# Synaptic-like transmission between neural axons and arteriolar smooth muscle cells drives cerebral neurovascular coupling

**DOI:** 10.1038/s41593-023-01515-0

**Published:** 2024-01-02

**Authors:** Dongdong Zhang, Jiayu Ruan, Shiyu Peng, Jinze Li, Xu Hu, Yiyi Zhang, Tianrui Zhang, Yaping Ge, Zhu Zhu, Xian Xiao, Yunxu Zhu, Xuzhao Li, Tingbo Li, Lili Zhou, Qingzhu Gao, Guoxiao Zheng, Bingrui Zhao, Xiangqing Li, Yanming Zhu, Jinsong Wu, Wensheng Li, Jingwei Zhao, Woo-ping Ge, Tian Xu, Jie-Min Jia

**Affiliations:** 1 School of Life Sciences, Fudan University, Shanghai, China; 2 Key Laboratory of Growth Regulation and Translation Research of Zhejiang Province, School of Life Sciences, Westlake University, Hangzhou, China; 3 Laboratory of Neurovascular Biology, Institute of Basic Medical Sciences, Westlake Institute for Advanced Study, Hangzhou, China; 4 Westlake Laboratory of Life Sciences and Biomedicine, Hangzhou, China; 5 Laboratory of Neurovascular Biology, School of Life Sciences, Westlake University, Hangzhou, China; 6 College of Artificial Intelligence and Big Data for Medical Sciences, Shandong Academy of Medical Sciences, Shandong First Medical University, Jinan, China; 7 Huashan Hospital, Shanghai Medical College, Fudan University, Shanghai, China; 8 Program in Speech and Hearing Bioscience and Technology, Harvard Medical School, Boston, MA USA; 9 Department of Neurosurgery, Huashan Hospital, Shanghai Medical College, Fudan University, Shanghai, China; 10 Brain Function Laboratory, Neurosurgical Institute of Fudan University, Shanghai, China; 11 Institute of Brain-Intelligence Technology, Zhangjiang Lab, Shanghai, China, Shanghai, China; 12 Shanghai Key Laboratory of Brain Function and Restoration and Neural Regeneration, Shanghai, China; 13 Shanghai Clinical Medical Center of Neurosurgery, Shanghai, China; 14 Department of Anatomy, Histology, and Embryology, School of Basic Medical Sciences, Fudan University, Shanghai, China; 15 Department of Anatomy, Histology, and Embryology, Research Center of Systemic Medicine, School of Basic Medicine, and Department of Pathology of the Sir Run-Run Shaw Hospital, The Cryo-EM Center, NHC and CAMS Key Laboratory of Medical Neurobiology, Zhejiang University School of Medicine, Hangzhou, China; 16 Chinese Institute for Brain Research, Beijing, Beijing, China

**Keywords:** Neuro-vascular interactions, Neurotransmitters, Molecular neuroscience, Neurophysiology

## Abstract

Neurovascular coupling (NVC) is important for brain function and its dysfunction underlies many neuropathologies. Although cell-type specificity has been implicated in NVC, how active neural information is conveyed to the targeted arterioles in the brain remains poorly understood. Here, using two-photon focal optogenetics in the mouse cerebral cortex, we demonstrate that single glutamatergic axons dilate their innervating arterioles via synaptic-like transmission between neural–arteriolar smooth muscle cell junctions (NsMJs). The presynaptic parental–daughter bouton makes dual innervations on postsynaptic dendrites and on arteriolar smooth muscle cells (aSMCs), which express many types of neuromediator receptors, including a low level of glutamate NMDA receptor subunit 1 (*Grin1*). Disruption of NsMJ transmission by aSMC-specific knockout of GluN1 diminished optogenetic and whisker stimulation-caused functional hyperemia. Notably, the absence of GluN1 subunit in aSMCs reduced brain atrophy following cerebral ischemia by preventing Ca^2+^ overload in aSMCs during arteriolar constriction caused by the ischemia-induced spreading depolarization. Our findings reveal that NsMJ transmission drives NVC and open up a new avenue for studying stroke.

## Abstract (web summary)

In the CNS, glutamatergic neurons directly control functional hyperemia via synaptic-like transmission onto arteriolar smooth muscle cells. Inhibiting this process reduces brain atrophy following cerebral ischemia.

## Main

Blood supply powers neural computations in the brain. Fluctuations in computational activity produce commensurate alterations in the regional cerebral blood flow (CBF) within seconds, termed NVC^1–3^. Aberrant NVC is involved in brain disorders, and our understanding of NVC is still evolving. The 130-year-old metabolic hypothesis^2^ explaining NVC has become increasingly controversial^4,5^, with cell-type-specific and neurotransmitter-mediated mechanisms recently emerging as the primary mechanism regulating CBF^1,6,7^. However, the molecular and cellular mechanisms underpinning this neural messenger delivery specificity remain largely unknown. Identification of the delivery strategy to contractile aSMCs is particularly important because they can powerfully and rapidly alter CBF^8,9^.

Local synaptic and spiking activity is thought to drive vessel dilation via the synthesis and diffusion of vasoactive factors. Vasoactive factors refer to substances that can influence the diameter and tone of blood vessels, examples of which include nitric oxide (NO), PGE2, and EET^1^. However, accumulating evidence has revealed this process to be controversial^10^. For instance, vessels frequently respond to stimuli that barely evoke neural activity in the surrounding tissue^10^; CBF persistently increases irrespective of a reduction in net neural activity when inhibitory GABAergic neurons are activated by optogenetics^11^. These findings indicate that the neural messenger delivery strategy to vascular cells is more complex than previously thought.

In addition to the local diffusion strategy, evidence of perivascular nerve endings closely associated with aSMCs has long suggested that synaptic-like transmission between the neural–vascular interface may also play a role in regulating blood flow in the brain^12–16^. However, this delivery mechanism has been a highly controversial topic and underestimated in textbooks and the literature^16^, mainly because of the general assumption that astrocytic endfeet entirely insulate the vasculature. Consequently, classical vasoeffective neurotransmitters such as serotonin, acetylcholine, dopamine and neuropeptide Y (NPY) are thought to diffuse and cross endfeet and then bind to their receptors expressed by aSMCs^14^. Whether these neurotransmitters otherwise mediate NVC through synaptic-like transmission remains to be established.

Recent advances in electronic microscopy revealed gaps in astrocytic endfeet overlying the area of capillaries covered by pericytes^17–19^, suggesting that axons may physically contact pericytes. Pericytes and aSMCs share embryonic origins and are collectively called mural cells. If axon terminals pass through endfoot gaps, they might be able to form NsMJs, like skeletal neuromuscular junctions^20^. With the current serial block-face scanning electron microscopy (SBF-SEM) for large-scale tissues, this hypothesis can be directly tested.

Extensive studies have demonstrated that the neurotransmitter glutamate has a major role in regulating CBF during neural activity^7,21^. While the glutamate-receptive intermediate cells such as interneurons, astrocytes and pyramidal neurons have been shown to modulate or partially mediate NVC by releasing NO, arachidonic acid derivatives and prostaglandins, the possibility for glutamate directly acting on aSMCs and relaxing arterioles has not been considered. Recent studies have shown that functional GluN1 expressed in the CNS endothelial cells (ECs) mediates NVC^22^. More recent mouse and human single-cell RNA-sequencing (RNA-seq) studies have reported that cerebral aSMCs and ECs express comparable levels of *Grin1* mRNA and aSMCs have the second-highest levels of *Grin1* mRNA abundance among brain cells, although still substantially lower than neurons^23,24^. This suggests that aSMCs per se may be glutamate perceptive, and if glutamatergic axons belong to a type of perivascular nerve endings remains to be established.

Acute ischemic stroke produces excitotoxicity due to excessive glutamate release^25–28^ and generates spreading depolarization (SD), leading to subsequent arteriolar constrictions^29^. However, excessive glutamate-caused overactivation of NMDA receptors in aSMCs has not been directly linked to arteriolar constrictions following SD. Furthermore, whether specifically blocking vascular NMDA receptor activation during excitotoxicity can relieve SD-caused vasoconstriction is completely unknown. Contractile aSMCs control vasomotor activity, depending on the cytosolic Ca^2+^ oscillation^30^. If aSMCs express Ca^2+^-permeable NMDA receptors, we hypothesize excessive glutamate may disrupt aSMC Ca^2+^ homeostasis, which may link to aSMC constrictive behavior during SD, expanding infarct size.

Here, we corroborate the synaptic-like transmission hypothesis, postulated half a century ago^16,31^, with the combination of multiple cutting-edge technologies. First, using three-dimensional (3D) reconstruction of volumetric correlative light electron microscopy (CLEM) and RNA-seq, we overcome the throughput issue of EM and unambiguously identify different types of NsMJ ultrastructure and versatile neurotransmitter receptors expressed by aSMCs. Second, using immunogold EM, we demonstrate NMDA receptors exist in aSMCs of mice, monkeys and humans. Third, in vitro and in vivo functional calcium imaging and whole-cell patch-clamp recordings revealed these NMDA receptors to be functional, producing both physiological and pathological consequences, depending on glutamate levels. Fourth, single-axon optogenetics and natural sensory inputs verified that glutamatergic axon NsMJ (Glu-NsMJ) transmission is at least as important as PGE2 diffusion in mediating NVC. Finally, mice with aSMC-specific NMDA receptor perturbation display attenuated vasoconstriction and improved outcomes after ischemic stroke.

## Result

### Presynaptic boutons contact with penetrating arteriolar SMCs

**Extended Data Fig. 1:**
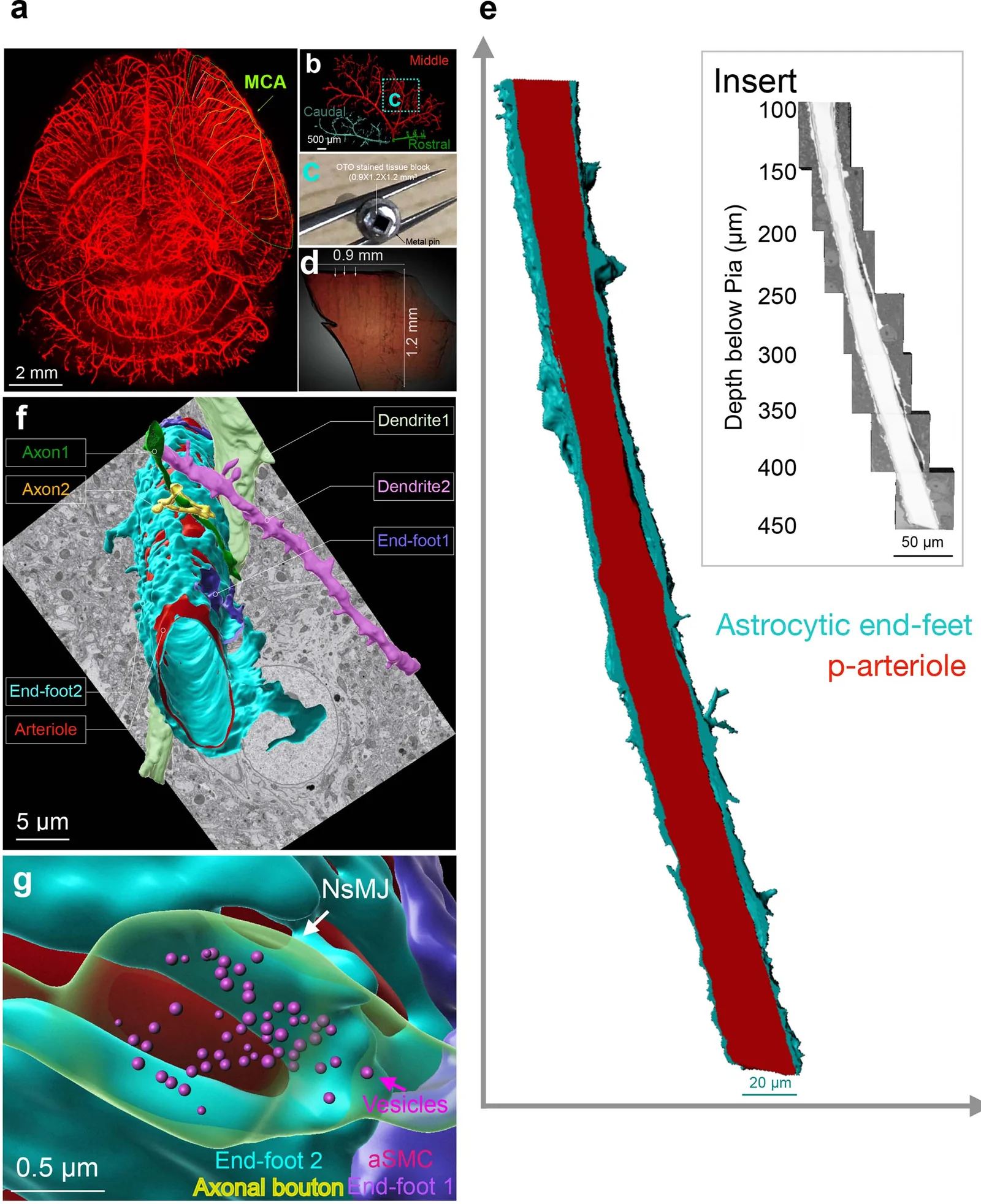
3D reconstruction of p-arteriole and perivascular cell components at the ultrastructural level. **(a)** Whole-brain arteriolar network of *SMACreER:Ai14* mouse. **(b)** The middle cerebral artery with three primary branches, namely rostral branch, middle branch, and caudal branch. **(c)** A 0.9 × 1.2 × 1.2 mm^3^ tissue block was collected from the somatosensory cortex as illustrated location by the cyan box labeled with c in b. This tissue block received OTO staining was immobilized on the metal pin and scanned by XRM **(d)**. **(e)** The opposite side of 3D reconstructed endfeet in Fig. 1b, which is the p-arteriole pointed by the most left white arrow in d. **(f)** 3D reconstruction of the 1^st^ branching arteriole in Fig. 1c, including 2 endfeet, 2 dendrites, and 2 axons. **(g)** A 3D-reconstructed NsMJ. A close-up perspective of f from an alternative angle reveals the presence of an NsMJ, showing axonal boutons that contain vesicles passing through astrocytic endfeet in direct contact with aSMCs.

To comprehensively assess direct contacts between axonal boutons and aSMCs, we examined serial ultrastructures for neurovascular units (spatial resolution of 5 × 5 × 75 nm^3^) of three penetrating arterioles (p-arterioles), downstream of the middle cerebral artery in a 0.9 × 1.2 × 1.2 mm^3^ mouse barrel cortex tissue (Extended Data Fig. 1a–d). We used 3View SBF-SEM to collect 3,000 ultrastructural images.

**Fig. 1:**
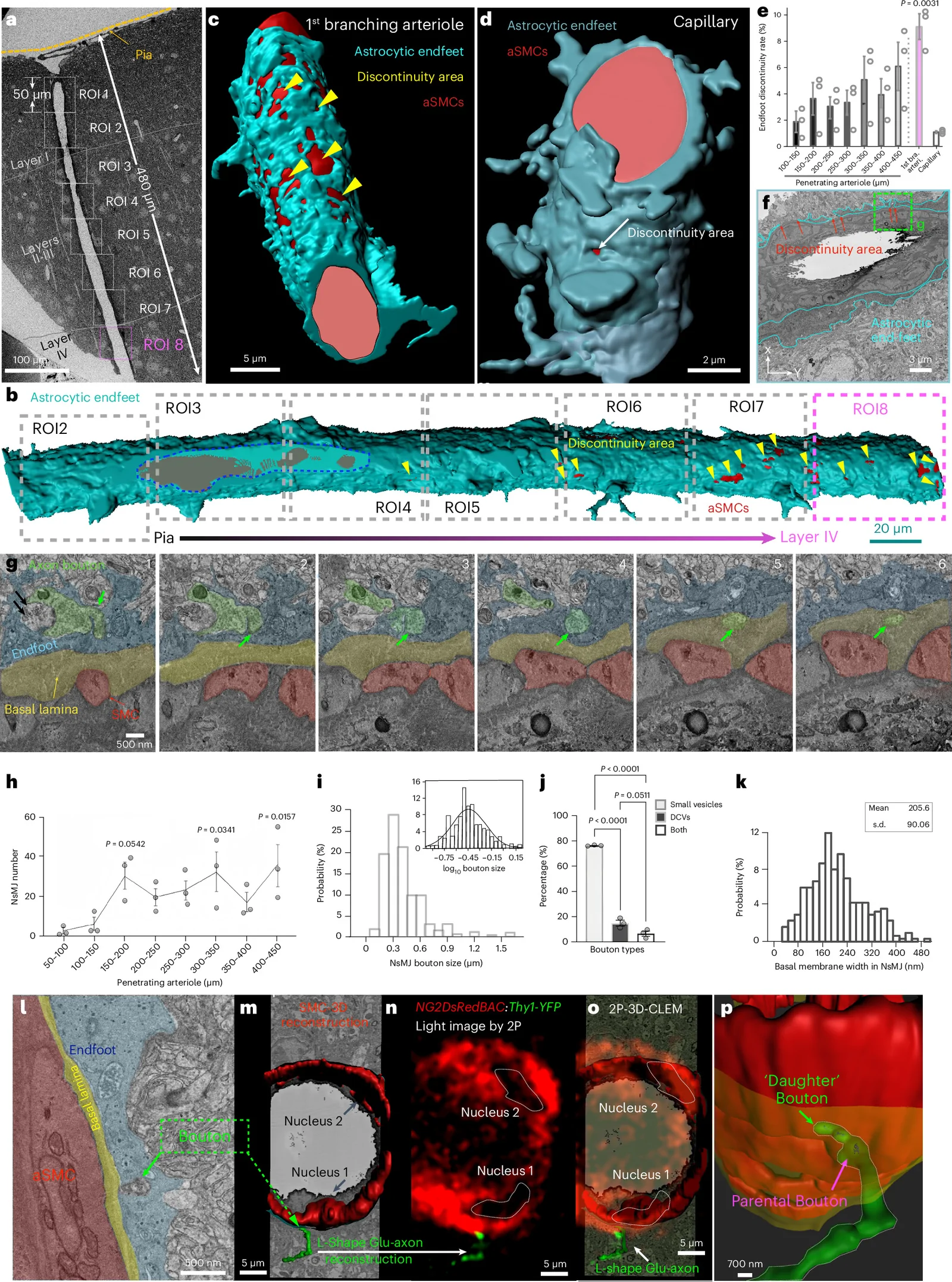
Axonal termini form dual innervations. **a**, SEM image of the barrel cortex with a p-arteriole (480 μm depth). **b**–**d**, 3D reconstruction of endfeet of the p-arteriole (ROI 2 to 8; **b**), the 1st branching arteriole (**c**) and a capillary deep in the cortex at 470 μm (**d**). **e**, The percentage of the discontinuity area for each ROI of p-arteriole, the 1st branching arteriole and the capillary. **f**, A single section from ROI 8. **g**, Six serial SEM images of the region in the green box in **f** (400 μm depth), with a section thickness of 75 nm. Axonal bouton is highlighted in green, and astrocyte endfeet are shown in blue. A typical asymmetric synapse is pointed by double black arrows in **g** (1). The daughter bouton is labeled by green arrows in **g** (1–6). **h**–**k**, Quantification analysis of NsMJ features. The average NsMJ number in each ROI (**h**; *n* = 499 NsMJs from ROI1–ROI8 of three p-arterioles), plot of NsMJ prejunctional bouton sizes with an inset of the calculation in log_10_ (**i**; *n* = 241 boutons), percentage of prejunctional bouton categories based on vesicle content (**j**; *n* = 499 NsMJs), and plot of the cleft distance of NsMJs (**k**; *n* = 297 basal membrane/junctional cleft measurements). The bouton-size and cleft-width measurements were obtained from subsets of NsMJs. **l**–**p**, Identification of Glu-NsMJ by CLEM, *n* = 1 NsMJ. **l**, A single SEM section from the insert in Extended Data Fig. 4e. **m**, 3D reconstruction of the axon in **l** and the two aSMCs. **n**, A single section of 2P images of PA-1, displaying two aSMCs and the associated L-shaped *Thy1* glutamatergic axon (YFP-positive). **o**, Superimposition of the CLEM images of **m** and **n**. **p**, High magnification and side view of the 3D reconstruction of Glu-NsMJ shown in **l**. Green arrows (prejunctional bouton); white arrows (L-shaped axon); magenta arrow (parental bouton); ‘nucleus 1’ and ‘nucleus 2’ (aSMC nuclei). Data are presented as the mean ± s.e.m. Statistical significance was determined by one-way analysis of variance (ANOVA) with a post hoc Bonferroni multiple-comparison adjustment (**e** and **h**) and nested, unpaired, two-tailed *t*-test (**j**). DCV, dense-core vesicle. Source data

We first determined the astrocyte endfeet discontinuous areas, which is a physical prerequisite because endfeet stand between aSMCs and neuropils^32^. The 3D-reconstructed astrocytic endfeet of half of the 450-μm-long p-arteriole, which was divided into seven regions of interest (ROIs; 50 μm for each) and spanned from the pia to layer IV, displayed a gradual increase in exposure areas (Fig. 1a–e; opposite-side view in Extended Data Fig. 1e). Each ROI was used as an object unit to quantify the endfoot discontinuity rate, which refers to the percentage of the total exposed area of the total arteriolar surface area. An increased number of more extensive exposed areas was found in the 1st branching arteriole that directly ramified from the p-arteriole in Fig. 1a, whereas only a small, exposed area in the capillaries (Fig. 1d,e). Because the p-arteriolar radii are larger than capillaries, we inferred a considerable proportion of the arterioles’ surface as exposed, which may allow a larger possibility for neural direct contacts.

Extended 3D reconstructions revealed direct contacts between aSMCs and neural somas, dendrites or axons (Extended Data Fig. 1f and Supplementary Video 1). Specifically, an axonal bouton that was originally located in ROI 8 (Fig. 1a,b) was found in a discontinuous area (the green box labeled ‘g’ in Fig. 1f). Serial images of this bouton revealed that its parental bouton had split into two parts (Fig. 1g). The larger part formed a conventional asymmetric synapse with a dendritic spine^33^, which likely is excitatory glutamatergic (Fig. 1g–1, black arrows). The collateral, smaller ‘daughter’ bouton was devoid of the endfoot, entered the basal lamina, and was juxtaposed to the aSMCs, which we termed NsMJ (Fig. 1g). An NsMJ may be seen from a specific angle by observing the exposed area of the aSMCs through the axonal bouton that contains synaptic vesicles (Extended Data Fig. 1g). Only 499 NsMJs were observed throughout these three p-arterioles and the number of NsMJs in each ROI was recorded, showing more NsMJs in layers II/III (ROIs 3–7) and IV (ROI 8) than in layer I (ROIs 1 and 2; Fig. 1h). This finding echoes the endfoot discontinuous rate results (Fig. 1e). These results clearly demonstrate that presynaptic boutons directly innervate the arteriolar wall without the endfeet interposed and concomitantly innervating postsynaptic neurons. Thus, unlike in the peripheral vascular system, parenchymal neural axons establish dual innervations with their targeted neuronal and nonneuronal cells.

To further investigate the ultrastructural features of NsMJs, we first characterized the boutons. Among these 499 prejunctional boutons, approximately 70% of them were between 0.3 and 0.6 μm, which are small sizes compared to conventional synaptic boutons (Fig. 1i). Presynaptic boutons can be classified into three subtypes based on synaptic vesicle features^34^. Accordingly, 76.4% of NsMJs were equipped with boutons that contained small, electron-lucent vesicles with a mean size of 50 nm, while 16% of NsMJs contained large or small dense-core vesicles and 7.6% possessed both classes (Fig. 1j and Supplementary Fig. 1). These results suggest that aSMCs may receive more frequent signals from neurons that release small-molecule neurotransmitters than those that release large-molecule neurotransmitters.

Secondly, electron contrast analysis revealed no postjunctional membrane feature in aSMCs (Supplementary Fig. 2a,b). A closer immunogold examination of postjunctional protein clustering in the aSMC membrane is presented later. Together, these ultrastructural results demonstrate that various types of axons establish direct innervation with thick extracellular matrix (ECM)-associated aSMCs in the brain.

**Extended Data Fig. 2:**
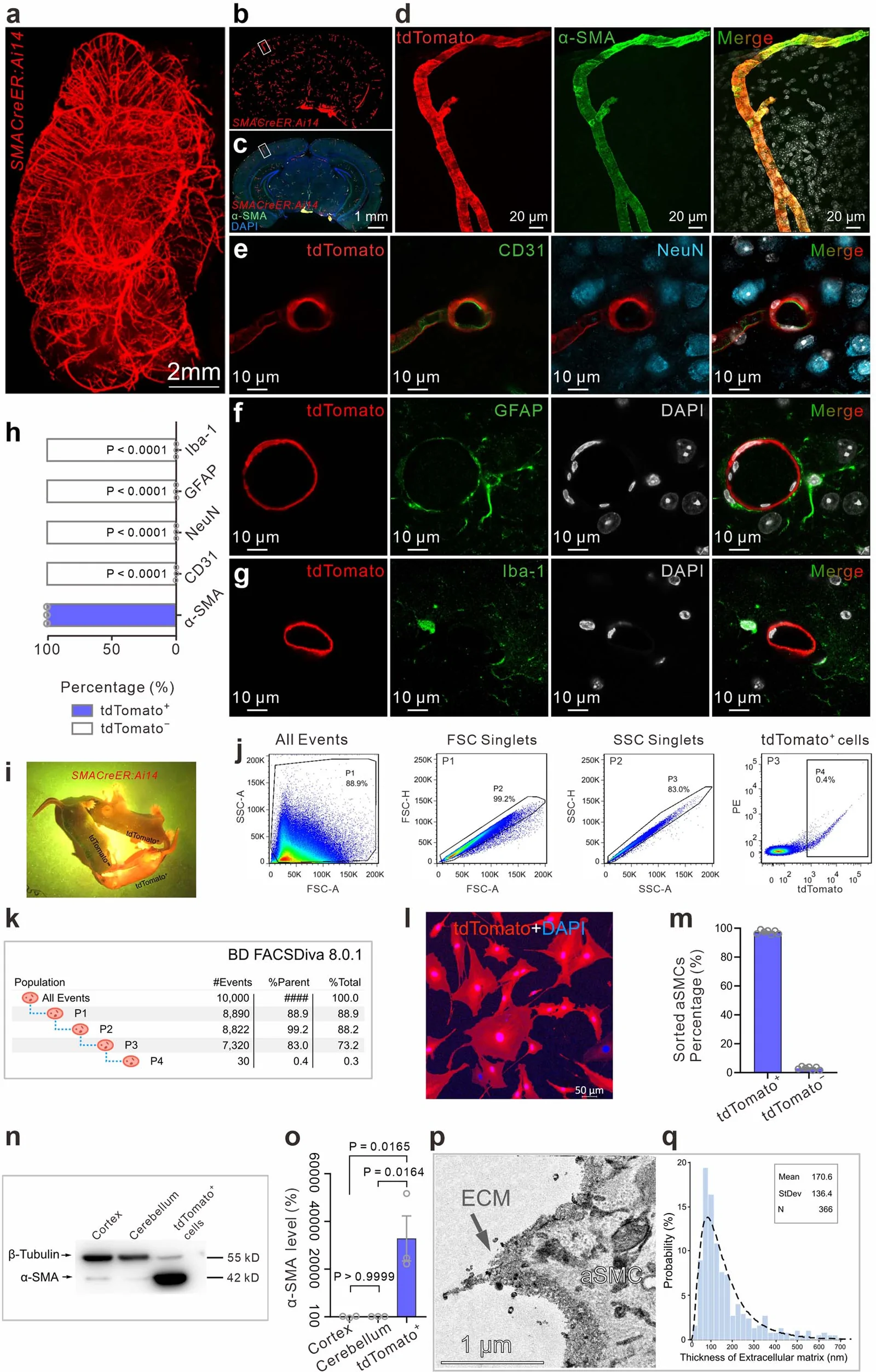
Demonstration of the efficiency and specificity of SMACreER promotor in labeling cerebral aSMCs. **(a-h)** The specificity of SMACreER driver line in labeling aSMCs. **(a)** 3D reconstructed light-sheet images of the cleared brain of *SMACreER:Ai14* mouse. (b-c) Low-magnification images of brain slice from *SMACreER:Ai14* mouse shown in single tdTomato signal **(b)** or multiple channels with anti-α-SMA and DAPI signals **(c)**. **(d)** High-magnification images of the region in the white box in b and c, with DAPI (white). **(e-g)** High-resolution immunofluorescent images of *SMACreER:Ai14* mouse brain slices co-stained with different cell markers, anti-CD31 **(e)**, anti-NeuN **(e)**, anti-GFAP **(f)**, anti-Iba-1 **(g)**, and DAPI **(e-g)**. **(h)** Statistic analyses of labeling of tdTomato^+^ and tdTomato^−^ cells with five different cell markers for d-g (N = 3 mice). Adult *SMACreER:Ai14* mice (2-3 months old) received tamoxifen intragastrically and were sacrificed one month later. **(i)** Fluorescent image of two tdTomato^+^ mice and one wild-type littermate at P3, which received tamoxifen starting at E18. **(j)** Representative FACS sorting of the parenchymal tdTomato^+^ aSMCs from cortices of pups in i. SSC stands for side scatter, FSC for forward scatter, A for area, H for height, and PE for phycoerythrin, an orange fluorescent protein. **(k)** The aSMCs with high tdTomato signal intensities were harvested via a FACS sorter, shown in the P4 gate. **(l)** Representative fluorescent image of the sorted aSMCs (tdTomato^+^), counterstained with DAPI, at DIV31 in vitro culture. **(m)** Quantification of the percentage of aSMCs versus total cell number (DAPI number) in l. N = 7 independent experiments. (n-o) Western blotting and quantification of aSMC marker α-SMA expressions in brain tissues and cells in l. **(p)** TEM image of the edge of the tdTomato^+^ aSMC derived from brain parenchyma. **(q)** The probability of ECM thickness along aSMC’s perimeter is quantified in p. Data are mean ± s.e.m.; one-way ANOVA after a post hoc Bonferroni multiple comparison adjustment **(h, o)**. Source data

Finally, we measured the gap between the bouton and aSMC membranes, which ranged from 40 to 480 nm with an average distance of 205.6 nm (Fig. 1k and Supplementary Fig. 2c). To determine if such a large junctional size is attributed to the intrinsic capability that aSMCs can generate large ECMs, we used transmission electron microscopy (TEM) to examine the ECMs of primary aSMCs isolated from the neonatal brains of *SMACreER:Ai14* mice (Extended Data Fig. 2 and Methods), from which meninges were removed to obtain parenchymal aSMCs. In this mouse line, tdTomato is exclusively expressed in aSMCs under the control of the *SMA* promotor without any nonspecific labeling of endothelia, neurons, astrocytes and microglia (Extended Data Fig. 2a–h). Additionally, the *SMACreER* promoter presents more specific labeling of aSMCs compared to other mouse line promoters including *SM22iCre*, *SMACre*, *Myh11CreER* and *PDGFRβCreER* (Supplementary Fig. 3). To obtain enough aSMCs, we expanded the sorted cell number in vitro for 14 d because original aSMCs only accounted for 0.3% of total brain cell populations (Extended Data Fig. 2k). After expansion, we achieved primary cultures of nearly all tdTomato positive (tdTomato^+^ Extended Data Fig. 2l,m) cells expressing a robust level of alpha-smooth muscle actin (α-SMA), a typical marker for aSMCs (Extended Data Fig. 2n,o). These aSMCs, in contrast to neurons, were intrinsically capable of producing thick ECMs, with an average thickness of 170 nm (Extended Data Fig. 2p,q), similarly to the size of the average junctional cleft (205.6 nm) in vivo (Fig. 1k). This suggests that a wide junctional distance is an inherent anatomical feature of NsMJs in vivo. Similarly, the conventional skeletal neuromuscular junctions have junctional clefts of up to 100 nm with thick muscle-derived ECMs^35^. Taken together, we defined the anatomical NsMJ as a synaptic-like neurovascular interface, consisting of three parts: a prejunctional membrane, a wide junctional cleft and a postjunctional membrane.

### Glutamatergic axons form NsMJs (Glu-NsMJs) with aSMCs

**Extended Data Fig. 3:**
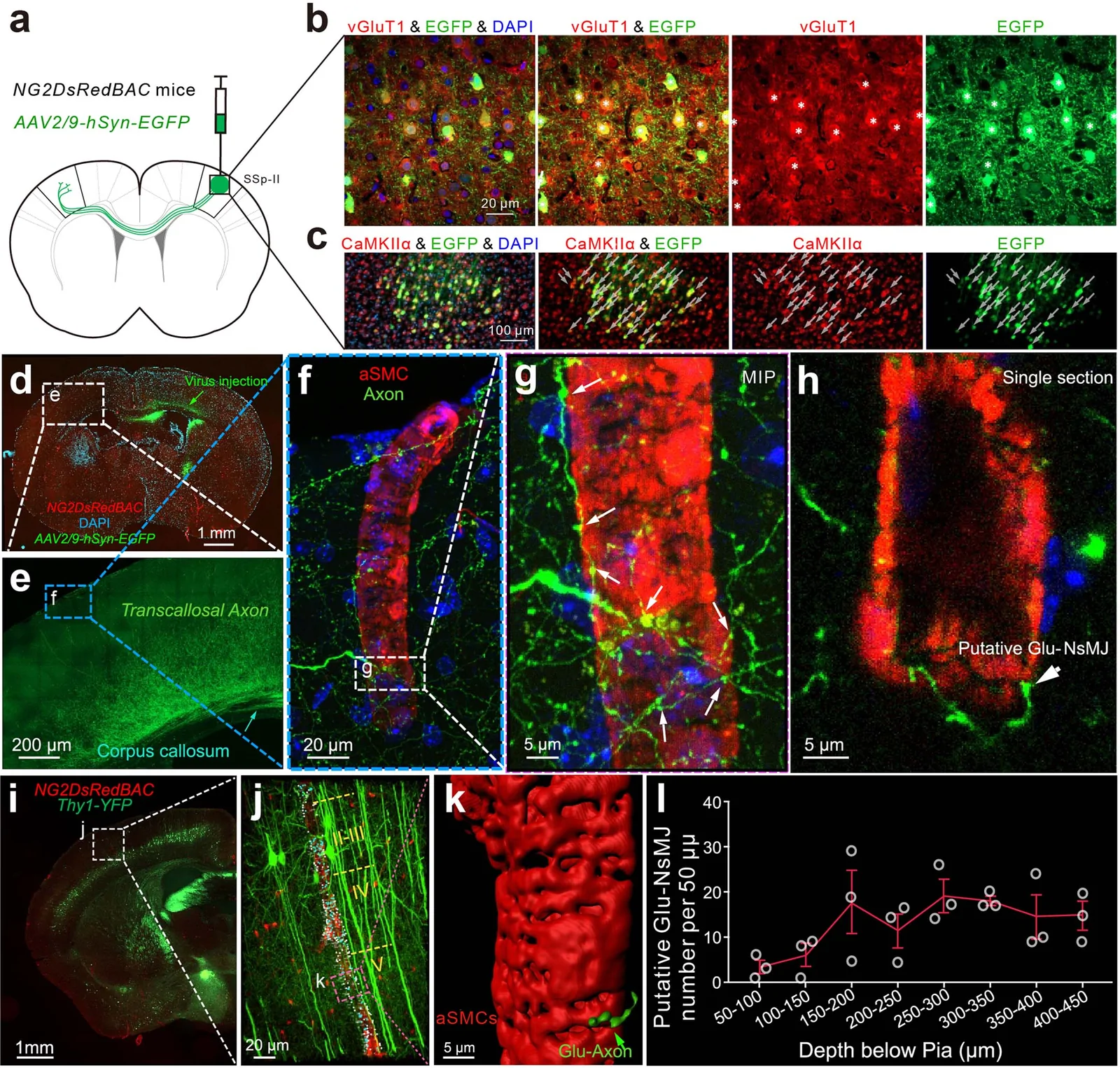
Glutamatergic axon wraps p-arterioles, forming putative Glu-NsMJs. **(a)** Schematic graph of virus stereoscopic injection to label glutamatergic neurons. **(b-c)** Immunoflourescent images of virus transduced EGFP^+^ neurons are positive for vGluT1 and CaMKIIα⊡ Asterisks and white arrows depict double-positive neurons. (d-h) Transcallosal axons innervated p-arterioles in the contralateral hemisphere. **(d)** The primary somatosensory layer V (SSp-II 5) neurons were transduced EGFP by injecting *AAV2/9-hSyn-EGFP* virus into *NG2DsRedBAC* Tg mice. **(e)** High magnification image of the projecting terminal location of axons. (f-g) Confocal zoomed-in images of p-arteriole wrapped by EGFP^+^ axons. **(h)** A single confocal magnifying section of g. **(i)** Brain slice of *NG2DsRedBAC:Thy1-YFP* mouse. **(j)** Magnified image shows the distribution of Glu-NsMJ along p-arterioles. Gray and cyan dots represent the putative Glu-NsMJs identified by YFP-positive axon boutons contacting DsRed-positive aSMCs. **(k)** 3D-reconstruction of a YFP-positive glutamatergic axon terminus and aSMCs. **(l)** Glu-NsMJ number along the p-arterioles per 50-μm length was analyzed (n = 3 PAs). Data are mean ± s.e.m. Source data

Encouraged by the findings above, we hypothesized that glutamatergic axons innervate arterioles and Glu-NsMJs exist in vivo. We first used confocal imaging to observe the transcallosal fibers in *NG2DsRed* mice that received AAV2/9-*hSyn-EGFP* virus injection in the barrel cortex, where most of the EGFP^+^ neurons were glutamatergic (Extended Data Fig. 3a–c). These axon fibers ran parallel or crossed p-arterioles transversely, with many nerve endings on aSMCs and likely forming putative Glu-NsMJs (Extended Data Fig. 3d–h). A similar distribution of the putative Glu-NsMJs in *Thy1-YFP:NG2DsRed* double transgenic mice according to results from 3D-EM quantification was found (Extended Data Fig. 3i–l).

**Extended Data Fig. 4:**
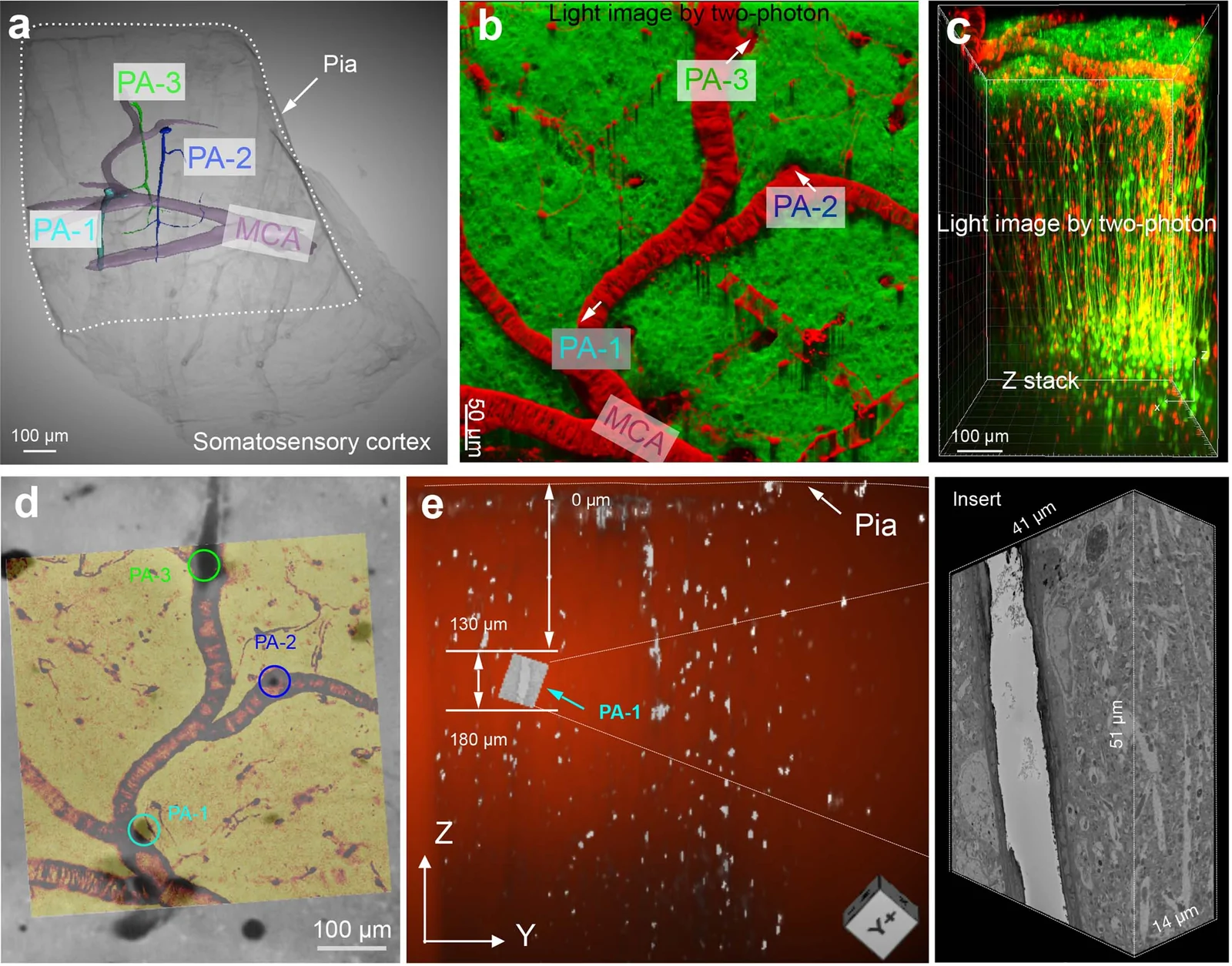
The original location of the Glu-NsMJ identified in Fig. 1l–p. **(a)** Semitransparent X-ray microscopy (XRM) image of the brain tissue block (0.9×1.2×1.2 mm^3^, the same block in Extended Data Fig. 1c and d) from *NG2DsRed:Thy1-YFP* mouse. Pseudocolors highlight the middle cerebral artery branch and its associated penetrating arteries (PA-1, PA-2 and PA-3). White dashed line outlines the pial surface of the somatosensory cortex (SSp-II). **(b)** Image arterioles in a by two-photon when the animal was alive. **(c)** Side view of the 3D reconstruction of two-photon images in b. **(d)** Top view of the superimposed two sets of 3D images of a and b. This superimposition was repeated with 2 tissue blocks independently from 2 *NG2DsRed:Thy1-YFP* mice. The circles mark each PA’s entry in the pia. **(e)** Side view of the 3D reconstruction of two-photon images from c. A segment of PA-1 below pia 130–180 μm is superimposed with a serial SEM image stack, magnified in Insert (n = 8 50-μm segments of a single PA-1).

Next, we used CLEM in 3D to correlate two sets of light and EM image data from the same *NG2DsRed:Thy1-YFP* mouse via the anatomical locations of three p-arterioles (PA-1, PA-2 and PA-3; Supplementary Fig. 4, Extended Data Fig. 4 and Methods), with the goal of verifying genuine Glu-NsMJs in the ultrastructure. In one segment of PA-1, 130 μm down from pia (Extended Data Fig. 4e), one aSMC was found to be directly opposite the vesicle-filled bouton, which is labeled ‘bouton’ in the green box (Fig. 1l). This particular bouton was reconstructed in 3D by using 38 successive SBF-SEM images, revealing an L-shaped axon that innervated one of the two aSMCs (Fig. 1m), which was the same axon acquired by two-photon (2P) microscopy (Fig. 1n). We then superimposed the two sets of images in Fig. 1o and found that the axon that innervated the aSMC in Fig. 1l,m was YFP positive and thus glutamatergic (Supplementary Video 2). The 3D reconstruction showed the glutamatergic prejunctional bouton was again a daughter bouton of the parental one (Fig. 1p). These structural data clearly demonstrate that the Glu-NsMJ exists in vivo.

### NsMJs in vitro with synaptic-like properties

**Fig. 2:**
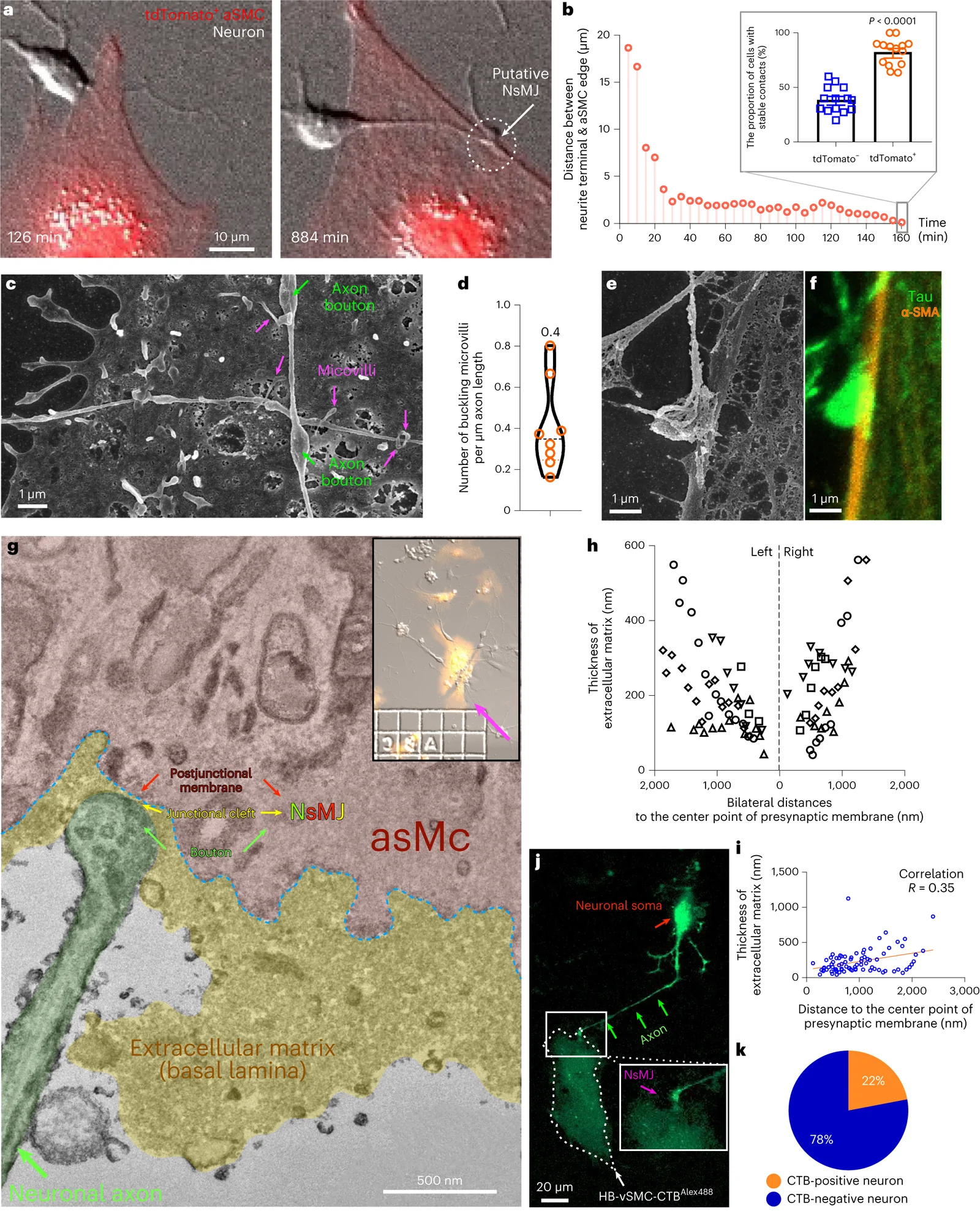
Recapitulated NsMJs in vitro. **a**, Bright-field and fluorescence microscopy images of the primary neurons and the tdTomato^+^ aSMCs, showing a putative NsMJ. **b**, The orange lollipop plot represents a single quantification for the example in **a**. The insert shows the percentages of cells that formed stable contacts with neurite terminals in the total tdTomato^−^ cells (*N* = 145 cells) or the total tdTomato^+^ cells (*N* = 150 cells; *n* = 14 field of views from 7 dishes of co-cultures). Data are the mean ± s.e.m.; nested, unpaired, two-tailed *t*-test. **c**,**d**, SEM of axons and aSMCs that protrude microvilli. **e**,**f**, CLEM of co-culture. SEM image (**e**) correlates with the confocal image (**f**), labeled with anti-Tau and anti-α-SMA. **g**, TEM image of the ultrastructure of an NsMJ on a tdTomato^+^ aSMC in th e insert labeled by the magenta arrow (Supplementary Fig. 6). The axonal bouton, ECM, aSMC cell membrane and aSMC are highlighted in light green, yellow, blue dashed lines and red. **h**, The thickness of aSMC ECM flanking NsMJs (*n* = 5 NsMJs, depicted by each shape of symbols). **i**, The correlation of ECM thickness of aSMC with the distance flanking NsMJs (*n* = 5 NsMJs). **j**, Fluorescence microscopy image of CTB^Alex-488^ of the co-culture at day 7 in vitro. Neuronal soma (red arrow); CTB^Alex488^-pretreated HB-vSMCs (white dashed line); CTB^Alex488^-positive axon (green arrows). The white frame with magenta arrow in the insert shows higher magnification of the NsMJ location. **k**, Pie graph of percentages of CTB^Alex488^-positive or negative neurons in co-cultured cortical neurons from three independent co-culture replicates. Source data

If NsMJs link neurons to aSMCs via synaptic-like connections, they should be recapitulated in vitro, unlike the artificial synapses that require overexpression of specific synaptic proteins^36^. Thus, we characterized the NsMJ in vitro neuron–aSMC co-culture system by utilizing various imaging techniques using neonatal *SMACreER:Ai14* mouse brains (postnatal day (P) 1–3). According to the experimental design in Supplementary Fig. 5a, freshly dissociated cortical neurons were added to previously seeded primary mixed cells (DIV 14) derived from brain parenchyma or leptomeninges, the latter containing pial arterioles. First, live-cell imaging recorded the entire process of axon terminals approaching and making stable contacts with both pial (Fig. 2a,b) and parenchymal (Fig. 2b and Supplementary Fig. 5b) aSMCs, which took approximately 2 h (Supplementary Video 3). Despite the relatively lower percentage of tdTomato^+^ cells (23%) compared to tdTomato^−^ cells (77%, which appeared to be fibroblast-like cells; Supplementary Fig. 5c–h), more tdTomato^+^ aSMCs established stable contacts with the approaching axons than the tdTomato^−^ (Fig. 2b). In addition, these contacts could still be observed when co-cultured cells were reimaged 14 d later (Supplementary Fig. 5i). Together, these data suggest that neurons have a higher tendency to form NsMJs with aSMCs.

Primary aSMCs are known to grow microvilli that are close projections of cytoplasm covered by the cell membrane. Interestingly, we observed that those microvilli can encircle neural axons with a spatial interval of 5 µm (Fig. 2c,d), as do myoblast and skeletal muscles^37^. Furthermore, we used correlative SEM to confirm that the above-observed contacts were axon marker Tau positive and aSMC marker α-SMA positive (Fig. 2e,f).

Finally, the TEM-CLEM (Supplementary Fig. 6) results revealed that the vesical-containing axonal bouton entered the sorted parenchymal aSMC’s ECM and apposed with the cell membrane, forming an NsMJ with a synaptic-like ultrastructural arrangement, similarly including a prejunctional-like membrane, junctional-like cleft and postjunctional-like membrane (Fig. 2g). Quantification analysis revealed an increasingly thicker ECM flanking the axon bouton and the thinnest in the center (Fig. 2h,i), reminiscent of NsMJs in vivo (Fig. 1g).

Finally, to directly assess if the NsMJ has the conventional synaptic features, we tested the retrograde transportation capability of the NsMJs by using cholera subunit B (CTB), a well-known neural tracer. We found that neurons that formed NsMJs became green when co-cultured with SMCs that were pretreated with Alexa Fluor 488-conjugated CTB (CTB-488; Fig. 2j). These pretreated SMCs were thoroughly washed, maintained in a CTB-free medium for an additional 48 h, and followed by washing again before primary neurons were added. These CTB-positive neurons accounted for 22% of the total neurons (Fig. 2k), suggesting a selectivity for the two types of cells to establish NsMJs.

### Cerebral aSMCs express various neuromediator receptor mRNAs

**Extended Data Fig. 5:**
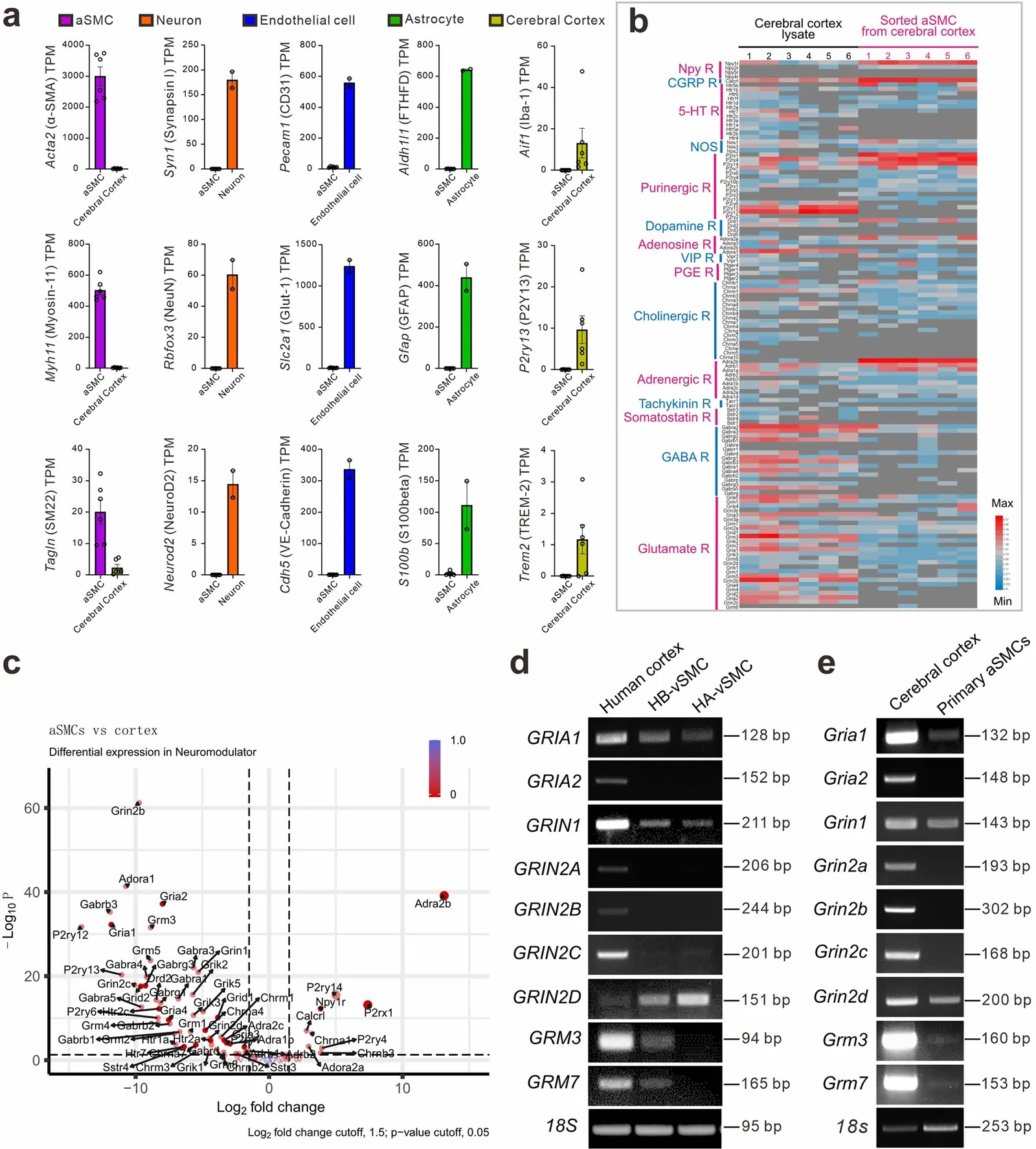
Cerebral aSMCs express a wide spectrum of receptors for neuron-derived signals. **(a)** Quantification of different cell marker expressions in the bulk RNA-seq data resulted from the sorted parenchymal aSMCs from *SMACreER:Ai14* mice. N = 6 mice. The transcriptomic data of neurons, astrocytes, and endothelia (n = 2 samples for each type of cells) were from the dataset in the study of Zhang Y., et. al. (2014, *JNS*, http://www.brainrnaseq.org/.) and those of aSMCs and cerebral cortex lysates (six samples for each) were generated in this study. Data are mean ± s.e.m. **(b)** Heatmap of relative gene expressions (red, high; light gray, median; blue, low; dark gray, no expression) of neuromediator receptors in cortical tissues and the sorted parenchymal aSMCs (N = 6 mice for each group). **(c)** Volcano plots presented DEGs amidst neuromediator receptor transcripts in aSMCs versus cortex. Log_2_ Fold change (FC) > 1.5 or < -1.5 (vertical dashed lines), and -log_10_ P-value > 1.30103 (*P* < 0.05, horizontal dashed line) was considered a significant difference. Tamoxifen was administrated to adult *SMACreER:Ai47* mice (2-3 months old) one month before cell sorting. (d-e) Representative RT-PCR results, the mRNA expressions of nine GluR subtype genes in human vSMCs lines **(d)** and primary cortical parenchymal aSMCs **(e)**. N = 4 independent experiments. Source data

To investigate the molecular prerequisite for junctional communications, we systematically profiled the neuromediator receptor gene expressions in aSMCs that were sorted from the enriched cerebral microvessels of adult brains of *SMACreER:Ai47* mice that received tamoxifen around 1 month before. Concerning contamination by ECs, which also express NMDA receptors^38^, we performed CD31 negative selection during cell sorting (Supplementary Fig. 7). A fraction of the same dissociated cortices was preserved, before the cell sorting experiment, as a control sample for the following bulk RNA-seq. The gene expression signatures of these samples were verified, indicating that we successfully sorted aSMCs without neuron and EC contamination (Supplementary Fig. 7d,e and Extended Data Fig. 5a).

In our transcriptomic analysis, 125 transcripts that encode neuromediator receptors for neuropeptide, neuromodulator or neurotransmitter, and enzymes for production of vasoactive agents were clustered and ranked according to their expression levels in aSMCs for each category (Extended Data Fig. 5b). The heat map indicated that parenchymal aSMCs expressed many types of neuromodulator receptors at different levels crossing different classes (Extended Data Fig. 5b). Differentially expressed gene analysis for the sorted aSMCs versus mouse cortices revealed that aSMCs not only highly expressed typical vasoactive receptors that have been demonstrated previously such as receptors for NPY, adrenaline and ATP^12,14^, but also expressed a range of subunits of glutamate receptors (GluRs) at very low levels (Extended Data Fig. 5b,c). Next, we used PCR with reverse transcription (RT–PCR) to confirm their subunit expressions by using two types of human cell lines (HA-vSMCs, HB-vSMCs) and primary mouse cerebral aSMCs. We found that, consistent with transcriptomic analysis, all the nine genes’ expression levels were lower in all types of SMCs compared to the positive control of mouse cerebral cortex and human brain tissue, with a pattern that NMDA receptor subunits *Grin1* and *Grin2d* had the highest expression levels, *Gria1* had median levels, *Grm3* and *Grm7* had low levels and *Grin2a*–*Grin2c* were undetectable (Extended Data Fig. 5d,e). Together, we conclude that more ionotropic and less metabotropic GluR subunits are expressed in cerebral aSMCs, which are conserved in human cells.

### GluN1 is on aSMCs’ abluminal side, conserved in mice and primates

We chose *Grin1* as the primary target for further investigation for three reasons. First, the GluN1 protein (encoded by *Grin1*) is the obligatory subunit in NMDA receptor multi-subunits complex^39^. Second, there are NMDA receptor-dependent human airway SMC contractions^40^. Thirdly, functional NMDA receptors in ECs have been reported to mediate NVC^22^, and the *Grin1* mRNA abundance in ECs is comparable to that in aSMCs^23,24^. Thus, we used multiple techniques to confirm the presence of *Grin1* mRNA and GluN1 protein in aSMCs.

**Fig. 3:**
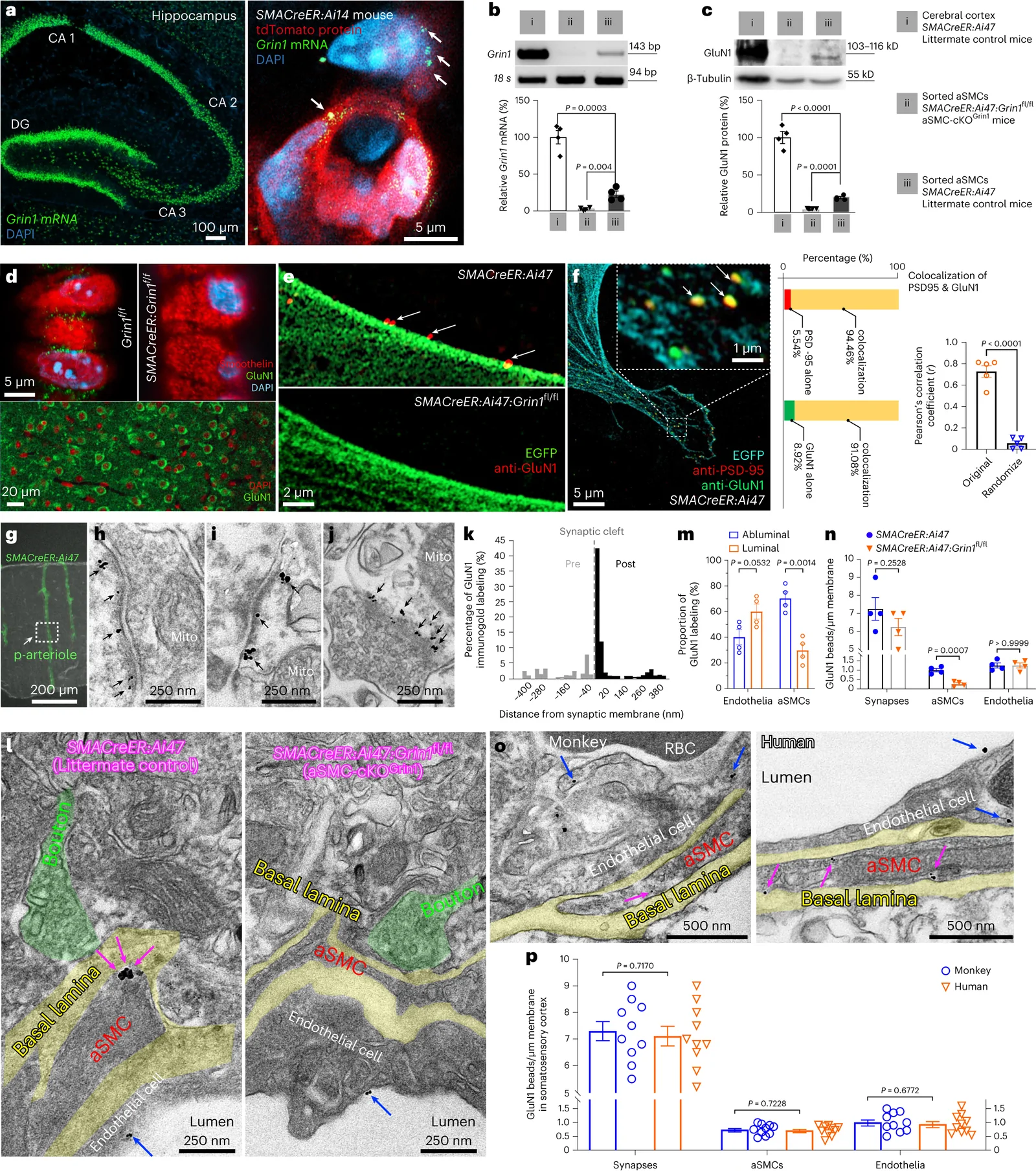
Abluminal expression of GluN1 in mouse and primate cerebral aSMCs. **a**, RNA scope images of *Grin1* mRNA in hippocampus (left). Airyscan image of a single slice of p-arteriole in barrel cortex (right) from a *SMACreERCreER:Ai14* mouse. *Grin1* mRNA foci (white arrows) are shown in aSMCs. Experiments were repeated with four mice. **b**,**c**, *Grin1* mRNA and protein in cortex and sorted cerebral aSMCs from littermate control (*SMACreER:Ai47*) and *aSMC-cKO*^*Grin1*^ (*SMACreER:Ai47:Grin1*^fl/fl^) mice. *N* = 4 mice. **d**, Immunofluorescence images of anti-GluN1. Top, isolated arterioles from control (*Grin1*^fl/fl^) and *aSMC-cKO*^*Grin1*^ (*SMACreER:Grin1*^fl/fl^) mice, aSMC marker smoothelin and DAPI. Bottom, cortex of an *aSMC-cKO*^*Grin1*^ mouse. *N* = 6 p-arterioles from two mice of each genotype. **e**, Membrane GluN1 puncta (red, indicated by white arrows) in the subcellular region of primary aSMCs from control (top) and *aSMC-cKO*^*Grin1*^ (bottom) mice, in both genotypes; aSMCs were labeled with EGFP. *N* = 3 repeated experiments. **f**, Colocalization analysis of GluN1 with PSD95 in primary EGFP^+^ aSMCs. *n* = 16 cells from three independent experiment replicates. **g**, Superimposed image of wide-field and fluorescence signals of brain slices from *SMACreER:Ai47* mice. The region (white box) underwent EM scanning. **h**–**j**,**l**,**o**, GluN1-gold beads in asymmetric synapses of mouse (**h**), monkey (**i**) and human (**j**), in aSMCs (magenta arrows), and endothelia (blue arrows) of p-arterioles from control mice (**l**, left, *SMACreER:Ai47*), *aSMC-cKO*^*Grin1*^ mice (**l**, right, *SMACreER:Ai47:Grin1*^fl/fl^), monkey (**o**, left) and human brain slices (**o**, right). *N* = 3 mice; one sample each for monkey and human. **k**, Histogram reflecting distances of GluN1 beads from presynaptic and postsynaptic membranes. **m**, Percentages of GluN1 immunoreactivity with abluminal or luminal distribution in aSMCs and endothelia of p-arterioles from control mice. **n**,**p**, Manual counting of GluN1 immunoreactivity in synapses, aSMCs and endothelia in brain slices from control and *aSMC-cKO*^*Grin1*^ mice (**n**), monkey and human (**p**). *N* = 4 mice,10 brain slices from one monkey and one human. In **a**, **d**, **g**, **h** and **k**–**n**, tamoxifen was intragastrically administered in adult mice at the age of 2 to 3 months, followed by euthanasia 1 month later. Data are the mean ± s.e.m.; nested, unpaired, two-tailed *t*-test (**b**, **c**, **f**, **m**, **n** and **p**). Source data

**Extended Data Fig. 6:**
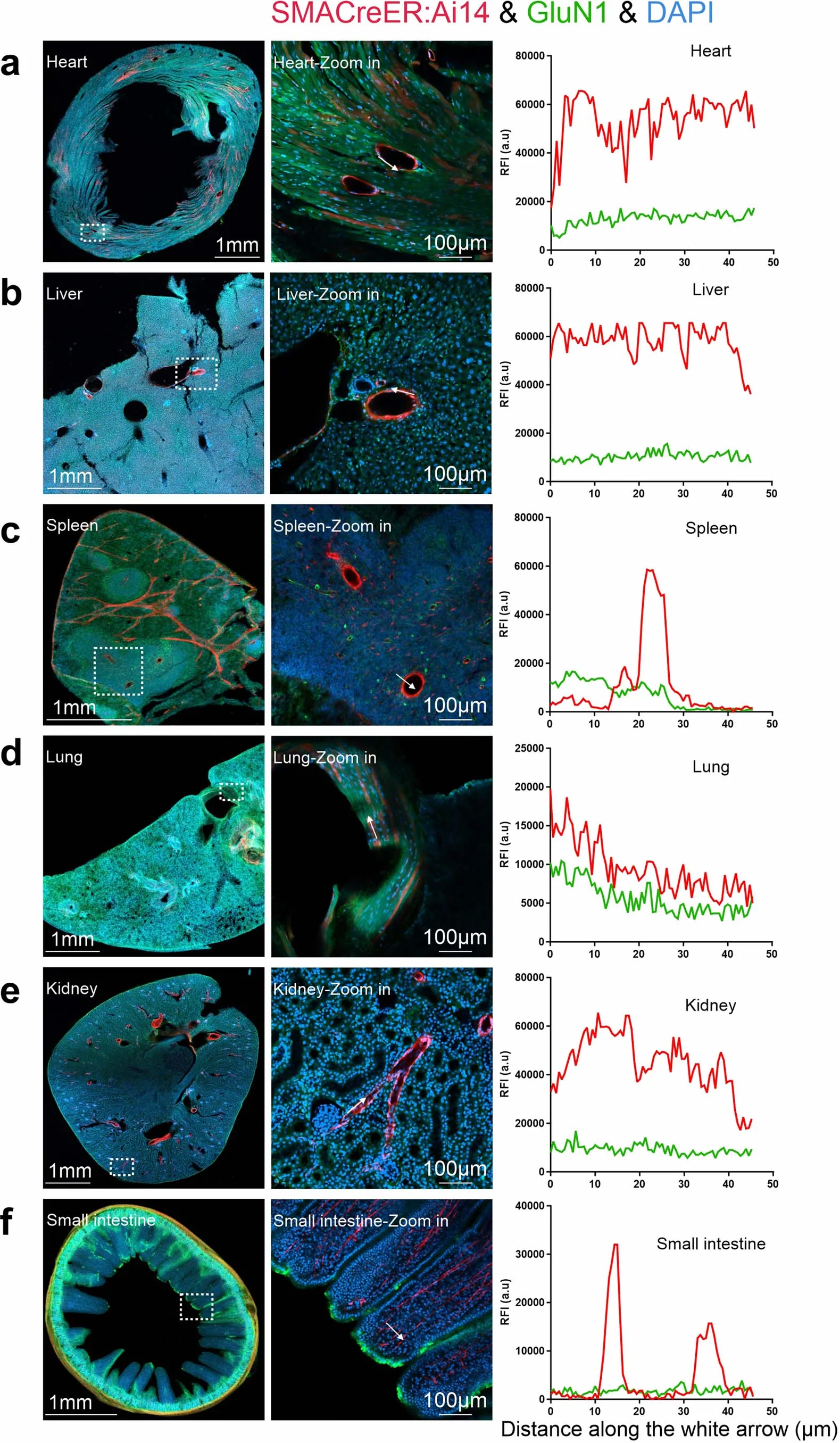
Vascular SMCs in peripheral organs do not express GluN1. **(a-f)** Left, Immunostaining of GluN1 in tissue sections from six organs (heart, liver, spleen, lung, kidney, and small intestine) from *SMACreER:Ai14* mouse, counterstained with DAPI. Middle, Zoomed-in images of the white boxed regions on the left. Right, Histogram of the red and green fluorescent intensities of the indicated sites in the Middle (white arrow). Tamoxifen was administrated to adult *SMACreER:Ai14* mice (2-3 months old) one month before collecting organs (N = 3 mice). Source data

The RNA scope detection system showed sparse *Grin1*-positive dots in the tdTomato^+^ aSMCs of the p-arterioles of *SMACreER:Ai14* mice as well as massive *Grin1* mRNA in hippocampus, validating the probe (Fig. 3a). To determine their relative expression levels in aSMCs compared to cortical tissue, we developed a selective aSMC GluN1 loss-of-function EGFP-labeling model by intercrossing *SMACreER:Ai47:Grin1*^f/+^ mice with *Grin1*^f/+^ mice or *Ai47:Grin1*^f/+^ mice, generating a triple transgenic mouse line *SMACreER:Ai47:Grin1*^fl/fl^, which, together with *SMACreER:Grin1*^fl/fl^, were named *aSMC-cKO*^*Grin1*^ mice. Their littermates *SMACreER:Ai47* with normal *Grin1* expression served as control mice because we had to rely on the Cre-dependent expression of reporter protein to perform cell sorting. Using RT–PCR and western blot, we found that *Grin1* mRNA and protein expression levels in aSMCs were lower than those of cortical tissues by fourfold to fivefold, and those were no longer observed in *aSMC-cKO*^*Grin1*^ mice (Fig. 3b,c and Supplementary Fig. 8a,b), while the total GluN1 protein levels in the whole-brain lysates were comparable between genotypes (Supplementary Fig. 8c,d), suggesting GluN1 expression in neurons was not affected. These results demonstrate GluN1 expression in aSMCs as well as confirmed specificities for the primers and anti-GluN1 antibody. Similar conclusions were drawn from the immunostaining of GluN1 in mouse brain sections (Fig. 3d) and primary cultured aSMCs (Fig. 3e). Besides, GluN1 puncta were found to localize in aSMC cell membrane (Fig. 3d,e). Notably, GluN1 expression was not observed in vascular SMCs in peripheral organs, such as heart, liver, spleen, lung, kidney and small intestine of *SMACreER:Ai14* mice (Extended Data Fig. 6), suggesting that only cerebral aSMCs receive glutamate.

Interestingly, recent studies have demonstrated that vascular SMCs express postsynaptic protein 95 (PSD95)^23,41–43^, a scaffold protein that binds to the GluN1 C terminus and cluster NMDA receptors in the cell membrane^44^. We not only confirmed this (Supplementary Fig. 9), but also found 94.4% of PSD95 colocalized with GluN1 in primary aSMCs (Fig. 3f) and vice versa. Immunofluorescence co-staining data revealed that, similarly to neurons where NMDA receptors are tethered to the postsynaptic cytoskeleton^45^, most GluN1 in aSMCs was associated with contractile α-SMA filaments (Supplementary Fig. 10), suggesting that NMDA receptors regulate contractile apparatus.

**Extended Data Fig. 7:**
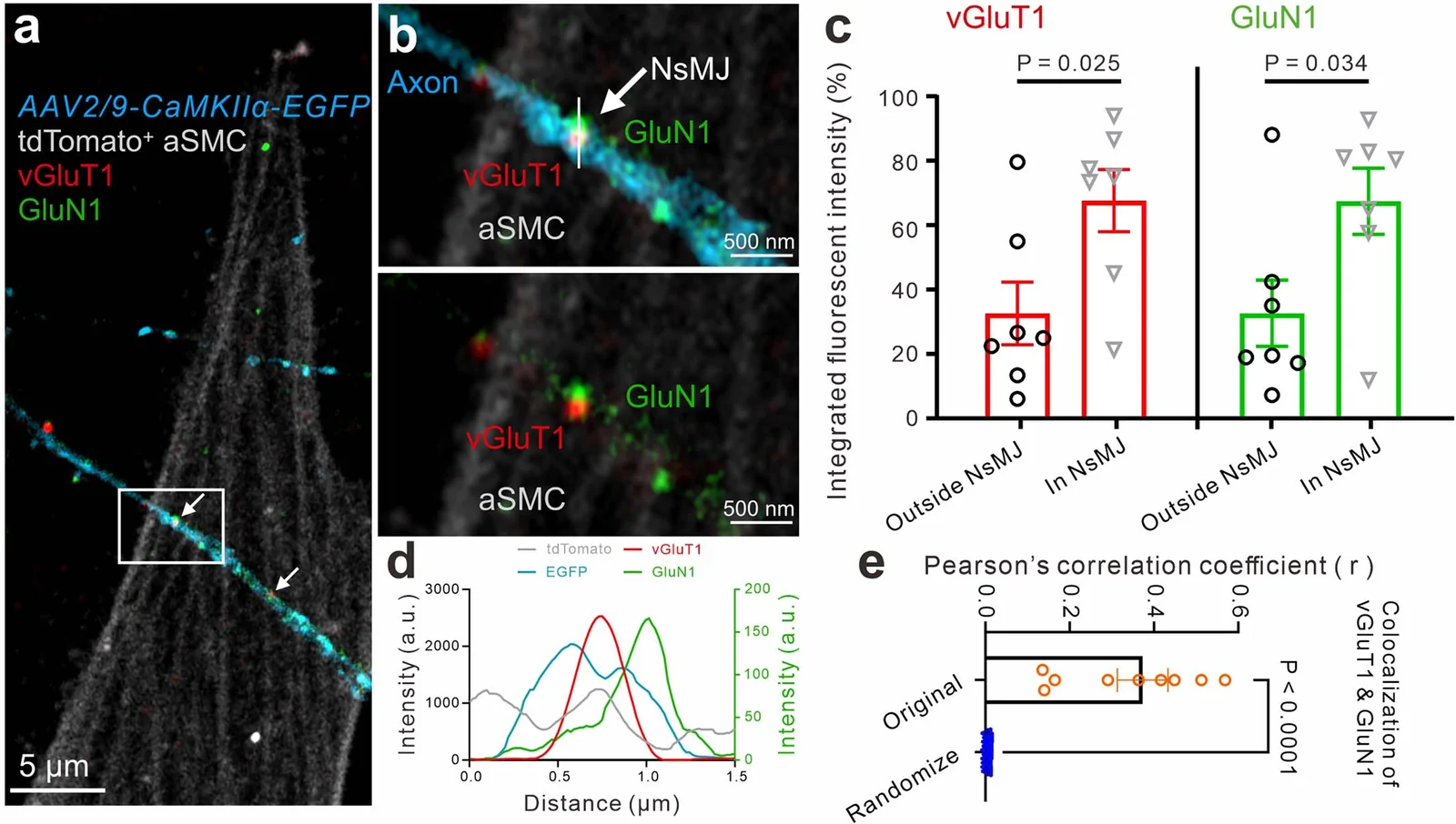
GluN1 puncta opposes with presynaptic marker vGluT1 of glutamatergic neurons in vitro co-cultures. **(a)** Immunofluorescent staining of glutamatergic presynaptic marker vGluT1 and GluN1 in coculture of neurons and aSMCs. Neuron infected by *AAV2/9-CaMKIIα-EGFP* virus. tdTomato^+^ aSMCs acquired from leptomeninges of *SMACreER:Ai14* mouse pups. **(b)** High-resolution Airycan confocal images of adjacent locations of GluN1 and vGluT1. The specific structure enriching molecular vGluT1–GluN1 complex is defined as NsMJ, indicated by white arrows. **(c)** The integrated fluorescent intensity of vGluT1 and GluN1 outside and in NsMJ respectively was quantified for a and b (N = 7 cocultures of axon and aSMCs). **(d)** The line histogram of vGluT1 and GluN1 proteins pixel intensity along the white line in b. **(e)** Co-localization analysis of vGluT1 with GluN1 (N = 9 cocultures). Data are mean ± s.e.m; nested, two-tailed *t*-test (c, e). Source data

To determine the ultrastructural localization of GluN1 in p-arterioles, we used the above-verified anti-GluN1 antibody to create an immunogold detection system that indicates the presence of a target epitope within a 30-nm radius (Supplementary Fig. 11). We chose the vessel segment in layers II–III in the *SMACreER:Ai47* mouse barrel cortex (Fig. 3g), where more NsMJs existed (Fig. 1h). The cortical synapses of mice, monkey and human were examined to confirm the GluN1 detection (Fig. 3h–k). We indeed found GluN1 beads in aSMC membrane, with an orientation toward the axonal boutons in control mice, but none were present in *aSMC-cKO*^*Grin1*^ mice (Fig. 3l). Likewise, prejunctional vGluT1 and postjunctional GluN1 were found to be preferentially colocalized in NsMJs in vitro (Extended Data Fig. 7). Notably, 70% of GluN1-positive gold beads were found on the abluminal side of the aSMCs (Fig. 3m), while GluN1 in ECs appeared to be equally distributed on the luminal and abluminal sides (Fig. 3l,m). While GluN1 immunoreactivity in the postsynaptic membrane and ECs remained comparable between both genotypic mice, they were substantially less in aSMCs in *aSMC-cKO*^Grin1^ mice (Fig. 3n). These results demonstrate that NMDA receptors preferentially reside in the abluminal membrane of the aSMCs. Notably, we observed GluN1 immunosignals crossing synapses, aSMCs and ECs in monkey and human brain slices (Fig. 3i,j,o), with similar orientations and relative abundances as in mice (Fig. 3n,p). Together, these data indicate that NMDAs in aSMCs with abluminal preferences are suitable for receiving glutamate directly from prejunctional neurons.

### Glu-NsMJ in vitro co-culture system is functional

**Fig. 4:**
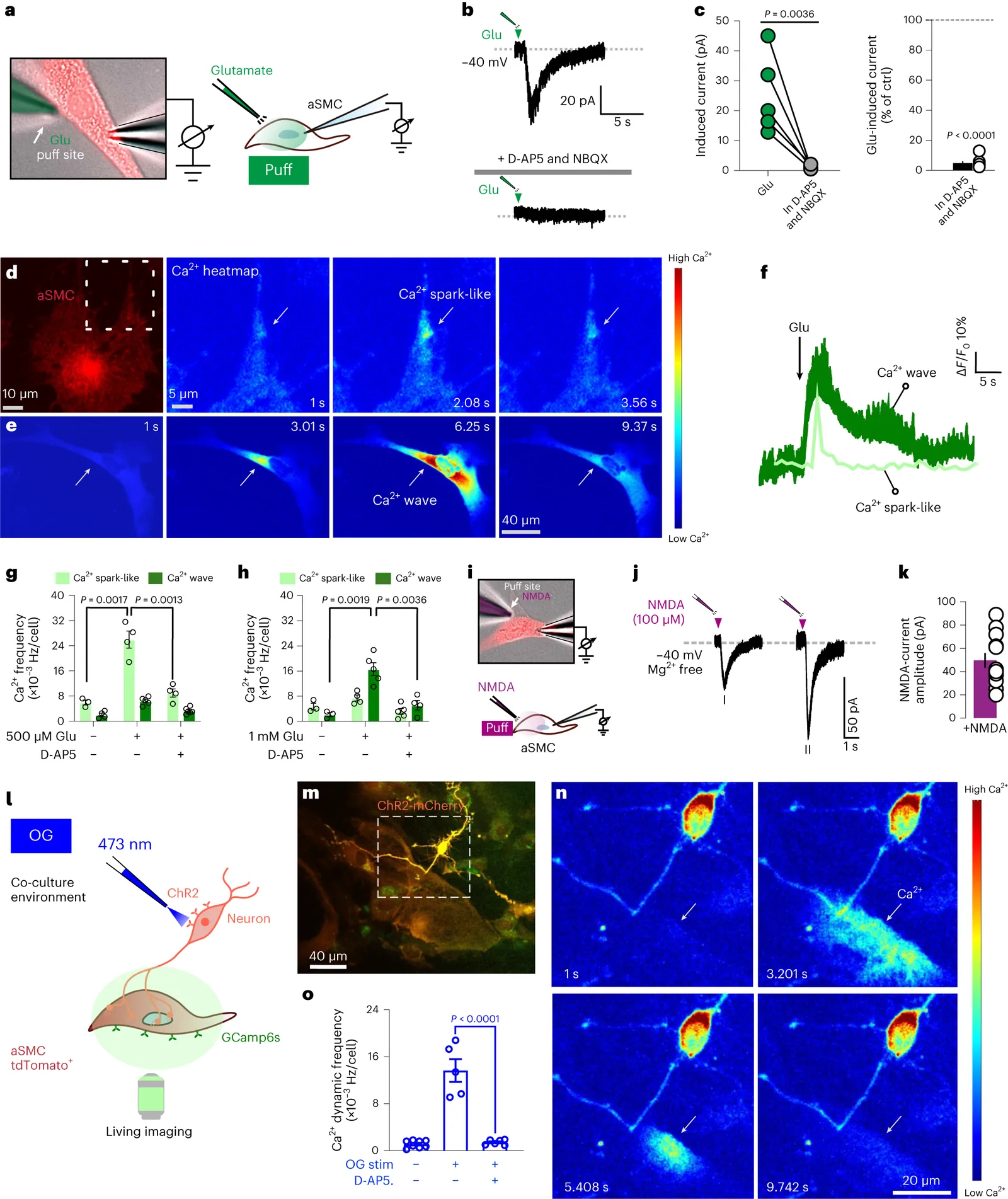
Functional assessments for NMDA receptors in primary aSMCs. **a**, Schematic showing the spatial arrangement of two electrodes, with the left one filled with glutamate (1 mM) for activating aSMCs through puff application, while the right one records membrane currents. **b**, A representative trace of glutamate-induced depolarizing currents at a holding potential of –40 mV in the absence (top) and presence (bottom) of D-AP5 and NBQX. **c**, The statistical analysis for **b** (*N* = 5 cells). **d**,**e**, Still frame images of Ca^2+^ spark-like (**d**) and Ca^2+^ wave (**e**) in aSMCs upon 500 μM (**d**) or 1 mM (**e**) glutamate stimulation (red, high; blue, low). **f**, Representative trace of Ca^2+^ spark-like kinetics and Ca^2+^ wave of **d** and **e**. **g**,**h**, Quantification of the frequencies of Ca^2+^ spark-like events and Ca^2+^ waves in aSMCs treated with vehicle (*n* = 4 cells), glutamate alone (**g**, 500 μM; **h**, 1 mM; *n* = 5 cells for each dose), or together with D-AP5 (*n* = 5 cells). These cells were collected from three independent experiments. The baseline F0 was determined by averaging 10 images without Ca2+ sparks. A Ca2+ spark was identified as a localized increase in ΔF/F0 greater than 0.2. Ca2+ waves were defined by an ΔF/F0 elevation >0.2 that propagated for more than 20 μm. **i**, Schematic illustration depicts the spatial configuration of electrodes, with the left one containing NMDA (100 μM) for puffing. **j**, Exemplary traces of NMDA-evoked currents at a holding potential of –40 mV under Mg^2+^-free artificial cerebrospinal fluid (aCSF) conditions. The first (left) and second (right) NMDA-induced currents were sequentially recorded on the same aSMC with intermediate aCSF washing. **k**, Quantification for **j** (*N* = 12 cells). **l**, Model diagram shows neural optogenetics (OG) and responding GCamp6 dynamic signals in co-culture. **m**, Representative live image of a ChR2 and GCamp6 double-positive neuron connecting with a GCamp6 and tdTomato double-positive aSMCs; *n* = 6 co-cultures. **n**, Sequential Ca^2+^ images of aSMCs upon OG. **o**, Quantitative analysis of the effect of D-AP5 on Ca^2+^ dynamics in aSMCs during OG, as shown in **n** (*N* = 8 cells without OG; 5 cells with OG; 6 cells with OG plus D-AP5; cells were collected from three independent experiments). Data are the mean ± s.e.m; nested unpaired two-tailed *t*-test (**c** and **o**), one-way ANOVA with Bonferroni multiple-comparison post hoc tests (**g** and **h**). Source data

We first tested if NMDA receptors in aSMCs are functional. The electrophysiological and intracellular Ca^2+^ dynamic responses of primary aSMCs to exogenous glutamate were investigated using whole-cell patch-clamp recordings and live imaging of GCamp6s signals (Fig. 4a–k). We observed that a short puff (~1 s) of glutamate evoked a substantial inward current in aSMCs when holding the resting membrane potential of −40 mV (Fig. 4a–c), which was largely suppressed in the presence of GluR antagonists, d-(-)-2-amino-5-phosphonopentanoic acid (D-AP5) and NBQX (Fig. 4b,c). This indicates a glutamate-induced current and functional GluRs in aSMCs. Meanwhile, we observed that low-dose glutamate preferentially increased the frequency of local Ca^2+^ spark-like dynamics that did not propagate (Fig. 4d). In contrast, a high concentration of glutamate was prone to induce more Ca^2+^ waves, which propagated (Fig. 4e). Both types of Ca^2+^ activity were attenuated by NMDA receptor blocker D-AP5 (Fig. 4f–h and Supplementary Video 4).

We further discovered that ionotropic NMDA receptors were predominantly expressed on aSMCs, and activation of these receptors could evoke receptor-mediated currents (Fig. 4i–k). We repeatedly detected NMDA currents in zero magnesian medium when we puffed NMDA to aSMCs, followed by washing and a second puffing (Fig. 4i–k). aSMCs from leptomeningeal pial and parenchymal arterioles responded similarly to glutamate and NMDA stimuli. Because it has been shown that different forms of Ca^2+^ transients correlate SMC relaxation and constriction differently^46,47^, these results suggest that aSMC responses to glutamate are dose dependent and may exhibit differential consequences with respect to contractility.

Next, in the co-culture system, neurons were overexpressed with GCamp6s, and the light-activated nonselective cation channel rodopsin-2 (ChR2) was tagged with mCherry, while tdTomato^+^ pial aSMCs expressed GCamp6s only (Fig. 4l,m). Epifluorescence stimulation of neural ChR2 substantially increased the frequency of Ca^2+^ events in aSMCs that were targeted by axons (Fig. 4n,o and Supplementary Video 5), and this enhancement was blocked by D-AP5 in co-culture with pial or parenchymal aSMCs (Fig. 4o). Thus, functional Glu-NsMJs that depend on NMDA receptor activity can be recapitulated in vitro.

### Glu-NsMJ transmission is required for arteriolar dilations

**Extended Data Fig. 8:**
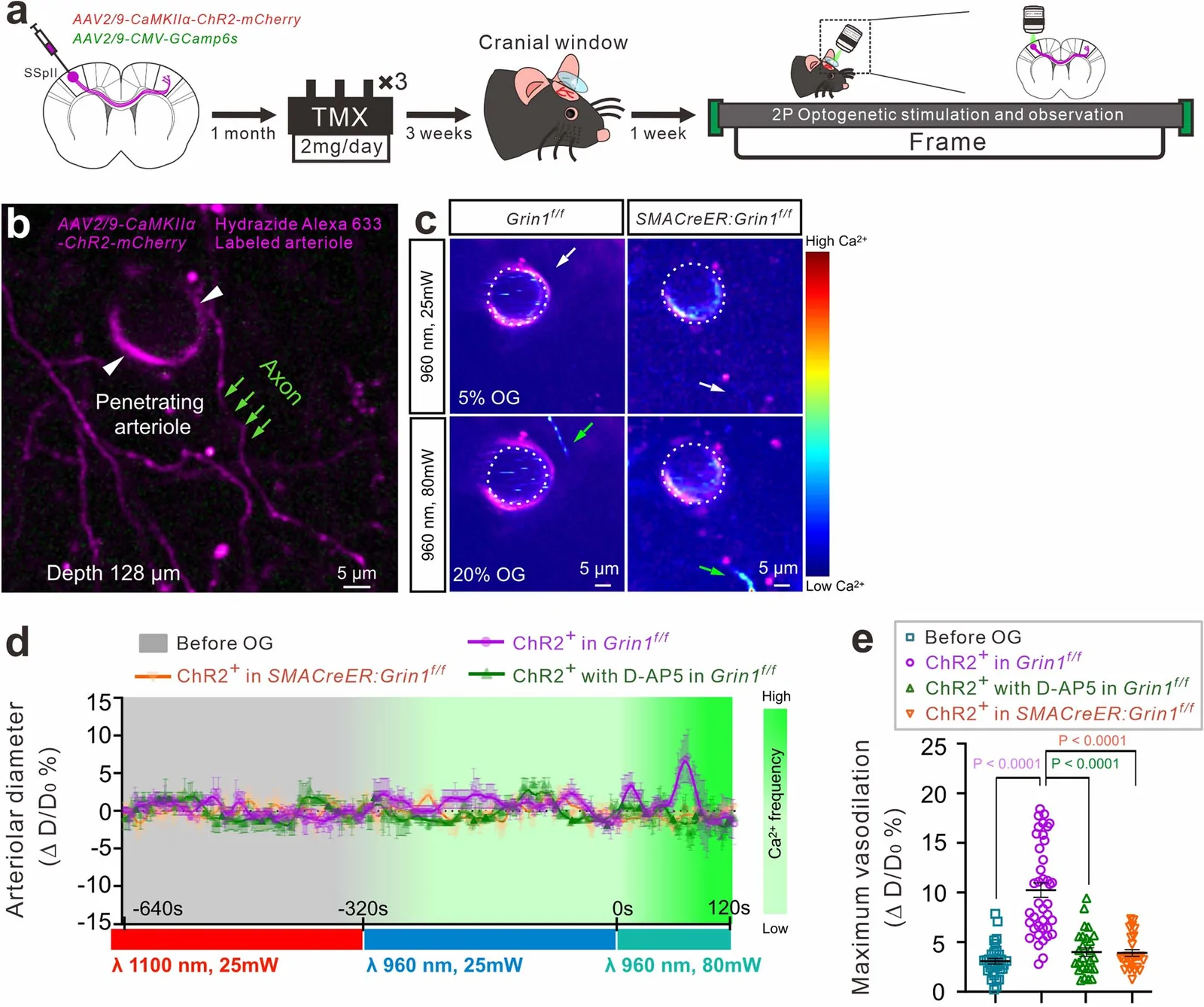
Prolonged, broad two-photon optogenetic activation of glutamatergic neurons dilates penetrating arterioles, depending on aSMC’s NMDA receptors. **(a)** Schematic graph of the experimental workflow of broad 2 P optogenetics **(b)** Two-photon image of a p-arteriole (labeled with Hydrazide Alex 633, white arrows) at a cortical depth of 128 μm and surrounding ChR2-mCherry positive glutamatergic axons (green arrows). **(c)** Still frame images of p-arterioles before and during photostimulation in the littermate control (*Grin1*^*f/f*^) and *aSMC-cKO*^*Grin1*^ mice (*SMACreER:Grin1*^*f/f*^). The GCamp6s intensity further enhanced in the same axons when illumination power switched from 25 mW (white arrow) to 80 mW laser (green arrow). The white dashed lines outline the basal arteriole calibers. **(d)** Time course of changes in p-arteriolar diameter in response to 1100-nm laser at 25 mW and the following 960-nm laser with increasing power in c. **(e)** Maximum dilation amplitudes in c (magenta, n = 8 p-arterioles from 6 mice) or cistern magna injection of D-AP5 (100 μM, green, n = 4 p-arterioles from 3 mice) in control mice or *aSMC-cKO*^*Grin1*^ mice (orange, n = 6 p-arterioles from 5 mice). Data are shown as the mean ± s.e.m.; nested, one-way ANOVA after a post hoc Bonferroni multiple comparison adjustments **(e)**. Source data

To directly assess if Glu-NsMJs regulate the diameter of the targeted p-arterioles in vivo, we used 2P neural optogenetics with two types of modes—broad or focal stimulations. Broad photostimulation by using the previously verified power levels that are effective as well as causing no injury^48^ was first performed to confirm successful activation of glutamatergic transcallosal axon terminals that coexpressed ChR2-mCherry and GCamp6s in our 2P microscopy (Supplementary Fig. 12a–d, Supplementary Video 6 and Methods) and no tissue injuries were found after photostimulation (Supplementary Fig. 12e). Consistent with previous studies^49^, we found that the brief activation of glutamatergic axons by broad 2P scanning sufficiently drove p-arteriole dilations (Extended Data Fig. 8a–e). The concerns of heating-induced vasomotion were excluded because we did not observe vascular responses when ChR2 was absent (Supplementary Fig. 13).

**Fig. 5:**
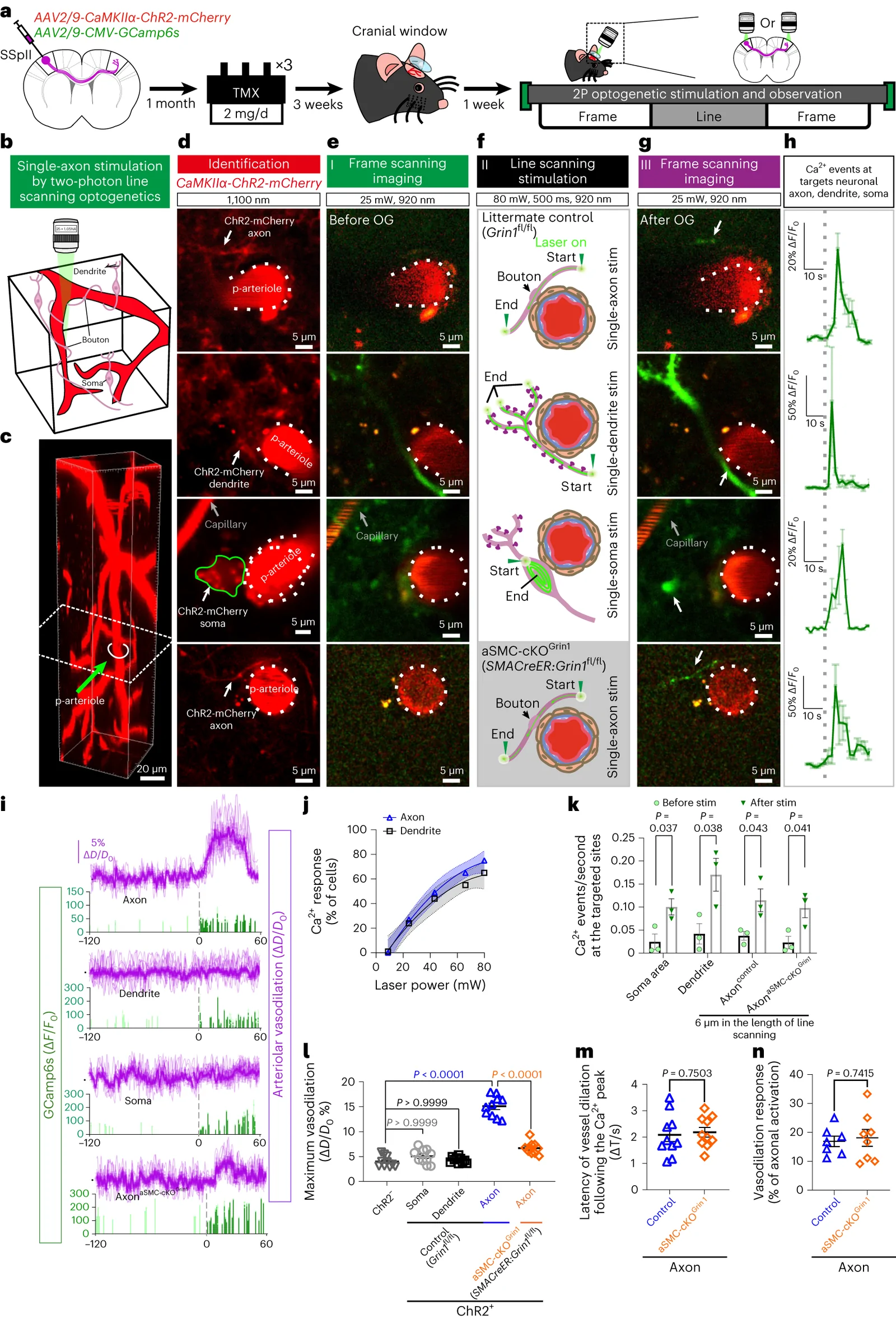
A single glutamatergic axon optogenetic activation drives vasodilation, depending on Glu-NsMJ transmission. **a**, Experimental flow of virus injection, tamoxifen administration, cranial surgery and 2P optogenetics. **b**, Schematic illustration of focal 2P optogenetics used to stimulate different parts of glutamatergic neurons expressing ChR2-mCherry and GCamp6s. **c**, Side view of p-arteriole with the stimulation location depicted. **d**, Focal images of stimulation planes using a 1,100-nm laser. Plasmatic rhodamine labels p-arterioles (white dashed lines outline the original diameters before stimulation) and mCherry signals indicate ChR2^+^ neural compartments. **e**–**g**, Single neurite and soma optogenetics in control and *aSMC-cKO*^*Grin1*^ mice, with three steps (**I**, observation; **II**, stimulation; and **III**, observation). Neural GCamp6s signals and p-arteriole diameters before (**e**) and after (**g**) stimulation. **f**, Illustration of line or spiral 2P stimulation paradigms. Neuronal compartments in purple, aSMCs in flesh and outlined in brown, ECs in blue, lumen in red, and 2P line scanning routes indicated by green start and end points connected by the green line. **h**, Ca^2+^ events recorded immediately after photostimulation (gray dashed line). **i**, Temporal Ca^2+^ events and p-arteriole diameter changes before and after photostimulation in control and *aSMC-cKO*^*Grin1*^ mice (gray dashed lines indicate a 500-ms, 80-mW power photostimulation,). *N* = 5 mice, 7 arteriole-and-neural-component sets. **j**, The percentage of calcium response in axons and dendrites following increasingly stronger 2P optogenetics. The blue and black lines, along with the error bands, represent the quadratic fitted curves with a 95% confidence level. **k**, Quantification of Ca^2+^ event frequency before and after photostimulations in targeted regions (*n* = 3 mice, 3 somas or neurites in total). **l**, Maximum changes in arteriolar dilation in littermate control and *aSMC-cKO*^*Grin1*^ mice (*n* = 5 mice, 10 arterioles in total for each group). **m**, Latency of vasodilation following the peak of Ca^2+^ signal (*n* = 5 mice, 10 axons per genotype). **n**, Probability of vasodilation following successful neural activations. (Control, *n* = 7 mice, 27 stimulated arterioles total; *aSMC-cKO*^*Grin1*^, *n* = 8 mice, 36 stimulated arterioles total). Data are the mean ± s.e.m.; nested, unpaired, two-tailed *t*-test (**k**, **m** and **n**); one-way ANOVA with post hoc Bonferroni multiple-comparison adjustment (**l**). Source data

Broad photostimulation inevitably activated many postsynaptic neurons other than glutamatergic neurons, which may also contribute to vasodilation. Thus, we developed a focal stimulation paradigm to precisely activate single axons surrounding p-arterioles (Fig. 5a–d and Methods). Under anesthesia, basal Ca^2+^ events were sparse (Fig. 5e). Following a line scanning stimulation (Fig. 5f), Ca^2+^ events were immediately and reliably induced (Fig. 5g,h), demonstrating the success of the focal mode and showing an 80% success rate at 80 mW and potentiation in neural activity that can last up to 1 min (Fig. 5i,j). This occurred only at the targeted sites but not the neighboring regions except for soma activations, demonstrating the precision and specificity of stimulation (Fig. 5k and Supplementary Fig. 14).

Notably, a single glutamatergic axon activation induced a rapid, prominent p-arteriole maximum dilation of up to 15.1% ± 0.7% (Fig. 5i,l), whereas, in mice with no ChR2, we only observed a maximum 4.0% ± 0.3% change in diameter (Fig. 5l). Conversely, the activation of glutamatergic postsynaptic sites (dendrites and somas), which is presumed to release vasoactive byproducts, such as PGE2, did not induce vasomotion in this context (Fig. 5i,l). This result demonstrates that single glutamatergic axon activation has a potent dilatory effect, which may be independent of diffusing vasodilators.

**Extended Data Fig. 9:**
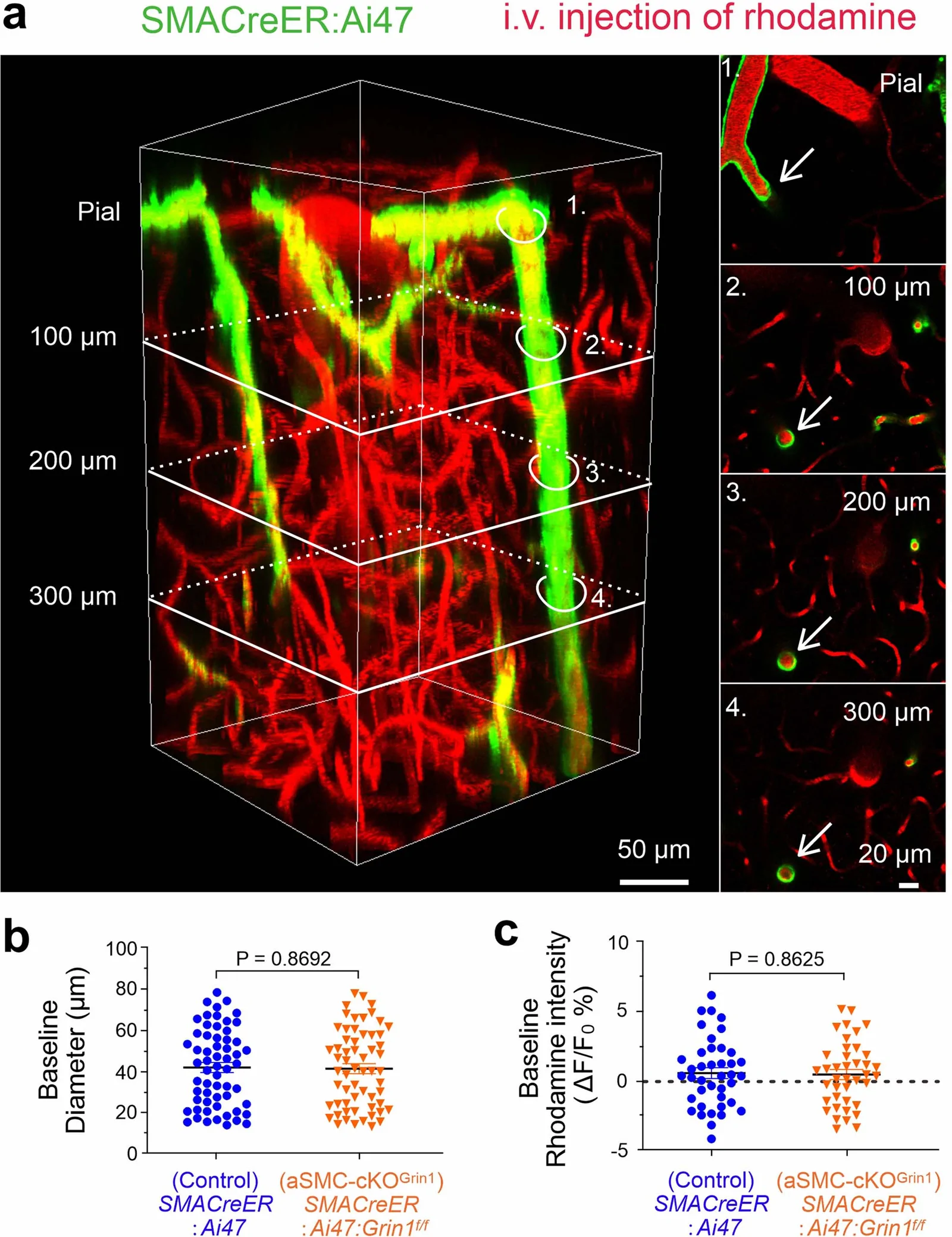
Comparable basal diameter of p-arterioles between littermate control and *aSMC-cKO*^*Grin1*^ mice. **(a)** Side view of three-dimensional volume image of a two-photon-imaged site in *SMACreER:Ai47* mouse barrel cortex. The lumen of all vessels is filled with i.v. injected rhodamine and aSMCs are genetically labeled by EGFP. The deepest imaged bin is at 300 μm. White slices correspond to z cross-sections shown per depth. **(b-c)** The baseline of basal diameter and blood flow. Tamoxifen was given to adult mice three weeks before cranial surgery, and live imaging was performed one week after cranial surgery in panels b and c. Independent replicates for a were conducted in 3 pairs of littermate control and *aSMC-cKO*^*Grin1*^ mice, n = 10 arterioles per group. Data are mean ± s.e.m.; nested, unpaired, two-tailed *t*-test **(b, c)**. Source data

Importantly, the disruption of Glu-NsMJ transmission in *aSMC-cKO*^*Grin1*^ mice suppressed the effects of both broad and focal single-axon stimulations on vasodilation (6.7% ± 0.4% versus 15.1% ± 0.7%; Fig. 5l and Extended Data Fig. 8c–e), despite the successful activation of ChR2^+^ perivascular axons (Supplementary Video 7). The latency of vessel dilation following the axonal Ca^2+^ peak remained unchanged between the two genotypes (Fig. 5m). Although the 80-mW laser was 80% successful at inducing axon activations (Fig. 5j), less than 20% of these activated axons could induce p-arteriole dilation (Fig. 5n), which is consistent with the low prevalence of NsMJs (Fig. 1h). In addition, we confirmed that there were no changes between littermate controls and *aSMC-cKO*^*Grin1*^ mice in terms of the basal arteriolar vasomotion, anatomical features of middle cerebral artery vascular trees, contractile protein mRNA expression levels, such as *Acta2*, *Smtn* and *Tagln*, protein expression of smoothelin or smooth muscle cell coverage rate on arterioles (Extended Data Fig. 9 and Supplementary Fig. 15). These results suggest that the absence of GluN1 in aSMCs specifically impairs the amplitude of diameter changes in responding to neural inputs but likely no other features. Thus, these results together demonstrate that specific glutamate transmission in Glu-NsMJs is essential for targeted vasodilation and is more potent than postsynaptic neural signals at least under this specific context.

### Glu-NsMJ mediates sensory input-evoked regional CBF changes

**Fig. 6:**
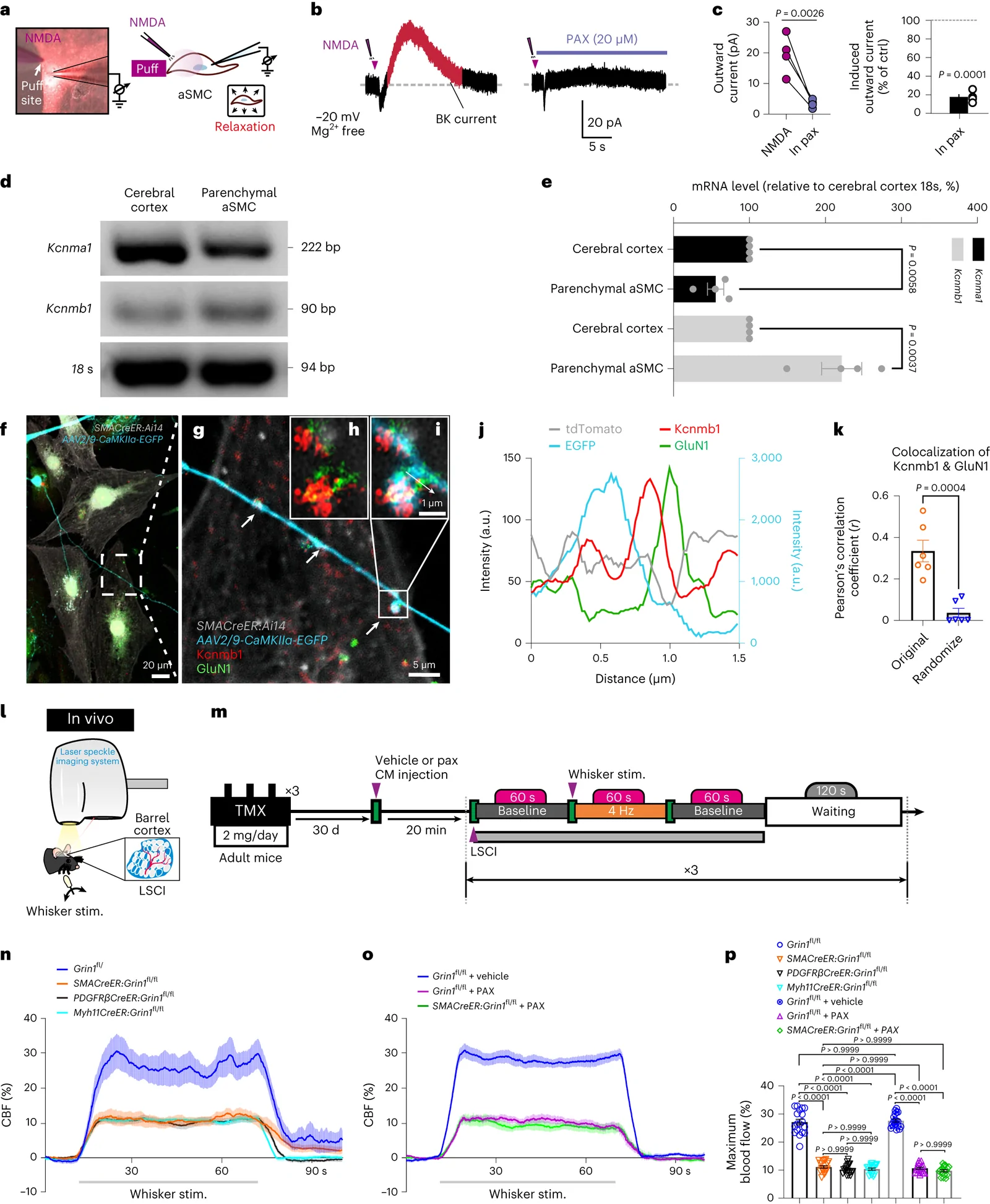
Glu-NsMJ transmission mediates NVC via activating hyperpolarizing BK channels in aSMCs. **a**, Schematic representation displays the spatial positioning of electrodes, filled with NMDA (100 μM) in the left one and configurated with a recording electrode in the right. **b**, Raw traces of the NMDA-induced currents at holding potential of –20 mV under Mg^2+^-free aCSF conditions before (left) and after (right) bath application of PAX (20 μM). **c**, Statistical graphs for **b** (*N* = 4 cells). **d**, RT–PCR of BK channel subunits *Kcnma1* and *Kcnmb1* gene expression levels in cerebral cortex and sorted parenchymal aSMCs. **e**, Statistical graphs show relative mRNA expression levels of *Kcnma1* and *Kcnmb1* in **d** (*N* = 4 mice). **f**, Immunofluorescence staining of Kcnmb1 (red) and GluN1 (green) in the co-culture of pial tdTomato^+^ aSMCs (white) and cortical neurons (cyan) infected by *AAV2/9-CaMKIIα-EGFP* virus. **g**, Zoom-in image of **f**. **h**,**i**, High-resolution confocal images show colocalization of Kcnmb1 with GluN1 in a putative NsMJ in vitro. **j**, Line histograms of Kcnmb1 and GluN1 protein pixel intensity along the white arrow in **i**. **k**, Statistical analysis for the colocalization of Kcnmb1 and GluN1 in **f**–**i**. *N* = 6 independent co-cultures. **l**,**m**, Schematic of LSCI imaging of CBF in mouse barrel cortex during whisker stimulation (**l**) and experimental workflow (**m**). Littermate control (*Grin1*^fl/fl^) and *cKO*^*Grin1*^ (*SMACreER:Grin1*^fl/fl^, or *PDGFRβCreER:Grin1*^fl/fl^ or *Myh11CreER:Grin1*^fl/fl^) mice were intragastrically administered tamoxifen (TMX). **n**–**p**, Time course (**n** and **o**) and maximum percentage (**p**) of CBF changes in the barrel cortex from littermate control and *cKO*^*Grin1*^ mice treated with vehicle or PAX upon whisker stimulations. *N* = 6 mice per group, 3 stimulations for each mouse. The gray horizontal lines show the duration of whisker stimulation. Data are the mean ± s.e.m.; nested, unpaired, two-tailed *t*-test (**c**, **e** and **k**); one-way ANOVA with a post hoc Bonferroni multiple-comparison adjustment (**p**). a.u., arbitrary units. Source data

Large-conductance calcium-activated potassium channels (BK channels) are modulated by both membrane potential and Ca^2+^ sparks near cell membranes, then hyperpolarizing and relaxing aSMCs^46^. We hypothesized that in aSMCs, the activation of NMDA receptors in Glu-NsMJs leads to the influx of calcium ions, which subsequently bind to and activate the BK channels, resulting in an augmented potassium ion efflux, causing membrane hyperpolarization and subsequent relaxation of aSMCs. To test this hypothesis, we held aSMC membrane potential at −20 mV instead of −40 mV, because the BK channel is more prone to opening at −20 mV than at −40 mV^50^. We detected outward currents following NMDA currents, which were nearly completely attenuated in the presence of BK channel blocker paxilline (PAX; Fig. 6a–c). Furthermore, consistent with the previous report^23^, we confirmed that aSMC indeed expressed BK channel α and β subunits *Kcnma1* of *Kcnmb1* mRNAs (Fig. 6d,e; in which the mRNAs were split from the same source as in Supplementary Fig. 7 and Extended Data Fig. 5a,b). In co-culture, we further observed the β subunit protein Kcnmb1 colocalized with GluN1 at the interface of the axon and aSMC (Fig. 6f,k). These results together suggest that, like in neurons^50^, the NMDA receptor-dependent Ca^2+^ spark-like event was one of the calcium sources for BK channels in aSMCs, providing a possible underlying mechanism for how Glu-NsMJ transmission dilated arterioles (Fig. 5).

To determine the importance of Glu-NsMJ transmission in physiological NVC, for instance, functional hyperemia in the barrel cortex of anesthetized mice induced by whisker brushing (Fig. 6l, Supplementary Fig. 16a). We used laser speckle contrast imaging (LSCI) to record blood flow changes in the barrel cortex of adult mice receiving tamoxifen (Fig. 6m). *SMACreER:Grin1*^fl/fl^ and *Myh11CreER:Grin1*^fl/fl^ mice exhibited a dampened blood flow increase compared to that of control mice (Fig. 6n,p). In addition, to assessing the contribution of contractile capillary pericytes, we found *PDGFRβCreER:Grin1*^fl/fl^ induced a similar extent of reduction as *SMACreER:Grin1*^fl/fl^ mice (Fig. 6n,p), suggesting Glu-NsMJ transmission dominantly occurred on aSMCs. While pharmacological inhibition of BK channels via cisterna magna (CM) injection was not clean, it did not achieve an additive CBF change when applied to *SMACreER:Grin1*^fl/fl^ mice (Fig. 6o,p), suggesting BK channels work together with Glu-NsMJ. These results demonstrated that the disruption of Glu-NsMJ substantially inhibited NVC.

To examine the interaction between the nonsynaptic diffusive and synaptic-like transmission pathways, the COX2–PGE2 pathway was chosen because PGE2 is a major diffusive vasodilatory factor in NVC^51–53^. Therefore, we characterized NVC in *SMACreER:Grin1*^fl/fl^ mice that received CM injections of the COX2 inhibitor NS-398 or its vehicle. We found that the application of NS-398 in *SMACreER:Grin1*^fl/fl^ mice synergistically suppressed hemodynamic increases substantially less than individual pharmacological or genetic manipulations (Supplementary Fig. 16b,c), implying that Glu-NsMJ’s role in NVC is independent of the COX2–PGE2 pathway and at least as important as the nonsynaptic diffusion strategy for NVC.

### *Grin1* ablation in aSMCs attenuates pathological arteriolar constrictions

Because pharmacological blockade of NMDA receptors in the contractile aSMCs inhibited both small and large forms of Ca^2+^ activity in response to low and high concentrations of glutamate in vitro (Fig. 4d–h), we hypothesized that glutamate-receptive aSMCs play an essential role in the regulation of the ischemic SD-induced vasoconstriction, opposite to its role in mediating vasodilation with the low level of glutamate release under physiological conditions.

**Fig. 7:**
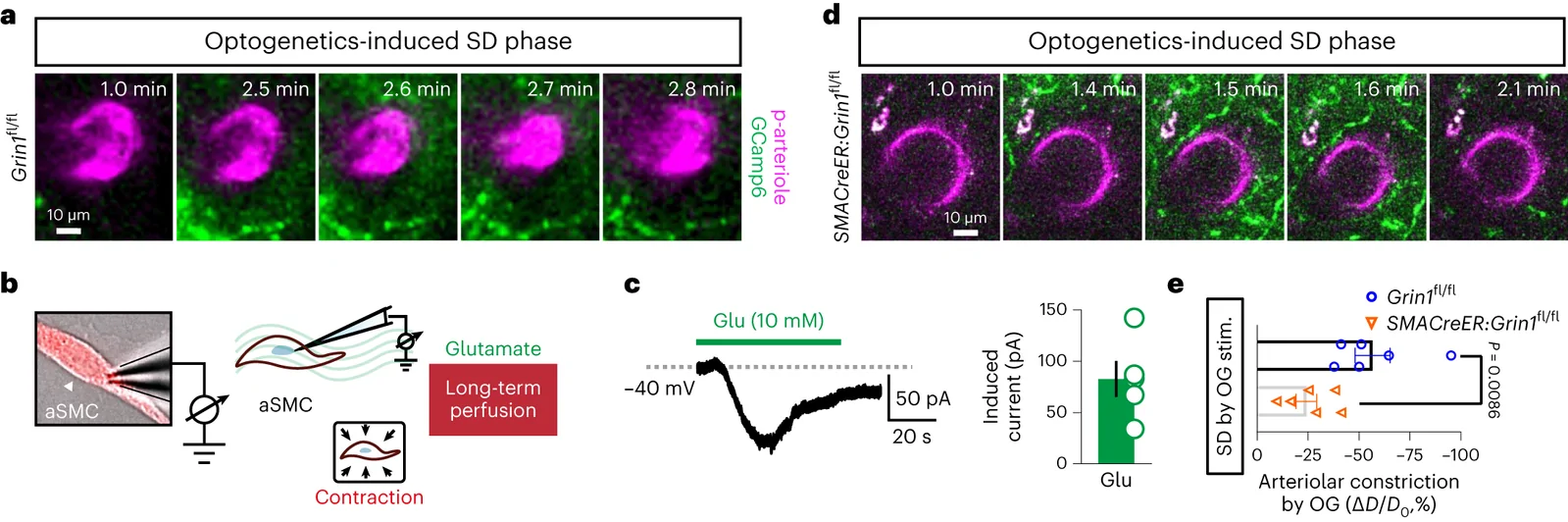
Genetic disruption of GluN1 in aSMCs attenuates vasoconstriction following neuronal SD induced by 2P optogenetics. **a**, Sequential 2P living images of p-arteriolar drastic constriction in control mouse brain (*Grin1*^fl/fl^) during an artificial SD. **b**, Schematic shows a recorded tdTomato^+^ aSMC under long-term perfusion of glutamate. **c**, Raw trace of mega inward current recorded from primary aSMCs, perfused with glutamate (10 mM) for 1 min at a holding potential of –40 mV (left), and summary graphs illustrate the average amplitude of the glutamate-induced inward current (right; *N* = 5 cells). **d**, Sequential 2P living images of p-arteriolar drastic constriction in *aSMC-cKO*^*Grin1*^ mouse brain (*SMACreER:Grin1*^fl/fl^) during an artificial SD. **e**, Statistical analysis of maximum arteriolar constrictions in **a** and **d** (*N* = 6 mice), Artificial SD was induced and imaged on the ipsilateral side of virus injection. Data are the mean ± s.e.m; nested, two-tailed *t*-test (**e**). Source data

Because glutamatergic neuron excitation is the major initiator of SD^54,55^, we first artificially induced glutamate over-release by using prolonged, broad 2P optogenetic stimulation of glutamatergic neurons, which was also able to sufficiently induce SD, which we termed the artificial SD (Fig. 7a). We observed immediate, profound penetrating arteriolar constrictions, even sometimes obliteration of the lumen, following the artificial SD (Fig. 7a). Differently from puffing (~1 s) glutamate and inducing a small inward current, long-term perfusion (1 min) of glutamate to primary leptomeningeal or brain parenchymal aSMCs induced inward mega currents (Fig. 7b,c), suggesting overaction of NMDA receptors. This overactivation may subsequently activate other voltage-dependent Ca^2+^ channels which are abundantly expressed in aSMCs^56^. Then, it likely induces Ca^2+^ waves (Fig. 4e) and leads to aSMC constriction under the artificial SD. This notion was further supported by the finding that *SMACreER:Grin1*^fl/fl^ mice exhibited less severe arteriolar constriction when receiving similar artificial SD (Fig. 7d,e). These results demonstrate that moderate and extreme levels of activation of glutamatergic neurons result in opposite effects on vasomotor activity (Figs. 5i and 7e).

### *Grin1* removal in aSMCs improves stroke recovery

**Fig. 8:**
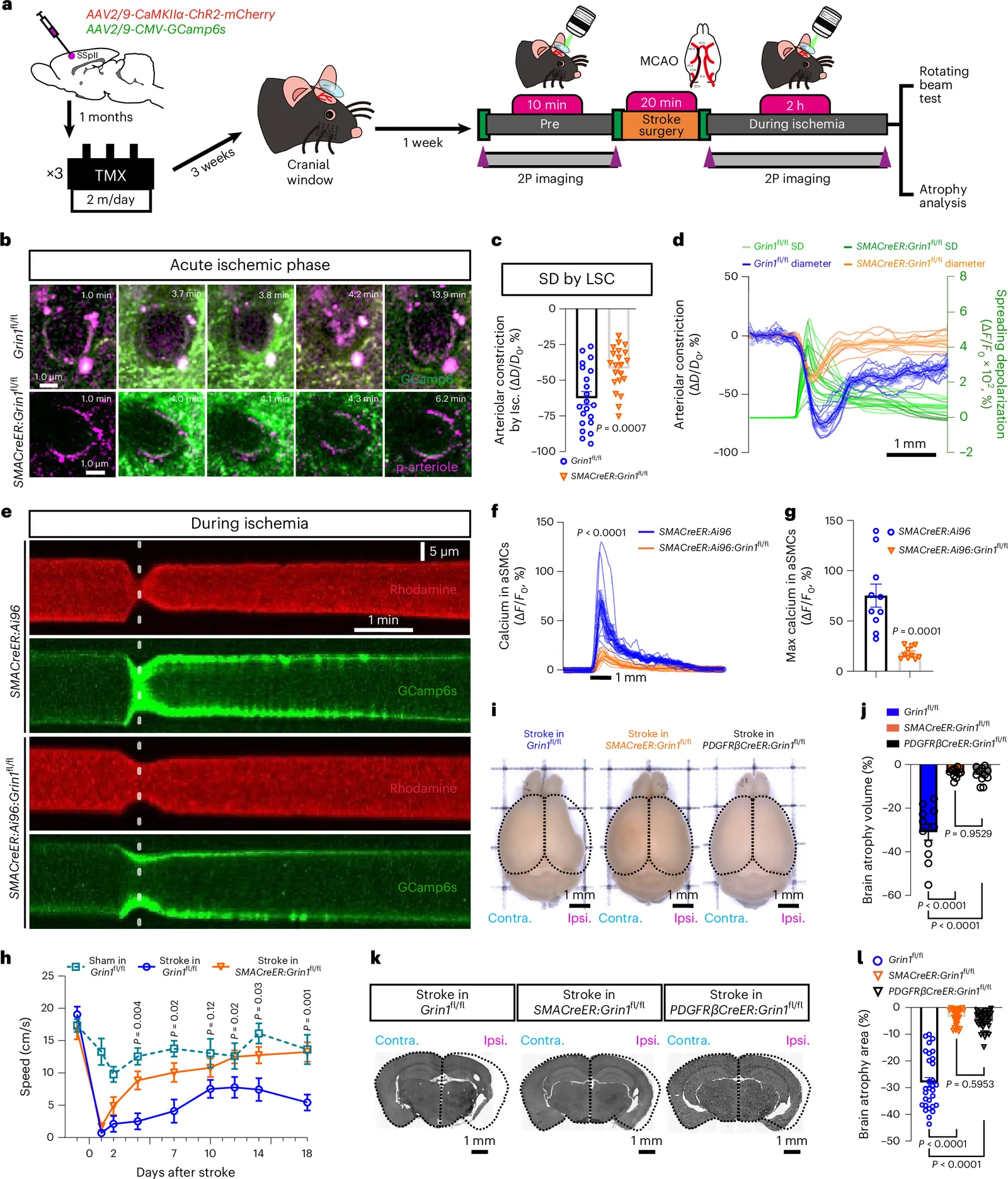
Genetic perturbation of GluN1 in aSMCs boosts functional recovery after ischemic stroke. **a**, Schematic for 2P observation of ischemic SD and vasoconstrictions in control and *aSMC-cKO*^*Grin1*^ mice. **b**, Sequential 2P living images of p-arteriolar drastic constriction in control and *aSMC-cKO*^*Grin1*^ mouse brains during ischemic SD. Arterioles labeled by hydrazide 633 (magenta) and SD indicated by Ca^2+^ propagation (GCamp6, green). **c**, Maximum changes in arteriolar diameter in drastic constriction as shown in **b** (*N* = 10 mice, *n* = 21 arterioles/genotype). Isc., ischemia. **d**, Time course of neural GCamp6 signal during SD and corresponding arteriolar constriction *Grin1*^f/f^ mice: *N* = 6 mice, *n* = 10 arterioles and neural SD-component sets; SMACreER:*Grin1*^f/f^ mice: *N* = 6 mice, *n* = 9 arterioles and neural SD-component sets. **e**, Kymography of arteriolar blood flow (red; labeled by rhodamine) and corresponding Ca^2+^ dynamics in aSMCs (green; detected by GCamp6) during the acute ischemic phase in control (*SMACreER:Ai96*) and *aSMC-cKO*^*Grin1*^ (*SMACreER:Ai96:Grin1*^fl/fl^) mice. White dotted line indicates time points when arteriolar caliber became narrowest during SD. **f**,**g**, Time course (**f**) and maximum amplitude (**g**) of aSMC Ca^2+^ in **e** (*N* = 3 mice, *n* = 10 arterioles). **h**, Travelling speed of mice in RBT before and at post-stroke days 1, 2, 4, 7, 10, 14 and 18 in sham mice (cyan; *N* = 8 mice), stroke in *Grin1*^fl/fl^ (blue; *N* = 9 mice) and stroke in *aSMC-cKO*^*Grin1*^ (orange; *N* = 9 mice) groups. Sham mice and *Grin1*^fl/fl^ mice were subjected to the same surgical operations to induce middle cerebral artery occlusion (MCAO) except without inserting a suture into the common carotid artery. **i**–**l**, Brain atrophy volume (**i**) and section (**k**) at post-stroke day 18 in control (*Grin1*^fl/fl^) and *cKO*^*Grin1*^ (*SMACreER:Grin1*^fl/fl^ or *PDGFRβCreER:Grin1*^fl/fl^) groups, with quantification in **j** (*N* = 11 mice) and **l** (*N* = 30 sections from 6/11 mice in **j**), respectively. Brain atrophy area (%) = (total Ipsi area – total Contra area)/total Contra area × 100%. Contra, contralateral; Ipsi, ipsilateral. Data are the mean ± s.e.m; nested, two-tailed *t*-test (**c** and **f**–**h**), one-way ANOVA after post hoc Bonferroni multiple-comparison adjustment (**j** and **l**). All mice in the control group used for experiments were littermates and all animals were anesthetized during 2P stimulation and imaging. Source data

Next, we combined real-time vascular imaging with middle cerebral arterial occlusion to induce stroke to directly test our hypothesis of whether p-arteriolar constriction following the ischemic SD is prevented in *aSMC-cKO*^*Grin1*^ mice (Fig. 8a). Notably, the degree of p-arteriolar constriction was substantially attenuated in *aSMC-cKO*^*Grin1*^ mice, despite the presence of comparable SD between the two genotypes (high glutamate; Fig. 8b–d and Supplementary Video 8). This finding demonstrates that glutamate-receptive aSMCs are essential for the ischemic SD-induced pathological vasoconstriction.

It is well known that cytosolic Ca^2+^ level is tightly correlated with aSMC contractility^47^. To test whether *Grin1* knockout protects aSMCs from the glutamate excitotoxicity-induced Ca^2+^ overloading and subsequent vasoconstriction, we generated the triple genetically edited line *SMACreER:Ai96:Grin1*^fl/fl^, in which aSMC cytosolic Ca^2+^ changes can be determined. Using the same imaging paradigm, we found that during the acute 2-h occlusion period, remarkable Ca^2+^ elevations in aSMCs repeatedly occurred in concomitance with drastic vasoconstrictions (Fig. 8e,f and Supplementary Video 9). Notably, this abnormal Ca^2+^ elevation was substantially suppressed in *aSMC-cKO*^*Grin1*^ mice (Fig. 8e,g and Supplementary Video 9). Together, these findings demonstrated that the NMDA receptor in aSMCs is a sensory factor that allows arterioles to bidirectionally adjust their vascular responses by detecting the microenvironmental glutamate concentrations at the physiological or pathological level.

**Extended Data Fig. 10:**
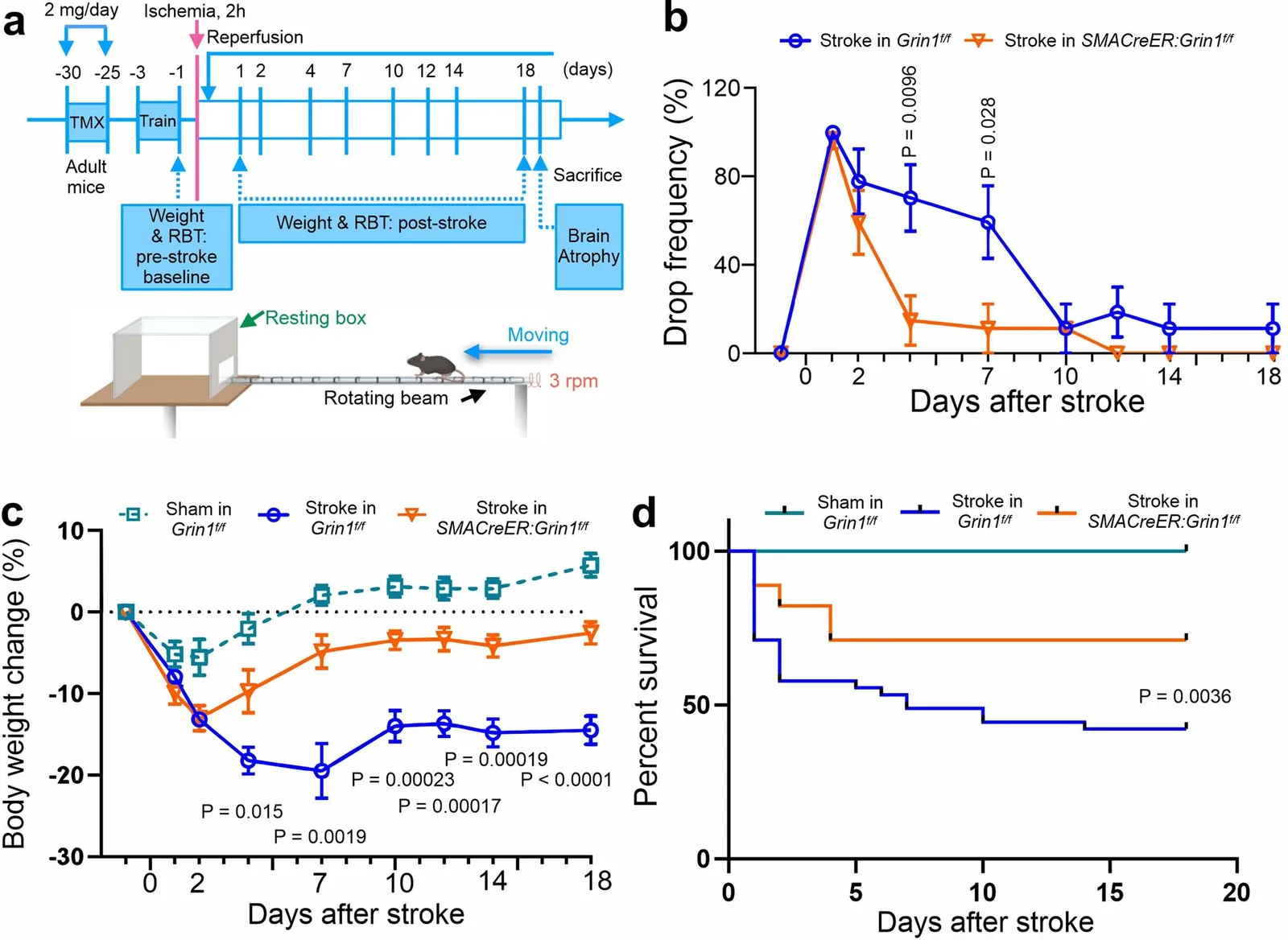
Conditional knockout of GluN1 from aSMCs promotes functional recovery after ischemic stroke. **(a)** Upper panel: experimental designs for recovery evaluation of mice subjected to cerebral ischemia. Lower panel: a sketch of the paradigm of RBT, created with BioRender.com. Tamoxifen was given to adult mice. Falls off frequency of the RBT **(b)**, body weight changes **(c)**, and survivorship curve **(d)** during post-stroke 18 days in sham (*Grin1*^*f/f*^, N = 8 mice), stroke in control (*Grin1*^*f/f*^, N = 9 mice), and stroke in *aSMC-cKO*^*Grin1*^ group (*SMACreER:Grin1*^*f/f*^, N = 9 mice). Data indicates the mean ± s.e.m.; nested, two-tailed *t*-test **(b)**, one-way ANOVA after a post hoc Bonferroni multiple comparison adjustment **(c)**. Log-rank (Mantel-Cox) and Gehan-Breslow-Wilcoxon for d. All mice applied for the experiments were males. Source data

Next, we decided to continue examining mouse motor behavior and brain tissue atrophy according to the experimental design in Extended Data Fig. 10a. We assessed mouse motor ability and balance control using the rotating beam test (RBT)^57^. Notably, we found that the speed with which *aSMC-cKO*^*Grin1*^ mice crossed a rotating beam increased substantially sooner than that of littermates after ischemic stroke (Fig. 8h). Compared with control littermates, *aSMC-cKO*^*Grin1*^ mice showed a substantially reduced number of falls off the rotating beam after ischemia (Extended Data Fig. 10b). Even more strikingly, *aSMC-cKO*^*Grin1*^ and *PDGFRβCreER:Grin1*^fl/fl^ mice exhibited only slight cerebral atrophy at 18 d after reperfusion, whereas their control littermates showed substantial tissue loss (Fig. 8i,j). Brain sections revealed that tissue loss in the striatum and cortex was dramatically prevented in *aSMC-cKO*^*Grin1*^ and *PDGFRβCreER:Grin1*^fl/fl^ mice (Fig. 8k,l). Moreover, the decrease in body weight was more mild (Extended Data Fig. 10c), and the survival rate was substantially higher in *aSMC-cKO*^*Grin1*^ mice than in control littermates after stroke (Extended Data Fig. 10d). Taken together, these results demonstrate insult to arterioles from excitotoxicity during ischemia, suggesting that prevention of NMDA receptor-dependent arteriolar constriction is beneficial for brain and behavior recovery after stroke.

## Discussion

Brain parenchymal vessels are innervated by a variety of types of perivascular nerve endings^14^. Cerebral aSMCs express many types of neurotransmitter receptors^23,24^ at various levels, with some expressed at extremely low levels (Extended Data Fig. 5b), yet whether synaptic-like transmission is truly engaged in neurovascular communications and how important it is remains elusive. In the present study, using 3View ultrastructural volume reconstruction, line-optogenetic and field-optogenetic stimuli while simultaneously measuring neural activity and vascular dynamics under 2P microscopy, physiological and pathological stimuli combined cell-type-specific genetic perturbations, we establish a clear-cut, definitive NsMJ transmission, which has a key role in delivering signals from the nervous side to the vascular side in NVC. Furthermore, we demonstrate Glu-NsMJ activation leads to not only vasodilation in physiological conditions but also vasoconstriction in pathological conditions, depending on glutamate levels. Notably, selective perturbation of NMDA receptors in aSMC can attenuate severe vasoconstriction during acute stroke and hence prevent brains from atrophy, suggesting a novel therapeutic strategy by manipulating neurovascular cross-talk.

We propose the two messenger delivery strategies—nonsynaptic diffusion and synaptic-like transmission—are both engaged in delivering vasoactive factors to aSMCs. Unlike the diffusion strategy, NsMJs can perform dual communications with postsynaptic neurons and postjunctional aSMCs using the same neurotransmitter molecule without taking extra steps to synthesize additional messengers. In contrast, the diffusion strategy requires extra steps to synthesize and release vasoactive factors, such as PGE2 synthesized by COX2 in pyramidal neurons, EETs by cytochrome P450 epoxygenases in astrocytes, NO by NO synthase in interneurons or ECs when their NMDA receptors or mGluRs are activated^1^. These findings not only suggest that synaptic-like transmission potentially delivers neural signals to aSMCs faster than the diffusion strategy but also provide the possibility of distal gating of CBF, as neural fibers can project to arterioles in distal regions, which is hardly achieved by the local diffusion mechanism.

We confirmed that the previously reported COX2–PGE2 pathway also has a role in NVC. However, we found that the NsMJ strategy works independently of the COX2–PGE2 pathway (Supplementary Fig. 16b,c) and makes up to 60% of the contribution of the CBF increase induced by sensory inputs. This large contribution to NVC by direct innervation of arterioles implies that the brain evolved a self-regulation strategy, using a proactive controlling mechanism to powerfully influence blood flow even in areas where there is no neural activity within the surrounding tissue^10^. The selectivity and the heterogeneity of neighboring p-arterioles to sensory stimuli^4,10^ may be linked to the NsMJ strategy.

The constrictive and dilatory neuromediators^6,13,14,16^ released by different types of neurons could determine the heterogeneity of NsMJ transmission, which can likely explain the following elusive issues in the NVC field. First, how the increase in CBF can be sustained in the presence of a reduction of the local neural network caused by optogenetic activation of inhibitory GABAergic neurons^11^. As traditional two-dimensional EM revealed some GABAergic terminals directly apposed to pericytes^58^, we speculate that GABAergic terminals possibly form GABA-NsMJs with dual innervations, where the parental bouton inhibits the neuronal network activity while the daughter bouton simultaneously dilates vessels. Indeed, we detected several GABA_A_ receptor subunits (*Gabra2*, *Gabra3*, *Gabrg2* and *Gabrd*) in bulk RNA-seq results (Extended Data Fig. 5b), which constitute chloride-permeable channels. Negatively charged chloride ion influx possibly produces aSMC membrane hyperpolarization, which has been linked to aSMC relaxation and vasodilation^46^.

Second, the finding that differently located arterioles respond heterogeneously upon receiving a limb stimulus^4^ may be alternatively explained by heterogeneous dilatory or constrictive NsMJs that differentially innervate those arterioles. These opposite NsMJs would compete as antagonistic forces to control arteriolar diameter, with their strengths proportional to the efficiency of NsMJ transmissions. Third, potential constrictive NsMJ transmissions may provide the underpinning mechanisms for task-related negative blood oxygen level-dependent signals in functional magnetic resonance imaging. For example, NPY-positive interneurons are implicated in driving negative blood oxygen level-dependent signals, which is possible through constrictive NPY-NsMJs onto NPY-Y1 receptor-positive aSMCs^59^ (Extended Data Fig. 5b). The heterogeneity of NsMJ transmission could provide the structural and molecular foundations for the theory of cell-type-specificity-dependent CBF regulation in the brain, which may produce heterogeneity in NVC presentations. In future work, a topological whole-brain mapping neurovascular connectome based on NsMJ merits further efforts.

In addition, we confirmed ECs express NMDA receptors not only in mice but also in monkeys and humans using immunoelectron microscopy (Fig. 3l–p), suggesting that glutamate can act on two independent vascular cell types to mediate NVC. NMDA receptors cluster in aSMCs and appose axonal terminals (Fig. 3l), while in ECs, they are predominantly distributed on the basolateral side^38^. The distinct subcellular localization of NMDA receptors in aSMCs compared to ECs indicates their different spatial relationship with axons and astrocytic endfeet. It is possible that ECs primarily react to glutamate released by astrocytes, whereas aSMCs primarily respond to glutamate released by neurons. How EC and aSMC NMDA receptors differentially contribute to NVC requires further investigation.

The newly developed ultrastructure detection technology has high throughput and resolution, facilitating quantitative characterization of the neurovascular unit of cerebral vasculature in three dimensions. Traditional two-dimensional EM requires considerable time to examine neurovascular units of three p-arterioles at a length of 450 μm; however, SBF-SEM can image these units. The 3D reconstructions in this study revealed larger exposure areas in p-arterioles than is usually thought for the capillaries^17^. Our findings indicate each aSMC has 2–3 NsMJs on average. This result was based on the finding of 499 NsMJs after examining close to 200 aSMCs from three p-arterioles. By extrapolation, there might be 8 million NsMJs distributed throughout the mouse brain, and only a small number of neurons establish NsMJ connections with aSMCs. If each NsMJ requires one neuron to form, at most 10% of the total neurons in the mouse brain would directly innervate aSMCs. Therefore, it might be reasonable that the success rate of arteriole dilation following successful single-axon activation was less than 20% (Fig. 5n).

Human airway SMCs have been demonstrated to express GluN1^40^. This study extends the concept to ‘vascular’ SMCs, which appear to be restricted to the brain but not peripheral organs (Extended Data Fig. 6). This suggests that cerebral aSMCs are a specialized type among whole-body vascular SMCs. Our research also demonstrates that GluN1 expression in cerebral aSMCs is conserved in higher primates, suggesting that direct Glu-NsMJ transmission occurs in human brains.

aSMCs are a cell type in the brain that show rapid contraction and relaxation^8^, which only account for about 0.3% of the total population (Extended Data Fig. 2j,k). It is an intriguing finding that manipulation of such a small proportion of brain cells can promote brain function recovery after stroke. Since the central function of aSMCs is to regulate CBF, our research indicates that finely tuning CBF during the reperfusion period may be critical for ensuring sufficient oxygen and glucose delivery to the affected brain regions. Although prior studies have shown that NMDA receptor blocker MK801 failed to reduce the frequency of SD, it did diminish the severity of episodic hypoperfusions^60^. Our findings of reduced arteriolar constriction in *aSMC-cKO*^*Grin1*^ mice (Fig. 8) may explain the beneficial effect of MK801. In addition, the interplay between calcium ions, NMDA receptors and BK channels plays a crucial role in the modulation of aSMC function and could potentially serve as a therapeutic target for stroke and related cerebrovascular disorders by avoiding the spreading of ischemic damage.

## Methods

### Animal and animal care

All mice procedures complied with the Institutional Animal Care and Use Committee guidelines of the School of Life Sciences, Westlake University (approval no. 20-033-JJM). *NG2DsRedBac*, *Thy1-YFP-H*, *Ai14* (Gt(ROSA)26Sor^tm14(CAG-tdTomato)Hze^) and *Ai96* (Gt(ROSA)26Sor^tm96(CAG-GCaMP6s)Hze^ mice (The Jackson Laboratory, stock nos. 008241, 003782, 007914 and 024106, respectively) were kindly provided by the laboratory of W.-p.G. at the Chinese Institute of Brain Research (CIBR). The laboratory of B.Z. kindly shared *SMACreER* (Tg(Sma-CreERT2)12Pcn) mice at the Shanghai Institutes for Biological Sciences, Chinese Academy of Sciences. *Ai47* (Gt(ROSA)26Sor^tm47(CAG-EGFP)Hze^) mice were kindly offered by the laboratory of Z.Q. at Chinese Academy of Sciences Center for Excellence in Brain Science and Intelligence Technology, Chinese Academy of Sciences. *Grin1*^fl/fl^ (Grin1^tm2Stl^, The Jackson Laboratory, stock no: 005246) mice were kindly supplied by the laboratory of Y.S. at Chinese Academy of Sciences Center for Excellence in Brain Science and Intelligence Technology, Chinese Academy of Sciences. *PDGFRβCreER* (Tg(Pdgfrb-cre/ERT2)6096Rha, The Jackson Laboratory, stock no: 029684) mice were kindly shared by the laboratory of X.Y. at Peking University*. Myh11CreER* (Tg(Myh11-cre/ERT2)F31Gko/J, The Jackson Laboratory, (stock no. 037658) mice were kindly provided by C.L. at Sun Yat-sen University. Wild-type C57BL6/J mice were purchased from the Laboratory Animal Resources Center of Westlake University. Standard chow and water were provided to the mice ad libitum. Four mice were housed in each cage in a standard animal room on a 12-h light/dark cycle (lights on at 07:00) at 25 °C with 40–60% humidity.

### Tamoxifen administration regime

In general, for all in vitro primary culture experiments involving primary SMCs, tamoxifen was added to the cell culture medium 1 week before subsequent experiments, except in Extended Data Fig. 2i–q, where tamoxifen was administered at embryonic day 18 for three continuous days. For all in vivo experiments, tamoxifen was intragastrically administered in adults at least 1 month before the following experiments, including bulk RNA-seq experiments. In addition, all littermate control mice throughout the study received tamoxifen strictly at the same doses as the conditional knockout groups.

### Brain tissue acquisition from high primates

The monkey brain was obtained from a male 14-year-old rhesus macaque with informed consent under a protocol approved by the Institutional Animal Care and Use Committee guidelines of the School of Life Sciences, Westlake University. The brain was shared by Hainan Jingang Biotech in Hainan, China. The brain was immediately immersed in 4% paraformaldehyde (PFA) following the dissection. The postmortem Brodmann brain area tissue was obtained from a generous adult donor through the Shanghai Red Cross. All tissue samples were donated with full, informed consent. The donor, a 91-year-old female with a medical history of heart disease and hypertension, gifted the tissue to further scientific research. The procurement was conducted at Huashan Hospital in accordance with a protocol approved by Huashan Hospital of Fudan University and with the explicit consent of local ethics committees. With an approved material transfer agreement, the collected tissue was stored in liquid nitrogen during transportation from the hospital and the laboratory. Hereafter, all procedures were carried out on ice in a 4 °C cold room as rapidly as possible. All experiments on the brains of humans and monkeys were performed according to institutional and national guidelines.

### Tissue clearing and light-sheet imaging

We performed whole-brain tissue clearing according to the CUBIC method with minor modifications^61^. Briefly, adult mice were deeply anesthetized with sodium pentobarbital at a dose of 100 mg per kg body weight by intraperitoneal injection and then transcardially perfused with fixative buffer containing 4% PFA and 4% sucrose (pH 7.4) in PBS. Mouse brains were dissected, postfixed in fixative buffer at 4 °C for 24 h, and then washed in 10 ml of PBS containing 0.01% NaN_3_ (wt/vol) for at least 2 h at room temperature. The samples were immersed in 8–10 ml of 0.5× CUBIC reagent 1 (25% urea, 25% quadrol, 15% Triton X-100 in double distilled water (ddH_2_O)) and shaken for 3–6 h. Subsequently, the samples were immersed in 1× CUBIC reagent 1 for 7–8 d under gentle shaking at ~50 r.p.m. After rinsing with PBS, the brain samples were cleared in ~5 ml of 0.5× CUBIC reagent 2 (50% sucrose, 25% urea, 10% triethanolamine, 0.1% Triton X-100 in ddH_2_O) for 6–24 h with gentle shaking and then in 1× CUBIC reagent 2 for an additional 6–7 d. CUBIC reagents 1 and 2 were refreshed every 2 d until the brains were completely cleared. All incubation steps in either CUBIC reagent were at 37 °C. The cleared brains were imaged with a light-sheet microscope (Carl Zeiss Z.1) acquired by Zeiss ZEN software (version 3.6) using a ×5 (NA = 0.16) and ×20 (NA = 1.0, ND = 1.45) objective.

### Brain sample preparation for in vivo CLEM

A 3-mm cranial window overlaying the somatosensory cortex was created in 4-month-old *NG2DsRedBac:Thy1-YFP* double transgenic mice (Supplementary Fig. 4a–c). P-arterioles associated with the middle cerebral artery were imaged by a 2P microscope (Olympus FLUOVIEW, FVMPE-RS; laser, InSight X3) at a resolution of 0.994 μm per pixel with a ×25/1.05w MP XL PlanN objective (Supplementary Fig. 4d–h). To mark the margins for the imaged ROIs, the widely tunable ultrafast laser in constant angular velocity tornado mode with 100% power at 960 nm was applied.

Anesthetized mice were transcardially perfused with a total volume of 200 ml of fixative buffer containing 2% PFA, 1.25% glutaraldehyde and 4% sucrose in 0.08 M cacodylate buffer. Then, a 0.9 × 1.2 × 1.2 mm^3^ tissue block after the live imaging acquisition was precisely dissected according to the marks made by laser burning using tornado mode (Supplementary Fig. 4i–l) and kept in perfusion buffer for an additional 24 h until osmium impregnation (OTO). This tissue block was washed with cacodylate buffer (0.15 M, pH 7.4) three times, postfixed with an ice-cold 2% osmium tetroxide solution (Ted Pella) for 1 h, and then directly placed in KFeCN (2.5%) buffer for another 1.5 h at 4 °C. After three rinses with cacodylate buffer, this tissue block was placed in a freshly prepared 1% thiocarbohydrazide solution in a water bath at 58 °C for 30 min, washed with ddH_2_O, and incubated for a second time in ice-cold osmium postfixation buffer for 1.5 h. After washing with ddH_2_O, the tissue block was incubated in 1% uranyl acetate for 2 h at 50 °C. It was rinsed in ddH_2_O, immersed in a lead aspartate solution in a stirred water bath at 50 °C for another 2 h, and dehydrated in increasing ethanol concentrations (50%, 70%, 80%, 90% and 100% in ddH_2_O, twice respectively). Finally, the tissue block was evaporated with acetone and embedded in resin (Epon 812, Ted Pella) and subjected to X-ray imaging and 3View imaging (Supplementary Fig. 4m,n).

### SBF-SEM imaging

Using X-ray microscopy (XRM, Xradia 520 Versa, Carl Zeiss) at a magnification of ×4 and 80 kV, the three target p-arterioles of the middle cerebral artery (Extended Data Fig. 4a) were identified in this 0.9 × 1.2 × 1.2 μm^3^ tissue block at a resolution of 1.8 μm per pixel. According to XRM imaging and the four-laser-burned spot markers, the tissue block was further precisely trimmed using Gillette blue blades under a stereoscopic microscope and adhered to an aluminum metal pin (Extended Data Fig. 1c) using conductive epoxy (CW2400, Chemtronics). The specimen was further precisely trimmed into a block of 600 × 600 × 700 μm^3^ using a glass knife and a right-angled diamond knife (Trim 90, DiATOME). After coating with gold-palladium (EM ACE200, Leica) and a final trim using a 3View ultramicrotome (Gatan), serial image acquisition was performed by using a BSE detector on a Carl Zeiss Gemini 300 scanning electron microscope at 2 kV voltage and approximately 1.8 × 10^−3^ mBar in high-vacuum mode with focus charge compensation. The specimens were sectioned at a thickness of 50–75 nm and a cutting speed of 0.1 mm s^−1^. Serial images for the 3D stack were reconstructed with an adjustment of 5–6 nm per pixel and a 0.7–1 μs dwell time.

### 3D reconstruction and analysis

First, using Amira software (Thermo Fisher), serial stack SEM images were auto-aligned to register each slice. The mismatched regions were cropped and filtered with a nonlocal meaning and membrane enhancement algorithm. Astrocytic endfeet, blood vessels and neuronal axons and dendrites were manually segmented using Imaris software (Oxford, Bitplane), proofread and semiautomatically segmented using the segmentation module of Amira. Both Amira and Imaris software were exploited to surface render and reconstruct the segmented or extracted cell structures in pseudocolors. Dragonfly software (ORS) was used to superimpose the serial 3View SEM image data with the fluorescence image data acquired with the 2P microscope and matched to the XRM data for the p-arteriole (Extended Data Fig. 4). Quantification modules, including measurement of the rendering volume, meshing surface area and skeleton length, were obtained with either Imaris or Amira. For endfoot reconstruction, the stack images were resampled to a resolution of 10 nm per pixel. Then the endfoot coverage was calculated with the surface–surface contact area algorithm of Imaris by using the following equation: surface coverage = *S*_contact surface area_/*S*_primary surface area_, where *S*_contact surface area_ and *S*_primary surface area_ refer to the surface area of the blood vessel contacted by the endfoot and the total surface area of the blood vessel, respectively. Quantification of vesicle size and NsMJ distribution was based on semiautomated and manual statistical assessment.

### Primary aSMC culture and co-culture with neurons

The mixed cell culture containing aSMCs was prepared by using two tissue sources of neonatal mouse, cerebral leptomeninges or parenchyma. In *SMACreER:Ai14 or SMACreER:Ai47* mice, the reporter proteins (red fluorescent protein tdTomato or the green fluorescent protein EGFP) were specifically expressed in aSMCs by crossing *tdTomato*^fl/fl^ (Ai14) or *tri-EGFP*^fl/fl^ (Ai47) reporter mice with a tamoxifen-inducible *SMACreER* recombinase driver line. As described previously^62,63^, with a few modifications, leptomeningeal and parenchymal aSMCs were separately prepared from P0 mice. Briefly, pup brains were dissected and separated into two components: the leptomeninges without dura (dura was discarded together with skulls) and brain parenchyma. These two components were then separately digested by trypsin and seeded on culture plates filled with DMEM plus 10% FBS or with SMCM medium, both of which are suitable for aSMC culture. The tamoxifen (5 μM; Sigma-Aldrich, T5648) was added to the culture medium to induce reporter protein expression in primary aSMCs.

These mixed primary cells (reporter protein-positive and negative cells) from both tissue sources were either cultured alone or used for neuron–aSMC co-culture (Supplementary Table 2). At 7 d after the seeding, freshly prepared cortical neurons were added; meanwhile, the old medium was replaced with neurobasal A medium to generate a co-culture system. Primarily cultured cells were kept in a 5% CO_2_ humidified incubator at 37 °C for 7–14 d in vitro before the experiments.

Additionally, tdTomato^+^ or EGFP^+^ aSMCs in the mixed primary cells described above were sorted by using FACS, and the sorted aSMC population was expanded by further culture for RT–PCR, western blot and co-culture with neurons for TEM-CLEM experiments (Supplementary Table 2).

### Fluorescence-activated cell sorting of parenchymal aSMCs

Parenchymal aSMCs were dissociated and sorted from neonatal mouse (P1–3) brains for in vitro culture experiments. Briefly, pregnant *SMACreER:Ai14* mice were intraperitoneally injected or intragastrically administered tamoxifen on an embryonic day 18 for three continuous days. Brain samples were collected on P1–3, and their leptomeninges were removed. Single-cell suspensions were obtained using the Neural Tissue Dissociation Kit (P) (Miltenyi Biotec, 130-092-628). Cells were sorted with a BD FACSAria III flow cytometer (BD Biosciences). A 100-μm nozzle and a PBS sheet fluid pressure of 20 psi was used (Extended Data Fig. 2i–k). First, cells were selected with a very wide gating setting using forward scatter area/side scatter area (FSC-A/SSC-A). Second, based on FSC-A/FSC-H (forward scatter high) and further SSC-A/SSC-H (side scatter high), adherent cells were removed twice from the parental FSC-A/SSC-A gate. Finally, fluorescence events were selected from the nonadherent cells. tdTomato was excited with a 561-nm laser, and its emission was detected with a 582/15-nm filter. Wild-type C57BL6/J and *Ai14* singlet-positive mice lacking fluorescence were used as the negative controls. tdTomato^+^ aSMCs were sorted directly into DMEM and seeded on plates for the following primary culture experiments and co-culture experiments (Supplementary Table 2). All FACS data were analyzed using FlowJo (v10.6.2).

For aSMC sorting for the study of profiling molecular expressions, parenchymal aSMCs were obtained from the brains of adult *SMACreER:Ai47* mice, which received tamoxifen 1 month before cell sorting. Parenchymal aSMCs were sorted and applied to bulk RNA-seq and some RT–PCR experiments. All procedures were the same as described above for neonatal aSMC sorting, except that cerebral vessels with minimal inclusion of capillaries were first enriched as described before^64^ proceeding to achieve single-cell suspensions, and negative selections were additionally performed to exclude CD31^+^ ECs and CD45^+^ immune cell contaminations using the PE-Alexa Fluor 594-conjugated anti-CD31 antibody (BioLegend, 102520, clone MEC13.3; 1:200 dilution) and APC-conjugated anti-CD45 antibody (BioLegend, 103112, clone 30-F11; 1:200 dilution).

### CLEM in vitro

SEM-CLEM was conducted using co-cultured neurons and pial aSMCs. The co-cultured cells were immunostained with anti-α-SMA and anti-Tau antibodies, and time-lapse imaging with a Zeiss confocal microscope was performed later. Afterward, they were further postfixed in 2.5% glutaraldehyde at room temperature for 2 h and then kept at 4 °C overnight. The samples were postfixed in an ice-cold 1% osmium tetroxide solution (Ted Pella) for 1.5 h and dehydrated through a graded ethyl alcohol series (30%, 50%, 70%, 80%, 90% and 100% in ddH_2_O twice, respectively). The samples maintained in absolute ethanol were transferred into a carbon dioxide critical point dryer (EM CPD300, Leica) until thoroughly dried. The dried samples were mounted on aluminum stubs and sputter-coated with a gold-palladium layer. Finally, SEM images of the co-cultured cells were acquired with a Gemini300 microscope (Carl Zeiss) at 5 kV.

TEM-CLEM was used to analyze NsMJs in vitro in a co-culture of neurons with sorted parenchymal aSMCs. Bright-field and fluorescence images were acquired before the co-cultured cells were prepared for TEM. Briefly, the cells were fixed with 2% glutaraldehyde in 0.1 M sodium cacodylate buffer for 30 min and then washed in ice-cold 0.1 M sodium cacodylate buffer three times. Next, the cells were postfixed in reduced 1% osmium tetroxide in cacodylate buffer for 1 h and rinsed with 0.1 M sodium cacodylate buffer three times. After washing with ddH_2_O three times, the cells were stained with 1% uranyl acetate in ddH_2_O for 1 h. Next, the cells were rinsed with ddH_2_O, dehydrated with a graded ethanol series, and infiltrated with Durcupan ACM resin. Finally, ultrathin sections were prepared, and TEM images were acquired with a transmission electron microscope (Thermo Scientific, Talos L120C G2) at 80 kV. Markers were used to identify the same cells that underwent fluorescence and TEM imaging in Ibidi μ-dishes (Grid-500).

### CTB for retrograde tracing

HB-vSMCs were preincubated with Alexa Fluor 488-conjugated CTB (1 mg ml^−1^, Invitrogen, C34775) for 2 d, followed by washing three times and maintained in the fresh SMCM medium for another 2 d. Before adding the freshly dissociated cortical neurons, the SMCM medium was replaced by neural basal medium. Time-lapse imaging was performed 7 d after seeding neurons. The retrograde tracing states were recorded using a motorized fluorescence microscope (Olympus, IX83). A control experiment was performed to exclude the possibility of CTB diffusing into the supernatant neural basal medium and then binding to co-cultured neurons. The supernatant medium of the pre-stained HB-vSMCs was collected and added to primary neurons. Notably, under such experimental settings, we did not observe any CTB^+^ neurons.

### Bulk RNA-seq analysis

Messenger RNAs used for bulk RNA-seq were harvested from the sorted parenchymal EGFP^+^ aSMCs from adult *SMACreER:Ai47* mice, which received tamoxifen as adults. RNA was isolated using the RNeasy Plus Mini Kit (Qiagen). RNA-seq libraries were prepared using a TruSeq RNA Library Prep kit v2 (Illumina). The libraries were sequenced on a HiSeq 2500 instrument with single-end 300–400-bp reads (single indexing reads). FastQC and TopHat align were exploited for raw data processing, described previously^65^. The normalized gene count matrix for all genes was used as the input through the htseq-count script. A heat map was generated using R code to visualize the gene expression levels in different groups. The genes of interest were clustered as neuromediator receptors and ranked according to expression level from high to low. The DESeq2 was used to compare differences in gene expression between aSMCs versus cortical tissue^66^.

### RT–PCR

We developed a selective aSMC loss-of-function EGFP-labeling model by generating a triple transgenic mouse line, crossing floxed-Grin1 (*Grin1*^fl/fl^) mice with driver mice expressing Cre-recombinase driven by the SMA promoter (*SMACreER*) and reporter mice expressing *EGFP*^fl/fl^ (*Ai47*). The triplet mutant *SMACreER:Ai47:Grin1*^fl/fl^ mice are conditional knockout GluN1 (*aSMC-cKO*^*Grin1*^) mice, while their littermates *SMACreER:Ai47* served as control mice. Total RNA was extracted from the adult mouse cerebral cortex, cultured primary leptomeningeal aSMCs, sorted EGFP^+^ aSMCs from *SMACreER:Ai47* or *SMACreER:Ai47:Grin1*^fl/fl^ triple transgenic mouse brains, the adult human cortical tissues, HB-vSMCs and HA-vSMCs, respectively, using TRIzol (Sangon Company, B511311). cDNA was synthesized using the HiScript III 1st Strand cDNA Synthesis Kit (Vazyme, R312-02). Each reaction comprised a total volume of 20 μl containing 2 μl of cDNA, 10 μl of Green Taq Mix (Vazyme, P131-03), each primer pair (Supplementary Table 1) at 0.5 μM (Tsingke) and 6 μl of ddH_2_O.

### RNAscope mRNA in situ hybridization assay

According to the manufacturer’s protocol, the RNAscope assay was performed using the RNAscope Multiplex Fluorescent Detection Reagents v2 Kit (323110, Advanced Cell Diagnostics). Briefly, frozen brain sections from adult *SMACreER:Ai14* were baked at 60 °C for 1 h, rinsed with PBS, and treated with hydrogen peroxide at room temperature for 10 min. The target retrieval was performed at 98–102 °C for 5 min, followed by protease plus (322331) treatment at 42 °C for 30 min. The *Grin1* RNAscope probe (431611) was then hybridized at 42 °C for 2 h. Then, the slices were subjected to RNAscope amplification and chromogenic detection (Opal 570, FP1488001KT, PerkinElmer). The slices were stained with DAPI, mounted with an antifade mounting medium, and imaged with a fluorescence microscope (Carl Zeiss LSM 800) acquired by Zeiss ZEN software (blue edition, version 3.6).

### Western blotting

Proteins were extracted from the sorted EGFP aSMCs from *SMACreER:Ai47* or *SMACreER:Ai47:Grin1*^fl/fl^ transgenic mouse brain or adult mouse cortical tissues, respectively, in ice-cold RIPA lysis buffer (CW2333S) containing protease inhibitor cocktail (CW220). The protein concentration was determined using the bicinchoninic acid method (CW2011), and then the protein samples were boiled with SDS–PAGE loading buffer (CW0027S). All the reagents mentioned above used for sample preparation were obtained from CWBiotech. Samples containing equal amounts of protein (40 μg) were loaded on 8% SDS–PAGE gels and separated at 120 V for 90 min. The proteins were transferred to 0.45-μm polyvinylidene difluoride membranes (Millipore Corporation, IPVH00010) at 400 mA for 120 min. After the blots were blocked with 5% nonfat milk in 0.5% Tween-20 in TBST for 1 h at room temperature, they were incubated with primary antibodies against GluN1 (1:2,000 dilution; Merck Millipore, MAB363), α-SMA (ABclonal Technology, A1011; 1:1,000 dilution) and β-tubulin (ABclonal Technology, AC021, clone AMC0498; 1:5,000 dilution) overnight at 4 °C. On the second day, they were incubated with horseradish peroxidase-conjugated secondary antibodies (1:5,000 dilution; CWBiotech, CW0102/0103). Each experiment was performed at least three times with lysates obtained from different sample preparations.

### Immunofluorescence staining

To determine the subcellular localization of GluN1 in sorted aSMCs or isolated arterioles, immunofluorescence staining was performed. Primary aSMCs were fixed in 4% PFA and 4% sucrose at room temperature for 10 min, and then the cell membrane was permeabilized in 0.3% Triton X-100 in PBS. The cells were blocked with 10% BSA for 30 min and then incubated overnight at 4 °C with an anti-GluN1 antibody (Merck Millipore, MAB363, clone 54.1; 1:500 dilution). After rinsing in PBS, the cells were incubated with a corresponding Alexa Fluor 488-conjugated goat anti-mouse IgG antibody (Invitrogen, A-11034; 1:1,000 dilution) for 2 h at room temperature. Confocal imaging (Carl Zeiss LSM 800) was performed after the cells were mounted with an antifade mounting medium. The raw integrated intensity of GluN1 was measured with ImageJ software.

A Cy3-conjugated anti-α-SMA antibody (Sigma-Aldrich, C6198, clone 1A4; 1:200 dilution) or a fluorescein isothiocyanate (FITC)-conjugated anti-α-SMA (Sigma-Aldrich, F3777, clone 1A4; 1:200 dilution) was used to identify aSMCs. An Alexa Fluor 488-conjugated anti-Tau antibody (Merck Millipore, MAB3420A4, clone PC1C6; 1:200 dilution) was applied to identify neuronal axons. The PSD95 antibody (Merck Millipore, MAB1598, clone 7E3-1B8; 1:500 dilution), NeuN antibody (Merck Millipore, ABN90P, clone A60; 1:500 dilution), Kcnmb1 antibody (Alomone labs, APC-036; 1:200 dilution), vGluT1 (Synaptic Systems, 135311, clone 317D5; 1:500 dilution) antibody staining were also performed. These antibodies of CaMKIIα (Invitrogen, PA5-19128; 1:500 dilution), CD31 (BD Biosciences, 557355, clone MEC13.3; 1:500 dilution), Collagen I (Abcam, ab34710; 1:500 dilution), PDGFRα (R & D systems, AF1062; 1:500 dilution), Smoothelin (Abcam, ab219652; 1:200 dilution) and CD13 (R & D systems, AF2335; 1:200 dilution) were also conducted for immunofluorescence staining. Confocal imaging was acquired by Zeiss Zen software (blue edition, version 3.8).

### Colocalization measurements

Colocalization was defined as when the spatial overlapping correlation between these two fluorescence signals was better than what would have been expected by chance for this image. Specifically, the PSD95, Kcnmb1, α-SMA or vGluT1 channel was fixed, and the GluN1 channel was randomized by moving point-spread-function sized chunks of the image to random locations in a new random test image. The colocalization was determined when the *P* value of statistical significance calculated by the Costes test equaled 1.00. A *P* value of 1.00 means that none of the randomized images had better Pearson’s correlation coefficient (*r*) than that between the two real PSD95 and GluN1, Kcnmb1 and GluN1, α−SMA and GluN1, or vGluT1 and GluN1 channels. The *P* value was obtained after this test was performed 100 times.

### Immunogold staining

After anesthesia with sodium pentobarbital, mice were transcardially perfused with PBS and fixative buffer (3% PFA and 0.2% glutaraldehyde in 0.1 M phosphate buffer, pH 7.4). The brain was removed, stored in 2% PFA overnight, then cut into 60-μm coronal sections. The sections were free-thawed in 30% sucrose, incubated in 5% normal goat serum (NGS) for 1 h, then in GluN1 antibody (Merck Millipore, MAB363, clone 54.1; 1:500 dilution) supplemented with 1% NGS overnight at 4 °C. After washing twice in PBS and twice in PBS-BSA, the sections were incubated in goat anti-mouse IgG-ultrasmall gold conjugate (1:100 dilution) diluted in 1% NGS overnight at 4 °C. After washing thrice in PBS, postfixed in 1% glutaraldehyde in PBS for 10 min, and washing again, the immunogold signal was strengthened with silver for 7 min at room temperature in the dark. The reaction was stopped by washing thrice in ddH_2_O. After thrice washes in PBS, the sections were postfixed in 0.5% osmium tetroxide for 10 min, dehydrated in ascending series of ethanol dilutions, treated with acetone, embedded in resin and polymerized for 48 h at 60 °C. Serial ultrathin sections were cut, collected on single-slot grids, and observed by TEM (Thermo Scientific, Talos L120C G2).

### Pharmacology

For in vitro experiments, a series of doses of glutamate (0.5, 1 and 10 mM, Sigma-Aldrich, 49621) were utilized to stimulate aSMCs in vitro in the presence of glycine (a GluN receptor coagonist, 200 μM; Sigma-Aldrich, V900144). The GluN receptor antagonist D-AP5 (500 μM, Tocris Bioscience, 0106) was also used to inhibit the effect of glutamate. All of these drugs were dissolved in the HEPES-buffered extracellular solution (ECS) containing 150 mM NaCl, 5 mM KCl, 2 mM CaCl_2_, 1 mM MgCl_2_, 10 mM HEPES and 10 mM glucose (pH adjusted to 7.4 with sodium hydroxide) for approximately 1 h at 37 °C before calcium living imaging.

For in vivo experiments, D-AP5 (500 μM), COX2 antagonist (100 μM, NS-398, MedChemExpress) or PAX (100 μM) was administered by intracisternal injection 15 min before whisker stimulation or 2P optogenetic stimulation.

### Electrophysiological whole-cell patch-clamp recording of aSMCs

Cultured leptomeningeal or parenchymal aSMCs were prepared and subjected to continuous perfusion with aCSF consisting of 124 mM NaCl, 2.5 mM KCl, 1.25 mM NaH_2_PO_4_, 1.3 mM MgSO_4_, 26 mM NaHCO_3_, 2 mM CaCl_2_ and 20 mM d-glucose, equilibrated with 95% O_2_ and 5% CO_2_. In some experiments, Mg^2+^-free aCSF was used.

Whole-cell patch-clamp recordings were conducted with borosilicate glass electrodes filled with an internal solution containing 130 mM K-gluconate, 10 mM HEPES, 5 mM KCl, 2 mM MgCl_2_, 2 mM NaCl, 1 mM MgSO_4_, 0.2 mM EGTA, 10 mM sodium phosphocreatine, 4 mM Na_2_-ATP and 0.4 mM GTP-Tris adjust to pH 7.2 with 1 M KOH. Electrode resistance in the bath solution was maintained at 5–7 MΩ, and series resistance was monitored continuously and kept stable within 20%. Visualized whole-cell recordings were obtained from cultured aSMCs using an Axopatch 700B amplifier (Axon Instruments), and the signals were digitized through a Digidata-1550 interface (Axon Instruments) for data acquisition and analysis using pClamp 11.2 (Axon Instruments). The whole-cell currents of recorded aSMCs were observed for at least 10 min to reach stability before application of drugs in voltage-clamp mode via bath perfusion/puff application.

For recording puff application of drugs that induce postjunctional currents mediated by GluRs, a glass pipette was placed rostrally to the recording electrode at the same depth as the recorded cell.

To explore the underlying receptor and ionic mechanisms, selective GluR antagonists NBQX (AMPA/kainate receptor antagonist, 20 μM; Tocris) and D-AP5 (NMDA receptor antagonist, 50 μM; Tocris), as well as the selective BK channel antagonist PAX (10 μM; Tocris) were used. The receptor antagonist or ion channel blocker was administered for at least 15 min before its effect was observed.

### Calcium imaging

In vitro calcium imaging of aSMCs was performed with a Nikon MC-LC1 spinning-disk laser confocal microscope with TIRF (Nikon) using a ×40 water-immersion objective (NA = 1.15) with a perfect focusing system. Images (size of 336 × 336 μm^2^) were recorded every 1.5 s. Each aSMC was imaged for at least 3 min after drug application. The frequency of Ca^2+^ events (Ca^2+^ sparks and waves) in aSMCs were calculated using NIS-Elements AR analysis software as previously described with a few modifications^67^ and manual analysis. Multiple ROIs with a size of 1.54 × 1.54 μm^2^ were chosen in each image to analyze the fluorescence intensity (*F*) over time. The baseline *F*_0_ was determined by averaging ten images without Ca^2+^ sparks. A Ca^2+^ spark was identified as a localized increase in Δ*F/F*_0_ greater than 0.2. Ca^2+^ waves were defined by an Δ*F/F*_0_ elevation > 0.2 that propagated for more than 20 μm. Ca^2+^ spark and wave frequency were calculated for each cell.

*pLenti-CMV-GCamp6s-2A-Tdtomato* (5.18 × 10^8^ transducing units per ml; Obio Technology) and *AAV2/9-hSyn-ChR2-EGFP* (5 × 10^12^ viral genomes/ml; Taitool Bioscience) were added to co-cultured neurons and cerebral vascular smooth muscle cells (HB-vSMCs or primary pial or parenchymal aSMCs from *SMACreER:Ai14* mice). GCamp6, which was under the control of the *CMV* promoter, was expressed in both aSMCs and neurons, while ChR2, which was driven by the *hSyn* promoter, was expressed only in neurons. A laser was exploited to activate ChR2 and detect GCamp6s in vitro at 488 nm. *AAV2/9-CMV-GCamp6s* (5 × 10^12^ viral genomes/ml) was used to identify Ca^2+^ events in vivo and was detected with a 920-nm or 960-nm laser by 2P live imaging.

### Cranial virus injection

Various recombinant adeno-associated virus (AAV) vectors, that is, *AAV2/9-hSyn-EGFP*, *AAV2/9-hSyn-ChR2-EGFP*, *AAV2/9-CaMKIIα-ChR2-mCherry*, *AAV2/9-CaMKIIα-mCherry* or *AAV2/9-CMV-GCamp6s* with a titer of 5 × 10^12 ^viral genomes/ml were injected into 2- to 4-month-old mice. The viruses were purchased from Taitool Bioscience Company. We used a microsyringe pump (Xinglin Lifescience Tech, LSS SH-01C) and a mouse stereotaxic instrument (Xinglin Lifescience Tech, LSS SMO-10B) to inject the viruses into the primary somatosensory cortex (SSp-II) at a speed of 50 nl min^−1^ (Extended Data Fig. 3a–h and Supplementary Fig. 12). The injection coordinates were as follows: AP, 0 mm; ML, 2 mm; DV, −0.5 to −1 mm. Subsequent experiments were conducted at least 3 weeks after the injection.

### Single-photon and 2P optogenetic stimulation

Optogenetic stimulation was achieved by either single-photon activation in vitro or 2P activation in vivo. Laser powers at each wavelength and power setting were measured at the objective site with an optical power meter (PM100D, Thorlabs) before each experiment. To activate virus-transduced neurons expressing ChR2 in vitro, we applied a laser at a wavelength of 488 nm, a mean laser power (under a ×40 water-immersion objective (Nikon, NA = 1.15) of 10–20% (1–2 mW) and 10 Hz and a duration of 50 ms using a spinning-disk laser confocal microscope. In vivo, the axon terminals of pyramidal neurons infected with *AAV2/9-CaMKIIα-ChR2-mCherry* in somatosensory cortex (SSp-II) were activated by a 2P laser (Supplementary Fig. 12a,b). We used an optical meter to determine the power of the wavelengths of 800 nm, 920 nm, 960 nm and 1,100 nm at the ×25 immersion objective (Olympus, XLPLN25XWMP 2, NA = 1.05; Supplementary Fig. 12c). The 920-nm and 960-nm powers resulted in similar powers, and thus we used both wavelengths in this study (Supplementary Fig. 12c). The laser powers were positively correlated with the degree of axonal activation. We used the 25-mW power for basal frame imaging as it was unable to effectively activate neurons, and we used the 45-mW and 80-mW powers to perform optical stimulation^68^, as they can activate neurons (Supplementary Fig. 12d and Supplementary Video 6). The stimulation paradigm of optogenetics was performed in frame or line scanning modes. The frame scanning at the galvanometer scanner with an image size of 512 × 512 pixels, dwell time of 4 μs and duration of 2 min for the broad activation of ChR2-positive neuron was executed. The line scanning at stimulation mode with a dwell time of 20 μs, frequency of 20 Hz and duration of 0.5 s for local activation of ChR2-positive neuronal soma, dendrite or axon were performed. As a control, the comparable fluorescence intensity of an intraluminal FITC signal, which can be excited by the 920-nm and 960-nm lasers, was measured at these three powers, reflecting the optimal control of the imaging parameters simultaneously.

Artificial SD was induced by frame scanning using a 920-nm laser at 100 mW for 6 min continuously with a dwell of 8 μs per pixel. If necessary, this stimulation paradigm was repeated until the artificial SD induction was successful. Normally, no repeated stimulation was required unless there were not enough ChR2-positive neurites within the ROI. The number of repetitions covaried with the variation of the ChR2 virus infection rate. In general, the more ChR2^+^ neurites, the fewer repeated stimulations. The ROIs were excluded if four repeats still failed to induce SD.

### Detection of optogenetic-induced heat injury

Optogenetic stimulation relies on high illumination powers, whether or not heat injury is induced by high-power illumination in the brain. Anesthetized mice were exposed to 2P optogenetic stimulation protocols: continuous frame scanning for 5 min at a wavelength of 920 nm with 80 mW power. The microscope’s focal plane was 100–200 μm below the pia. After 24 h, mice were transcardially perfused with 4% PFA in PBS. The brain was postfixed in the same solution for 4 h, followed by washing in PBS. Coronal sections were cut throughout the illuminated region and incubated with primary antibodies supplemented with 1% BSA and 0.1% Triton X-100. Alternating sections were labeled for glial fibrillary acidic protein (Thermo Fisher Scientific, UC276149, clone 2.2B10; 1:500 dilution), Iba1 (Wako Pure Chemical Industries, 019-19741; 1:500 dilution) and cleaved caspase-3 (Cell Signal Technology, 9661; 1:500 dilution). Fluorescence images were acquired by epifluorescence microscope.

### Live imaging of vascular dynamics by 2P microscopy

We injected FITC-conjugated dextran (2,000 KDa, 10 mg ml^−^^1^; Sigma-Aldrich, FD2000S) or Tetramethyl rhodamine isothiocyanate (TRITC)-dextran (MW 500 kDa, 0.05 mg per kg body weight, Sigma-Aldrich, 52194) via the tail vein to label blood, or Alexa Fluor 633 (5 mg per kg body weight; Thermo Fisher, A30634) via the tail vein to label arteriole elastin. The diameter and blood flow of p-arterioles were recorded by 2P imaging. Imaging acquisition was carried out using a laser at a wavelength of 1,100 nm, 960 nm or 920 nm, in a field of view of 512 × 512 pixels (pixel dwell time of 4 μs and pixel size of 0.994 μm). Live imaging was conducted at a depth of 20–300 μm from the pial surface. Fiji software converted the frame-scanned images to spatially optimized line-scanned mode images. P-arterioles were identified by DsRed-positive ring-shaped aSMCs or Alexa Fluor 633-positive vessels. Transverse section line scanning was used to assess the dynamic change in vascular diameter. The vascular diameter was calculated as the full width at half maximum of a time average of a line scanning image across the width of a vessel^69^. Penetrating arteriolar blood flow was determined as the ratio of the delta intensity (∆*F*) to baseline intensity (*F*_0_) of FITC-dextran or TRITC-dextran fluorescence in the bloodstream.

### Intracisternal injection

Anesthetized mice were fixed in a stereotaxic apparatus. A 30-gauge needle connected to PE-10 tubing filled with saline, tracers and drugs poked into the CM. For intracisternal injection, 20 μl of CSF tracer was injected at a 2 μl min^−1^ flow rate for 10 min with a syringe pump (KD Scientific Legato 130). The FITC-conjugated dextran (2,000 KDa, 10 mg ml^−1^, Sigma-Aldrich, FD2000S) was diluted in saline and used as a fluorescent CSF tracer. To verify a successful injection, the FITC tracer was injected with D-AP5, PAX or NS-398.

### Whisker stimulation and laser speckle contrast imaging

Whisker stimulation (4 Hz, 1 min) was performed on the right side of each anesthetized mouse using a foam brush controlled by a servo engine, which was manually turned on and off with approximately a 1-s deviation. CBF in mice with the scalp surgically opened was continuously recorded using LSCI (RFLSI III, RWD Life Sciences). For each mouse, five technical trials were acquired, of which the largest and the smallest CBF variation data were excluded, and the remaining three trials were averaged.

### Transient MCAO

The process of transient MCAO was induced as follows. Briefly, 8- to 12-week-old male mice (25–30 g) were anesthetized with pentobarbital sodium. A midline neck incision was made, and the right common carotid artery was ligated. An intraluminal suture (Doccol, 7023910PK5Re) was inserted into the right common carotid artery and advanced to the carotid bifurcation to allow middle cerebral artery occlusion. At 2 h after occlusion, the suture was withdrawn, by which reperfusion was initiated. The mice were monitored until they fully recovered from the MCAO surgery, and then returned to their home cages. Body weight and mortality rate after the surgery were closely monitored.

### RBT

The RBT is used to evaluate the motor, balance and sensory functions of animals. The RBT was performed 1 d before the induction of stroke and on post-stroke days 1, 2, 4, 7, 10, 12, 14 and 18. Briefly, before testing, the mice were subjected to training sessions for three consecutive days. Each training day consisted of three consecutive sessions where the mice traveled across the entire beam. During the test, the mice were placed on a beam rotating at 3 r.p.m., and the fall frequency, average speed and total travel distance were recorded and analyzed.

### Statistics and reproducibility

Representative images were replicated independently with similar results in at least three capillaries, three first branches from p-arterioles, three p-arterioles, ten cell culture dishes, six animals or three independent experiments. No statistical methods were used to predetermine sample sizes, but our sample sizes are similar to those reported in previous publications^70,71^. Data distribution was assumed to be normal, but this was not formally tested. Animals in test and control groups were littermates and selected randomly. The investigator was not blinded to most of the experiments in vitro because the cell and isolated p-arteriole experiments were performed using a pipeline applied equally to all conditions and replicates. However, the investigators were blind to the behavioral test experiments and brain atrophy quantification when animals were littermates. All the tests were conducted first, and the genotype was identified later. Exclusion occurred due to poor imaging quality and an unsuccessful MCAO model. For more specific exclusions, please see the Reporting Summary. All data were analyzed with GraphPad Prism 9 (version 9.5.0) and were presented as the mean ± s.e.m. Unpaired, two-tailed, Student’s tests were performed. Data for the two groups were parametrically analyzed using Welch’s correction for data with unequal variances. In addition, one-way ANOVA followed by the Bonferroni multiple-comparisons test was used to assess all differences between other groups. A *P* value < 0.05 was considered significant.

### Reporting summary

Further information on research design is available in the Nature Portfolio Reporting Summary linked to this article.

## Online content

Any methods, additional references, Nature Portfolio reporting summaries, source data, extended data, supplementary information, acknowledgements, peer review information; details of author contributions and competing interests; and statements of data and code availability are available at https://doi.org/10.1038/s41593-023-01515-0.

## Supplementary information

Supplementary Information Supplementary Figs. 1–17

Reporting Summary

Supplementary Tables 1 and 2

Supplementary Video 1 Neuronal axons pass through gaps in astrocytic endfeet and form the NsMJ with aSMC on p-arteriole.

Supplementary Video 2 Glu-NsMJ identified by CLEM.

Supplementary Video 3 The formation of NsMJ in vitro.

Supplementary Video 4 Calcium events of aSMC induced by glutamate stimulation.

Supplementary Video 5 Functional NsMJ activated by optogenetic in vitro.

Supplementary Video 6 Glutamatergic neuronal axon successfully activated by 2P optogenetic in vivo.

Supplementary Video 7 GluN1 genetic perturbation in aSMCs abrogates the p-arteriolar dilation induced by ChR2-positive glutamatergic neuronal axon activation.

Supplementary Video 8 Genetic disruption of GluN1 in aSMCs attenuates vasoconstriction following neural SD.

Supplementary Video 9 Genetic perturbation of GluN1 in aSMCs prevents calcium overload in aSMCs following ischemic stroke.

## Source data

Source Data Fig. 1 Statistical source data.

Source Data Fig. 2 Statistical source data.

Source Data Fig. 3 Statistical source data.

Source Data Fig. 4 Statistical source data.

Source Data Fig. 5 Statistical source data.

Source Data Fig. 6 Statistical source data.

Source Data Fig. 7 Statistical source data.

Source Data Fig. 8 Statistical source data.

Source Data Extended Data Fig./Table 2 Statistical source data.

Source Data Extended Data Fig./Table 3 Statistical source data.

Source Data Extended Data Fig./Table 5 Statistical source data.

Source Data Extended Data Fig./Table 6 Statistical source data.

Source Data Extended Data Fig./Table 7 Statistical source data.

Source Data Extended Data Fig./Table 8 Statistical source data.

Source Data Extended Data Fig./Table 9 Statistical source data.

Source Data Extended Data Fig./Table 10 Statistical source data.

## Data Availability

All data generated or analyzed in the current study are included in this published article and its Supplementary Information. The raw RNA-seq data for parenchymal aSMCs (six samples: GSM7493520–GSM7493525) and cerebral cortex (six samples: GSM6568826–GSM6568831) conducted in this study have been uploaded to the NCBI Gene Expression Omnibus (GEO) database under accession number GSE213026. The following publicly available datasets were used: single-cell RNA-seq database of mouse brain vasculature from the Betsholtz laboratory^23^ (https://betsholtzlab.org/VascularSingleCells/database.html) and the GEO database under accession number GSE98816. Bulk RNA-seq data of brain cells from Zhang et al.^71^ (http://www.brainrnaseq.org/) are deposited in the GEO under accession number GSE52564. Source data are provided with this paper.

## Code availability

The arteriole diameter changes before and after optogenetic stimulations, or during stroke condition were analyzed using custom code performed in MATLAB (https://github.com/JialabEleven/new_vessel_diameter_detection/) and uploaded in GitHub. The ∆*F/F*_0_ of calcium fluorescence intensity in vitro that is shown in Supplementary Video 4 was visualized by custom code performed using MATLAB (https://github.com/JialabEleven/calcium_spark_adjustment/) uploaded in GitHub.

The MATLAB (version 2019a) code of AQuA (https://github.com/yu-lab-vt/AQuA/) under open source in GitHub was applied to analyze the frequency of calcium events in vivo.
