# Author Correction: Synaptic-like transmission between neural axons and arteriolar smooth muscle cells drives cerebral neurovascular coupling

**DOI:** 10.1038/s41593-026-02474-y

**Published:** 2026-09-25

**Authors:** Dongdong Zhang, Jiayu Ruan, Shiyu Peng, Jinze Li, Xu Hu, Yiyi Zhang, Tianrui Zhang, Yaping Ge, Zhu Zhu, Xian Xiao, Yunxu Zhu, Xuzhao Li, Tingbo Li, Lili Zhou, Qingzhu Gao, Guoxiao Zheng, Bingrui Zhao, Xiangqing Li, Yanming Zhu, Jinsong Wu, Wensheng Li, Jingwei Zhao, Woo-ping Ge, Tian Xu, Jie-Min Jia

**Affiliations:** 1 School of Life Sciences, Fudan University, Shanghai, China; 2 Key Laboratory of Growth Regulation and Translation Research of Zhejiang Province, School of Life Sciences, Westlake University, Hangzhou, China; 3 Laboratory of Neurovascular Biology, Institute of Basic Medical Sciences, Westlake Institute for Advanced Study, Hangzhou, China; 4 Westlake Laboratory of Life Sciences and Biomedicine, Hangzhou, China; 5 Laboratory of Neurovascular Biology, School of Life Sciences, Westlake University, Hangzhou, China; 6 College of Artificial Intelligence and Big Data for Medical Sciences, Shandong Academy of Medical Sciences, Shandong First Medical University, Jinan, China; 7 Huashan Hospital, Shanghai Medical College, Fudan University, Shanghai, China; 8 Program in Speech and Hearing Bioscience and Technology, Harvard Medical School, Boston, MA USA; 9 Department of Neurosurgery, Huashan Hospital, Shanghai Medical College, Fudan University, Shanghai, China; 10 Brain Function Laboratory, Neurosurgical Institute of Fudan University, Shanghai, China; 11 Institute of Brain-Intelligence Technology, Zhangjiang Lab, Shanghai, China, Shanghai, China; 12 Shanghai Key Laboratory of Brain Function and Restoration and Neural Regeneration, Shanghai, China; 13 Shanghai Clinical Medical Center of Neurosurgery, Shanghai, China; 14 Department of Anatomy, Histology, and Embryology, School of Basic Medical Sciences, Fudan University, Shanghai, China; 15 Department of Anatomy, Histology, and Embryology, Research Center of Systemic Medicine, School of Basic Medicine, and Department of Pathology of the Sir Run-Run Shaw Hospital, The Cryo-EM Center, NHC and CAMS Key Laboratory of Medical Neurobiology, Zhejiang University School of Medicine, Hangzhou, China; 16 Chinese Institute for Brain Research, Beijing, Beijing, China

**Keywords:** Neuro-vascular interactions, Neurotransmitters, Molecular neuroscience, Neurophysiology

Correction to: *Nature Neuroscience* https://doi.org/10.1038/s41593-023-01515-0, published online 2 January 2024.

In the version of this article initially published, errors were identified in Fig. 1h, j and k, Fig. 8d, associated Source Data files, the Fig. 1 legend and the legend to Supplementary Fig. 4g. The total number of neurovascular smooth muscle junctions (NsMJs) reported in Fig. 1h was corrected from 502 to 499, following the removal of duplicate counts and inclusion of omitted NsMJs. In Fig. 1j, prejunctional boutons were reclassified into three categories based on vesicle content, and the Results text was revised from stating that 80% of NsMJs contained small vesicles and 20% contained dense-core vesicles to stating that 76.4% contained small vesicles only, 16.0% contained dense-core vesicles only and 7.6% contained both. In Fig. 1k, duplicated measurements were removed, resulting in a revision of the reported mean bouton-to-aSMC membrane distance from 200 nm to 205.6 nm. The Fig. 1 legend was updated to clarify sample sizes. In Fig. 8d, duplicated neural signal data were removed and the figure legend revised accordingly. The legend of Supplementary Fig. 4g was amended to clarify that the image shown was a 90° counterclockwise rotation of the image in Extended Data Fig. 4b. The figures, legends, Source Data, text and Supplementary Information have been updated in the HTML and PDF versions of the article. For comparisons, the original figures are available as Supplementary Information accompanying this amendment. These changes do not affect the conclusions of the study.

## Supplementary information

Original and corrected Figs. 1h, j, k, 8d

