# Supplementary material for: Synaptic-like transmission between neural axons and arteriolar smooth muscle cells drives cerebral neurovascular coupling
Source: Nat Neurosci. 2024 Jan 2;27(2):232–48. doi: 10.1038/s41593-023-01515-0 (PMC10849963; doi:10.1038/s41593-023-01515-0)
Supplement: Supplementary file 1 — Supplementary Figs. 1–17 [file 41593_2023_1515_MOESM1_ESM.pdf]

nature neuroscience

Article

https://doi.org/10.1038/s41593-023-01515-0

Synaptic-like transmission between neural axons and arteriolar smooth muscle cells drives cerebral neurovascular coupling

In the format provided by the authors and unedited

Supplementary Fig. 1

Zhang et. al

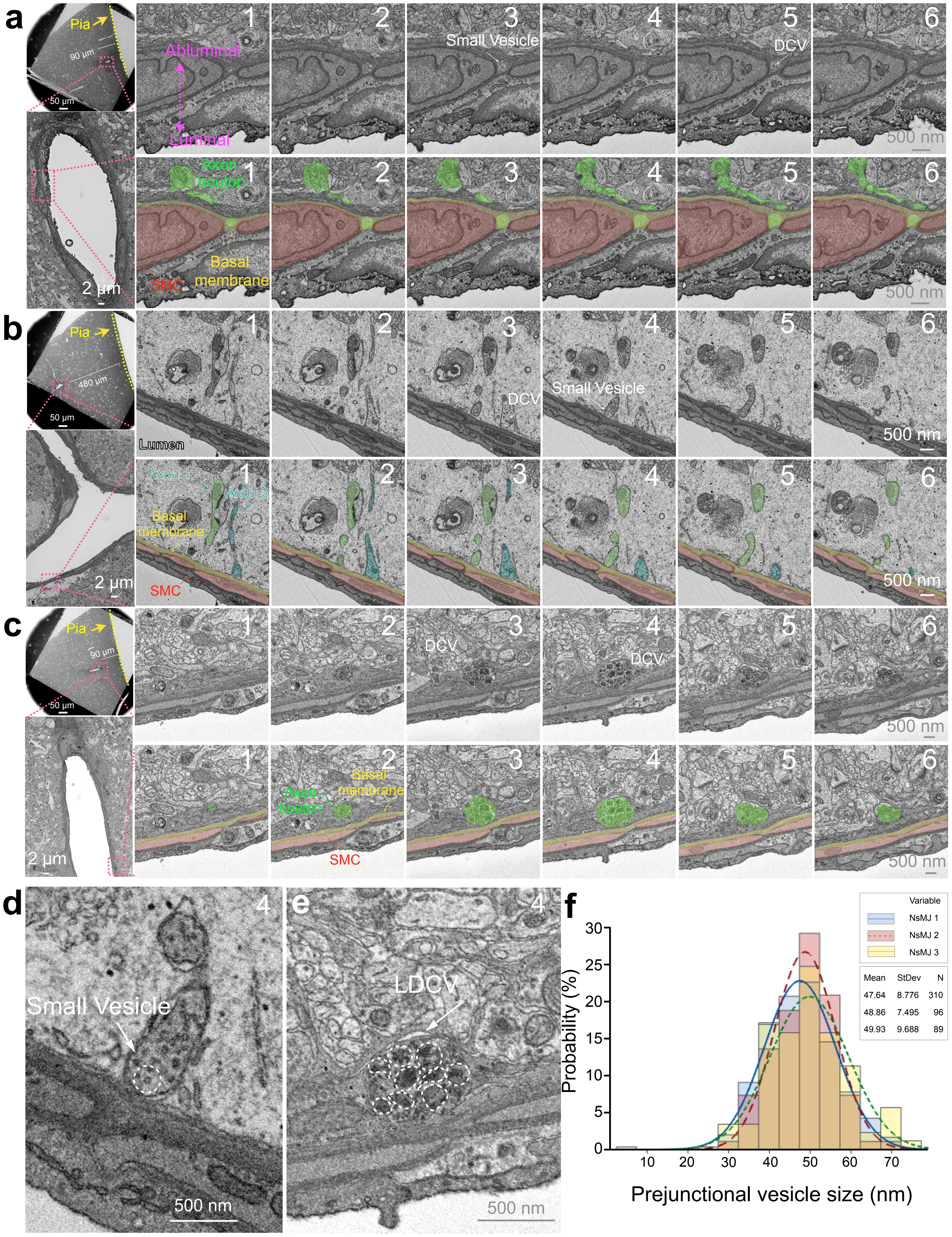

Supplementary Fig. 1 Ultrastructures of different NsMJs containing different types of presynaptic-like vesicles. (**a**-**c**) Additional four NsMJs, each with six serial SBF-SEM images, on the other two p-arterioles (PA-2 and PA-3). **Top left**: Large views of the somatosensory cortex (300 nm/pixel resolution). **Bottom left**: Higher magnification of areas in the dashed box labeled the corresponding top panels with depths of 90 µm or 480 µm from pia. The NsMJ in **a** shows small, clear vesicles and a solitary small dense core vesicle (SDVC) with an average size of approximately 50 nm; the left NsMJ in **b** contains small, clear vesicles only, while the right one in **b** has many SDVCs; the NsMJ in **c** contains large DVCs (LDVCs), with an average size of approximately 120 nm. NsMJs are highlighted with pseudo colors labeling axonal bouton, basal membrane, and aSMCs. (**d**-**e**) Zoomed-in SEM images of NsMJs in **b-4** and **c-4**, Each representative type of vesicle is marked. All representative images of **a**-**e** were replicated independently with 2 p-arterioles from one mouse. (**f**) Histogram graph for the sizes of vesicles from three additional axonal boutons that formed NsMJs, with small, clear vesicles. NsMJ 1, 310 vesicles; NsMJ 2, 96 vesicles; NsMJ 3, 89 vesicles. Averaged distribution of all the vesicles, brown. Fitting curves represent the estimated normal distribution for vesicle sizes near 50 nm in each NsMJ.

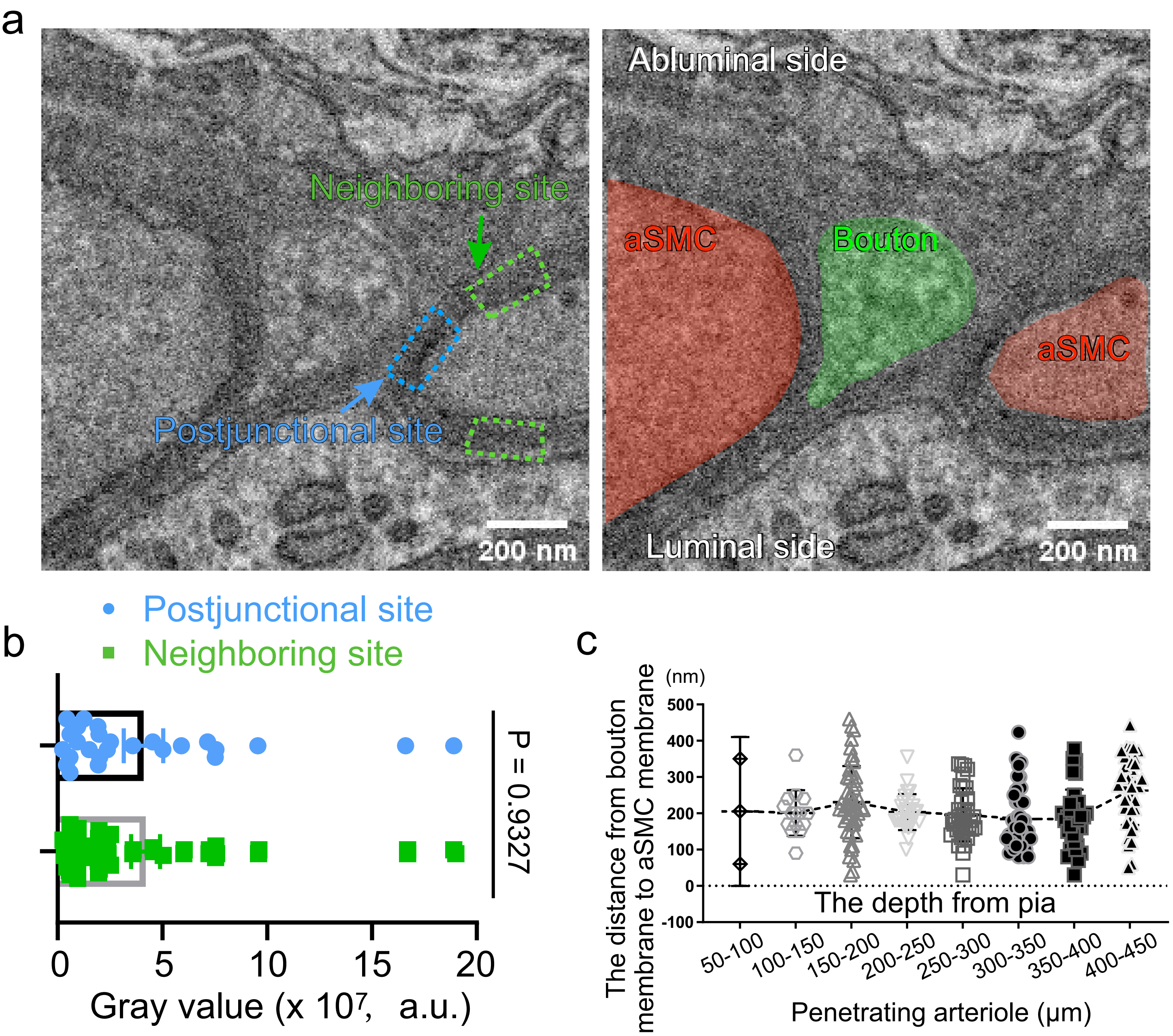
a
Neighboring site
Postjunctional site
200 nm
Abluminal side
aSMC
Bouton
aSMC
Luminal side
200 nm
b
Postjunctional site
Neighboring site
P = 0.9327
0
5
10
15
20
Gray value (x 10^7, a.u.)
c
(nm)
500
400
300
200
100
0
-100
The distance from bouton membrane to aSMC membrane
The depth from pia
50-100
100-150
150-200
200-250
250-300
300-350
350-400
400-450
Penetrating arteriole (μm)

Supplementary Fig. 2 Ultrastructural characterizations of postjunctional membrane and junctional cleft for NsMJs. (**a**) One section of serial SEM sections from an NsMJ from **Supplementary Fig. 1a-5**. Electronic density analysis for the postjunctional site as well as the neighboring sites. (**b**) Density quantification for both sites in **a**. N = 26 postjunctional and 50 neighboring sites of 26 NsMJs from 3 p-arterioles. (**c**) The measurements for the size of the putative junctional cleft of NsMJs per depth (50 μm) from pia. N = 247 NsMJs from 3 p-arterioles. Data are mean ± s.e.m.; nested, unpaired, two-tailed *t*-test.

Supplementary Fig. 3

Zhang et.al

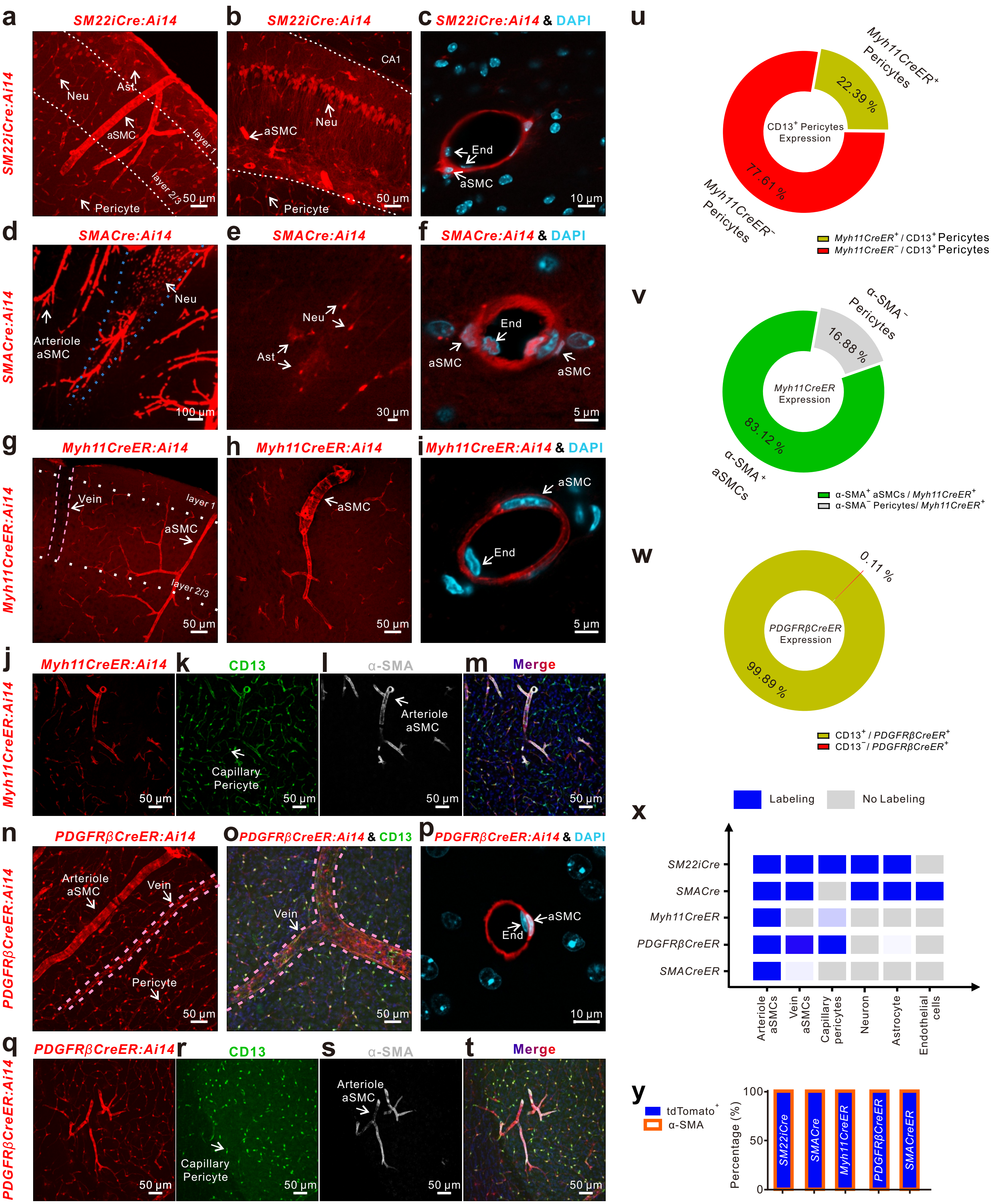

Supplementary Fig. 3 Different specificities to aSMCs in different promoter driver lines. (**a-t**) Representative brain images from *SM22iCre:Ai14* mice (**a-c**), *SMACre:Ai14* (**d-f**), *Myh11CreER:Ai14* (**g-m**), and *PDGFRβCreER:Ai14* (**n-t**). Zeiss Z1 light sheet images of *SMACre:Ai14* mouse brain after tissue clearing (**d-e**) and confocal image of brain slice (**f**). Confocal images of brain sections with immunostaining of anti-a-SMA and/or CD 13 antibodies and counterstaining of DAPI (**j-t**). CD13 antibody labels nearly all aSMCs and pericytes, while a-SMA antibody only labels aSMCs. (**u**) Percentage of tdTomato$^+$ cells in total pericytes. (**v**) Pericyte and aSMC percentages in tdTomato$^+$ cells. (**w**) CD13 marker labeled the vast majority of tdTomato$^+$ cells, including nearly all aSMCs and all pericytes. (**x**) Summary of the labeling specificities of five driver lines. (**y**) The efficiency of labeling aSMCs across the five driver lines. End, endothelial cell; Ast, astrocyte; Neu, neuron. Gray depicts no labeling at all, blue means labeling with high efficiency, and light blue means minimally labeling in **x**. N = 3 mice per promoter driver lines.

Supplementary Fig. 4 Zhang et. al

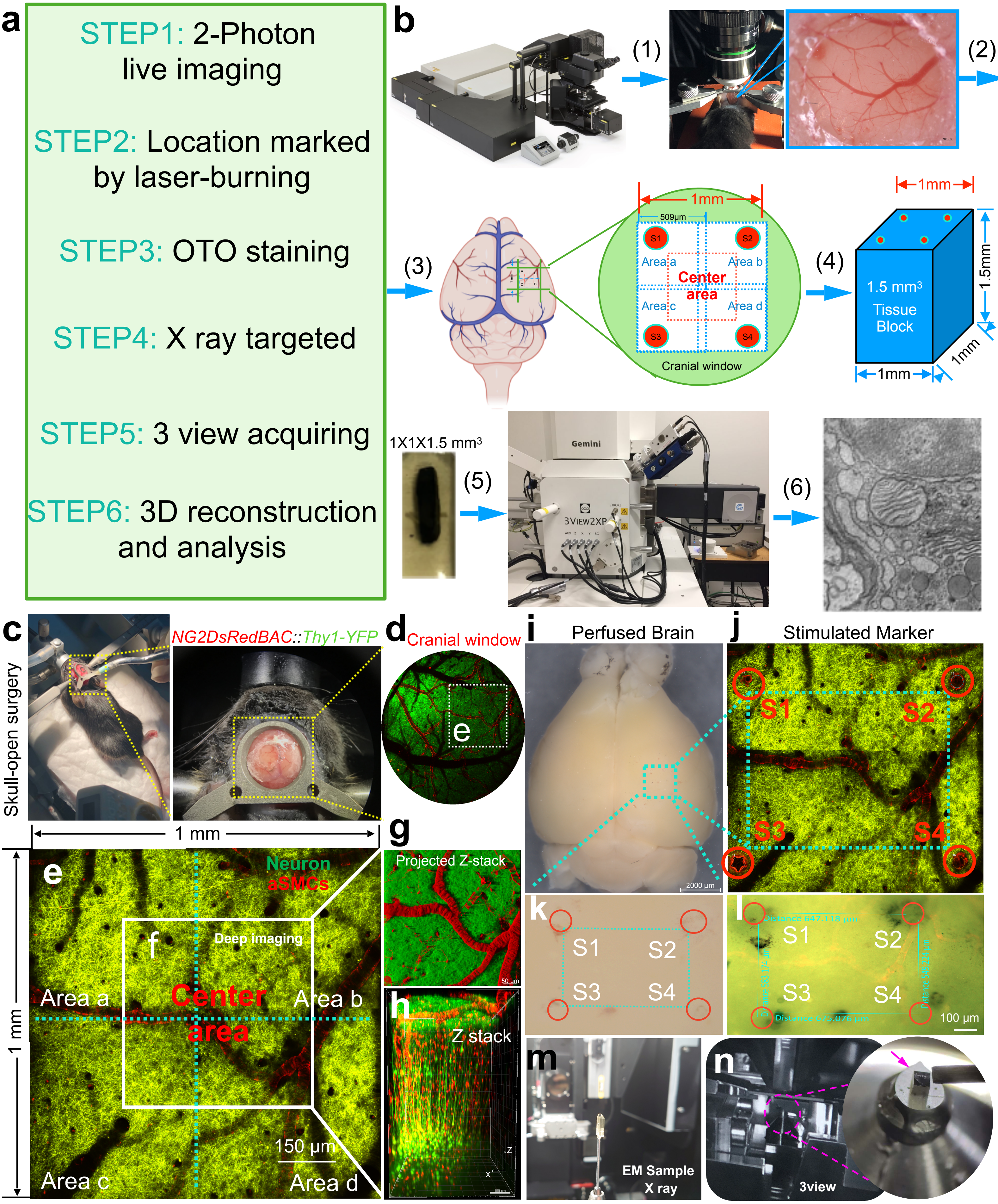

Supplementary Fig. 4 The procedure of conducting two-photon live imaging-based CLEM for the Glu-NsMJ identification. (**a**) Procedure outlines six steps. (**b**) Image-illustration of each step shown in **a**. The schematic brain in **b**-(3) was created with BioRender.com. S1-S4 indicates four laser-burned locations closing to the edge of the stitched four field-of-view (Area a-d) that had been 2P live-imaged, with a center area imaged deeply. A $1\times1\times1.5$ mm^3 tissue block was precisely dissected and subjected to OTO staining and 3view SEM imaging. (**c**-**h**) Images were taken when the mouse was alive. (**c**) The photograph shows the *NG2DsRedBAC:Thy1-YFP* double Tg mouse with a cranial window subjected to 2P live imaging. (**d**) Low magnification image of the DsRed-positive aSMCs and YFP-positive glutamatergic neurons (5X objective). High magnification of live imaging was performed in **e**, which was stitched from 4 field-of-views by $509\times509\times200$ μm^3 (Area a-d). (**f**) 'Center area' was imaged again by $509\times509\times820$ μm^3. The surface rendering of the projected z-stack image (**g**) and the side view (**h**) of the 'center area' in **f**. (**i-l**) Images taken after the mouse was perfused by PFA. N = 3 mice for **e** to **h**, among these 3 samples, two of them proceeded from **i** to **l**. The 4 laser-burned spots (S1-S4) were identified back in the perfused brain (**i**) in both fluorescent (**j**), bright-field image (**k**), and their merged images (**l**). (**m-n**) The specimen on XRM setup and 3view SEM setup. The magenta arrow points to the installation site of the specimen.

Supplementary Fig. 5

Zhang et.al

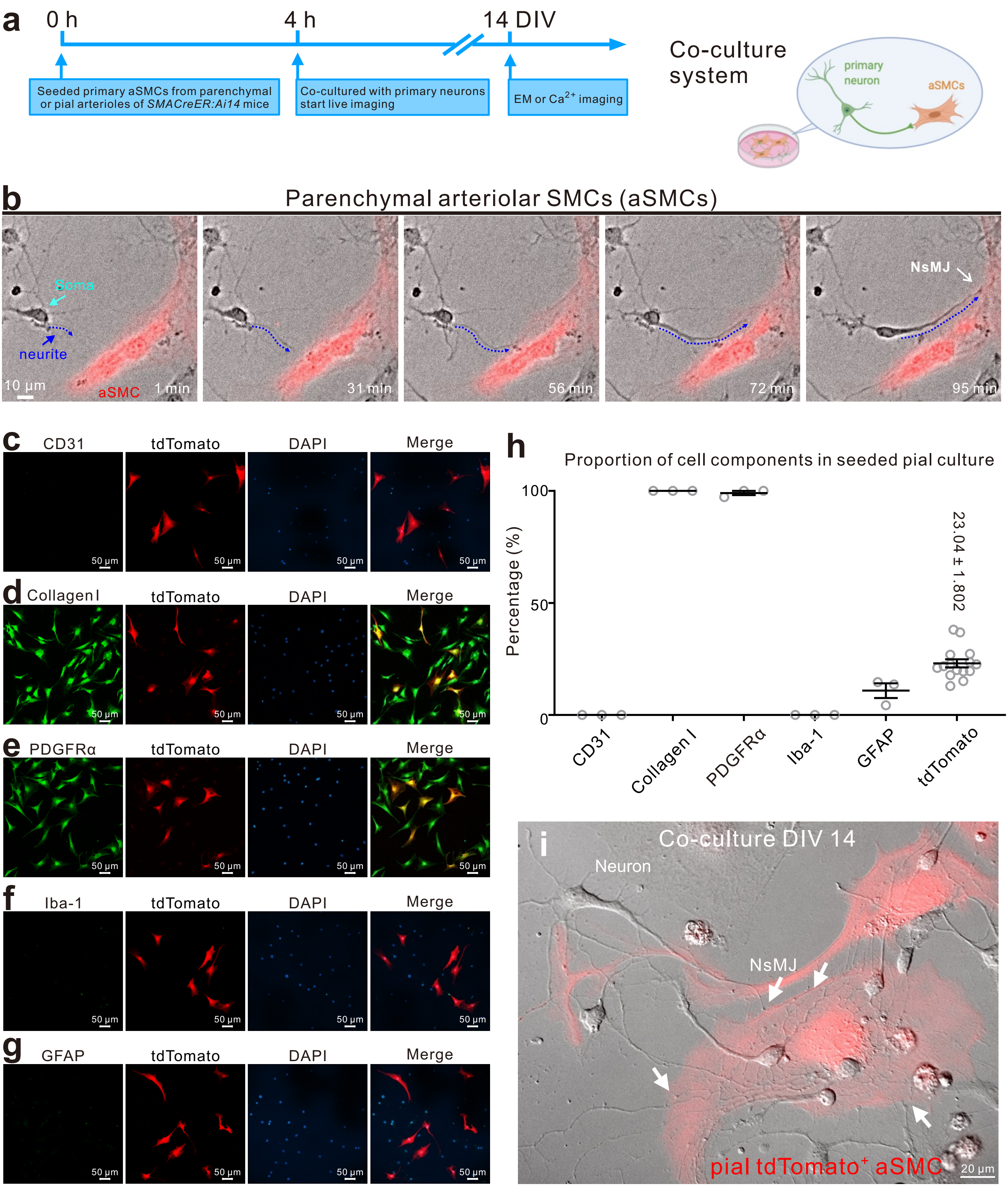

Supplementary Fig. 5 Recapitulation of NsMJ formation and maintenance *in vitro.* (**a**) Illustration of the timeline for generating a co-culture system of primary pial or parenchymal aSMCs of *SMACreER:Ai14* mouse brains with primary neurons. Right panel was created with BioRender.com. One week before adding neurons, tamoxifen was first added to the culture medium to induce tdTomato expression. (**b**) Serial live images of parenchymal tdTomator+ aSMCs with primary neurons. Time-lapse imaging started at 4 hours after seeding neurons and continued for 95 min. The neuronal soma extends a neurite towards aSMCs, forming a potential NsMJ in vitro. N = 18 culture dishes from three independent experiments. (**c**-**h**) Determination of tdTomato^{-} cell identity in the primary mixed leptomeningeal cells culture in SMCM for 14 days when primary neurons were added. Immunofluorescent staining of CD31 (**c**), Collagen I (**d**), PDGFRα (**e**), Iba-1 (**f**), GFAP (**g**). (**h**) Statistic analysis of the proportion of cell components in these mixed cultures in **c**-**g**. N = 30 FOVs from 10 cell culture dishes examined from 3 independent experiments. Data are mean ± s.e.m. (**i**) Epifluorescence with bright field image of the co-culture at DIV 14 for neurons, while in terms of aSMC, it was at DIV 28. White arrows pointed to the putative NsMJs. N = 14 FOVs from 5 dishes from 5 independent co-culture experiments.

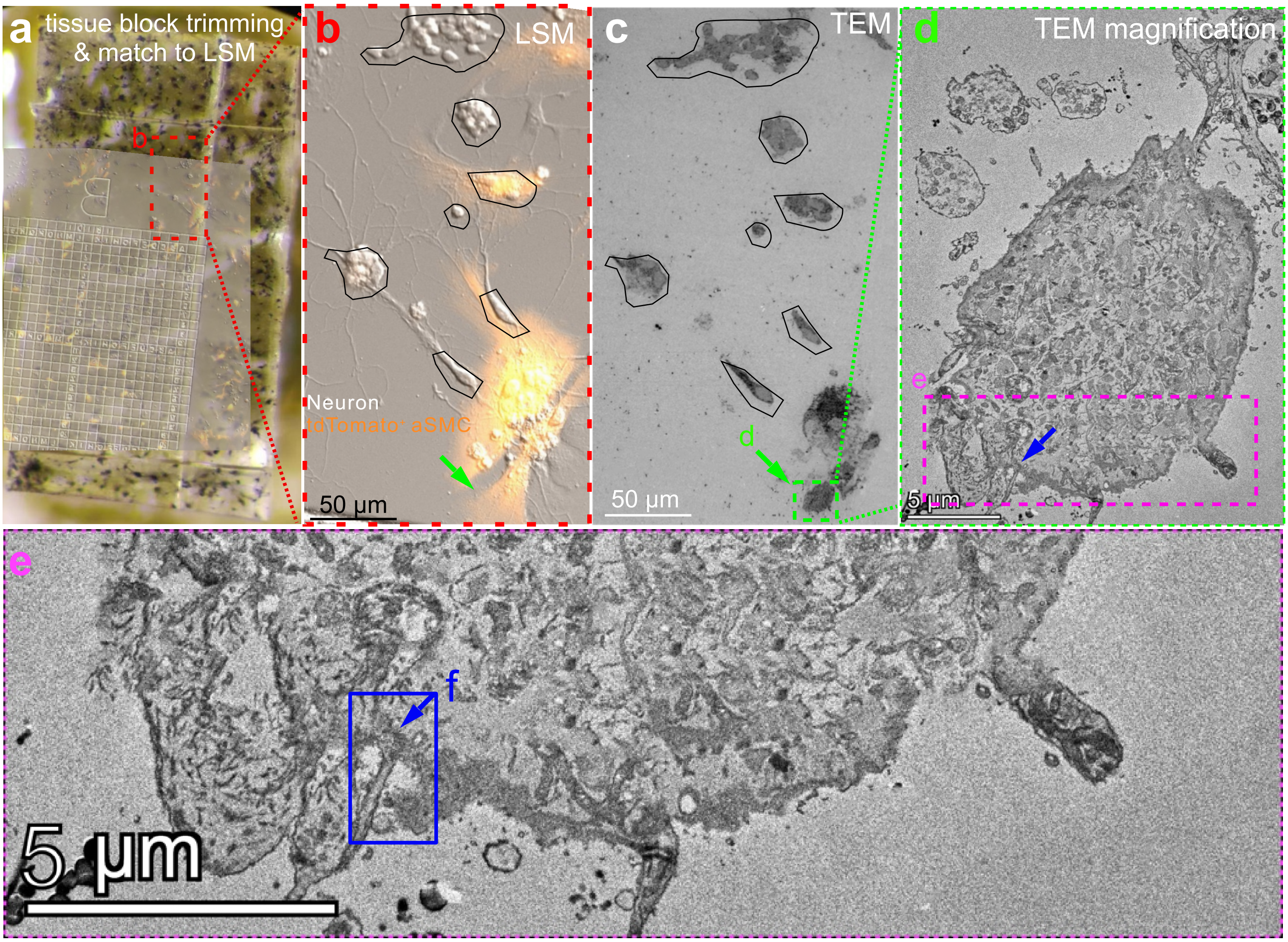
a
tissue block trimming
& match to LSM
b
LSM
Neuron
tdTomato⁺ aSMC
50 μm
c
TEM
d
50 μm
d
TEM magnification
e
5 μm
e
f
5 μm

Supplementary Fig. 6 TEM-CLEM of the primary neuron and the sorted parenchymal tdTomato$^+$ aSMC coculture *in vitro.* (**a-f**) Correlated LSM with TEM for identifying the ultrastructure of NsMJ. (**a**) Trimmed TEM sample block of co-culture matched the correlated fluorescent image. (**b**) Magnified fluorescent image with our targeted tdTomato$^+$ aSMCs pointed by the green arrow. (**c**) Correlated TEM of **b**. (**d**) Magnification of the region in the green box labeled by **d** in **c**. (**e**) High-power image of the region in the magenta box labeled by **e** in **d.** (**f**) The blue arrow pointed NsMJ with higher resolution is shown in **Fig. 2g**. N = 14 FOVs from 5 dishes from 5 independent co-culture experiments.

Supplementary Fig.7 Zhang et.al

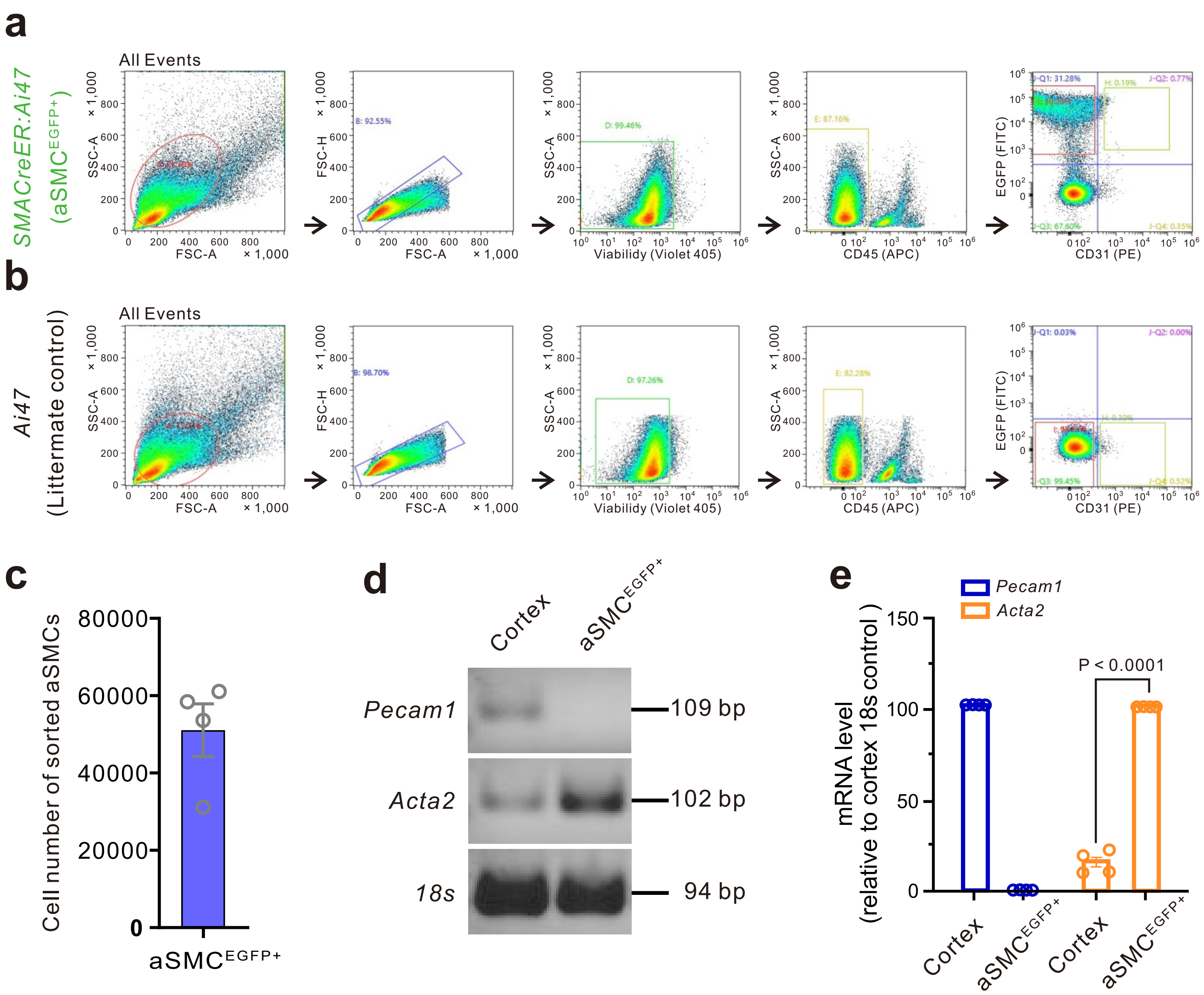

Supplementary Fig. 7 Parenchymal arteriolar SMCs sorting with strict exclusion of EC contaminations. (**a**) Flow cytometry sorted EGFP$^+$ aSMCs derived from enriched parenchymal vessels with minimally containing capillaries. *SMACreER:Ai47* mice received tamoxifen at adult. CD45 and CD31 negative selections were used to exclude contaminations from immune cells and ECs. (**b**) Ai47 single-positive mice were used as control during the flow cytometry. APC stands for allophycocyanin, an intensely bright phycobiliprotein; PE for phycoerythrin. Averaged cell numbers of the obtained parenchymal EGFP$^+$ aSMCs with high purity (**c**). RT-PCR of *Pecam1* (CD31) and *Acta2* (α-SMA) genes in the cortex and sorted EGFP$^+$ aSMCs (**d**). (**e**) Quantification for **d**. N = 4 mice. Data are mean ± s.e.m.; nested, unpaired, two-tailed *t*-test.

Supplementary Fig. 8

Zhang et.al

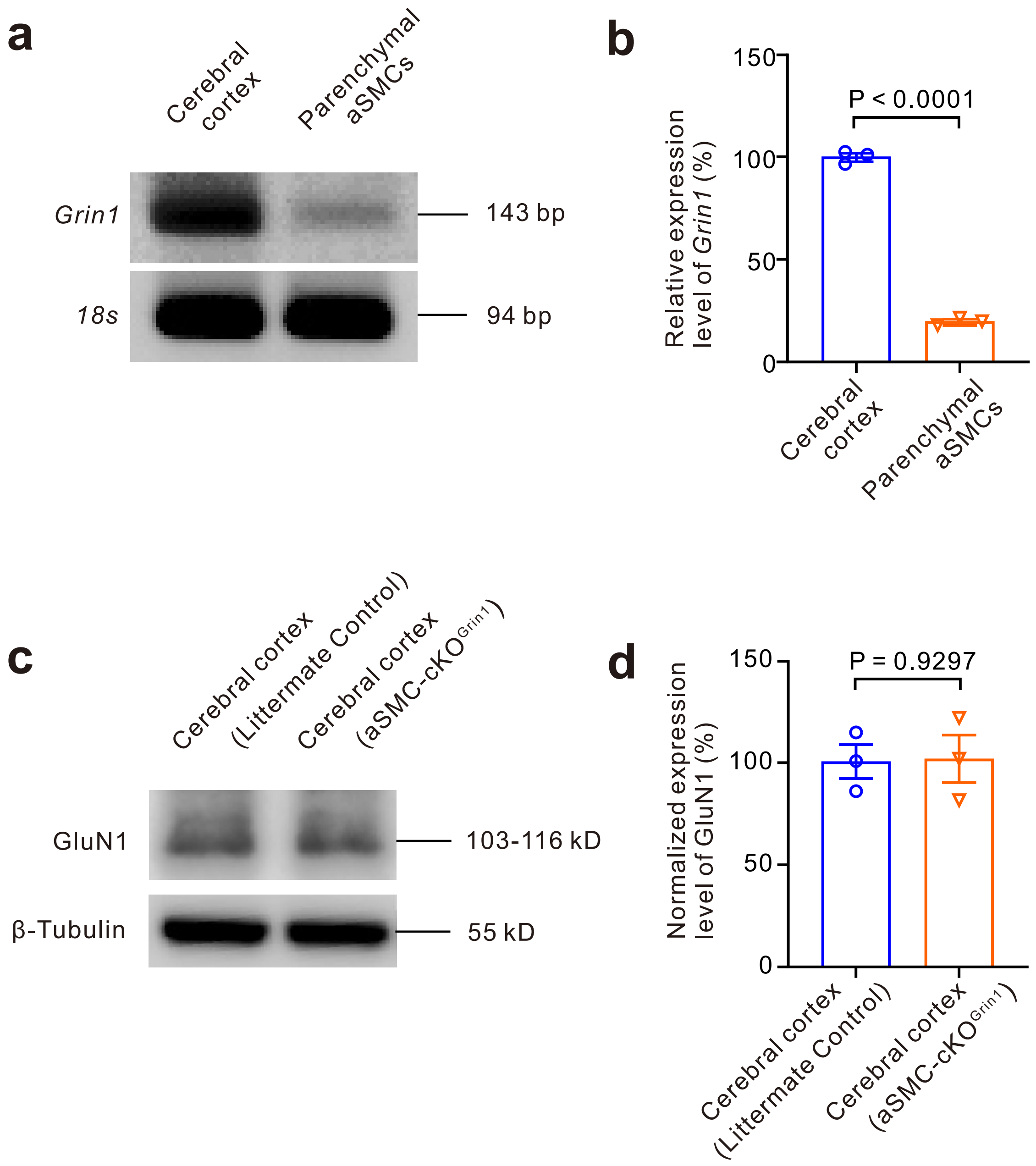

Supplementary Fig. 8 GluN1 expression in parenchymal arteriolar SMCs. (**a**) RT-PCR of *Grin1* gene in cerebral cortex lysates and sorted parenchymal aSMCs from adult brains, with statistic quantification in **b**. N = 3 mice (*SMACreER:Ai47*). (**c**) GluN1 protein with equivalent expression in the cerebral cortex of control (*Grin1*$^{f/f}$) and *aSMC-cKO*^{Grin1} (*SMACreER:Grin1*$^{f/f}$) mice, with statistic quantification in **d**. N = 3 mice for each genotype. Data are mean ± s.e.m.; nested, unpaired, two-tailed *t*-test (**b**, **d**).

Supplementary Fig. 9

Zhang et. al

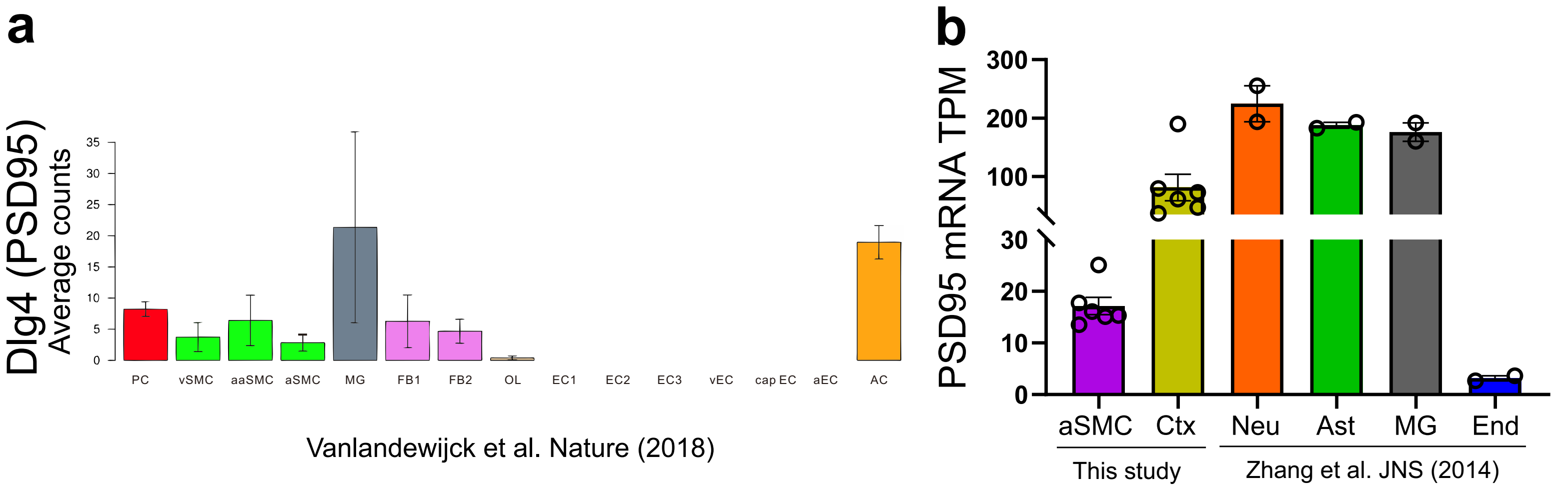

Supplementary Fig. 9 Postsynaptic density 95 (PSD95) mRNA expression in aSMCs. (**a**) Single-cell RNA sequencing data shows PSD95 mRNA expression across mouse brain vascular and glial cells. Data credit: Christer Betsholtz lab in Sweden[23]. https://betsholtzlab.org/VascularSingleCells/database.html. (**b**) Bulk RNA sequencing data from this study and the previous study http://www.brainrnaseq.org/.

Supplementary Fig.10 Zhang et.al

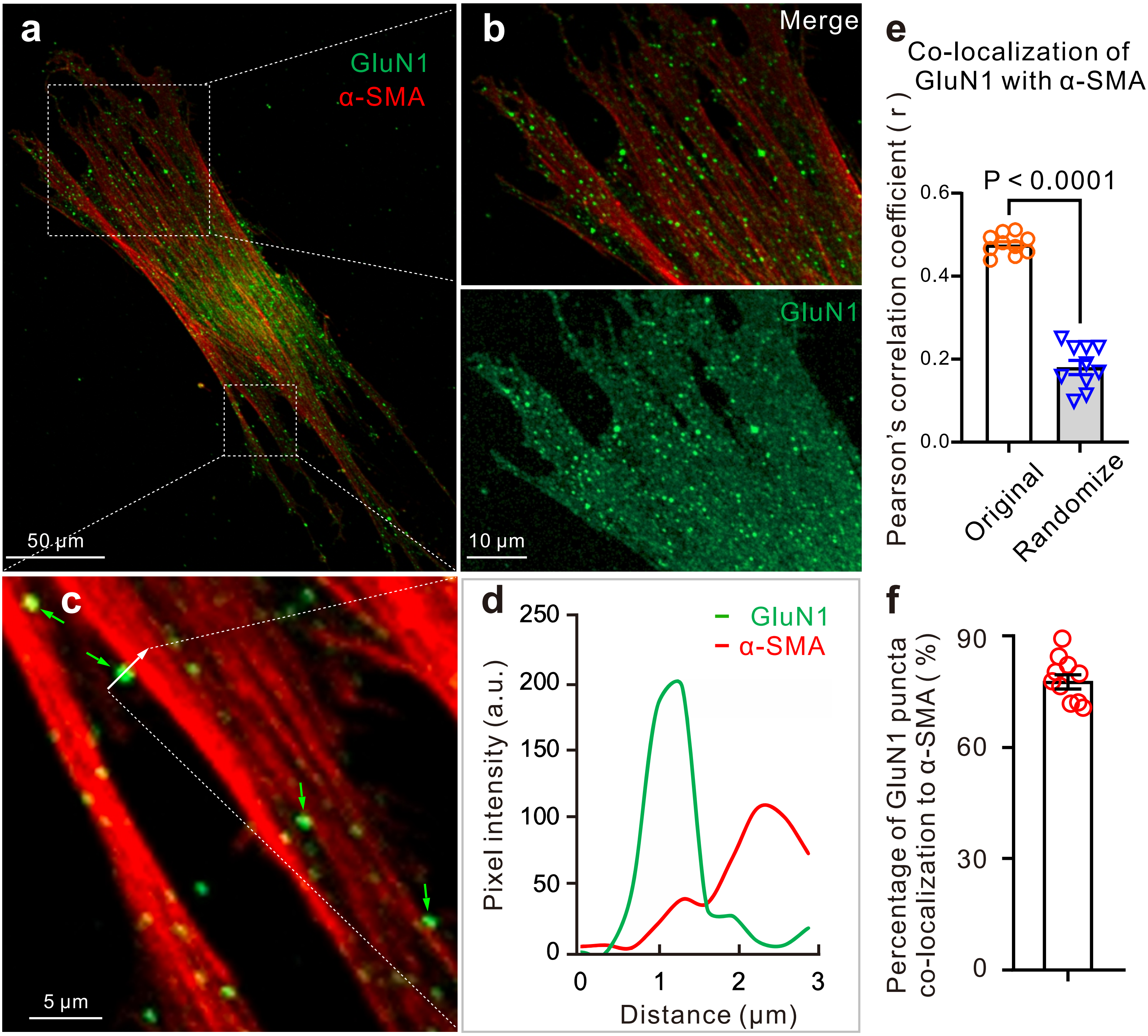

Supplementary Fig. 10 GluN1 puncta are adjacent to αSMA filament in primary pial aSMCs. (**a**) Representative immunofluorescent images of the subcellular relationship of GluN1 with **α**-SMA. (**b**) Zoomed-in image of the region in the upper box in **a**. (**c**) Zoomed-in image of the region in the bottom box in **a**. GluN1 puncta closely associated with α-SMA filaments are pointed by green arrows. (**d**) The line histogram of two proteins pixel intensity along the white arrow in **c**. (**e**) Co-localization analysis of GluN1 with **α**-SMA. (**f**) Quantified distribution of the GluN1 puncta colocalized to **α**-SMA versus total puncta number (n = 10 cells). Data are mean ± s.e.m.; nested, unpaired, two-tailed *t*-test (**e**).

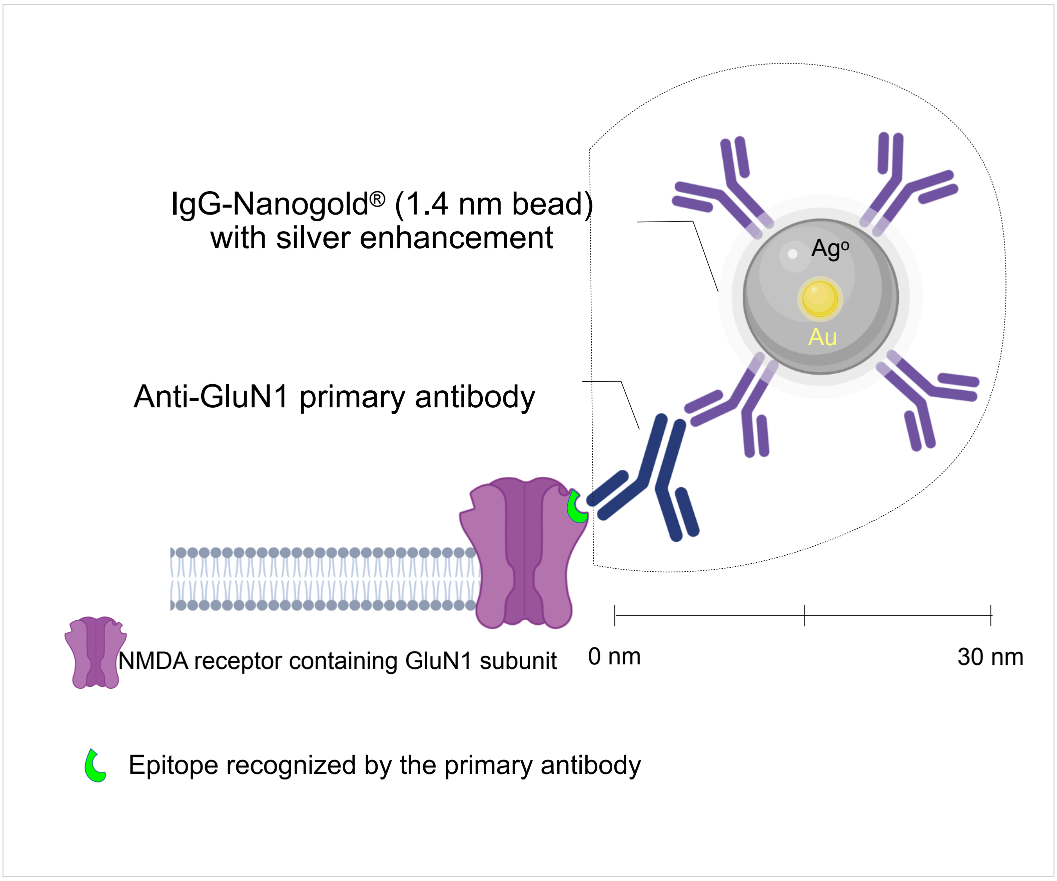
IgG-Nanogold® (1.4 nm bead)
with silver enhancement
Ago
Au
Anti-GluN1 primary antibody
NMDA receptor containing GluN1 subunit
0 nm
30 nm
Epitope recognized by the primary antibody

Supplementary Fig. 11 Illustrative representation of the approximate size scale of immunogold complexes relative to the GluN1 cognate target.

The sketch was created with BioRender.com.

Supplementary Fig.12 Zhang et. al

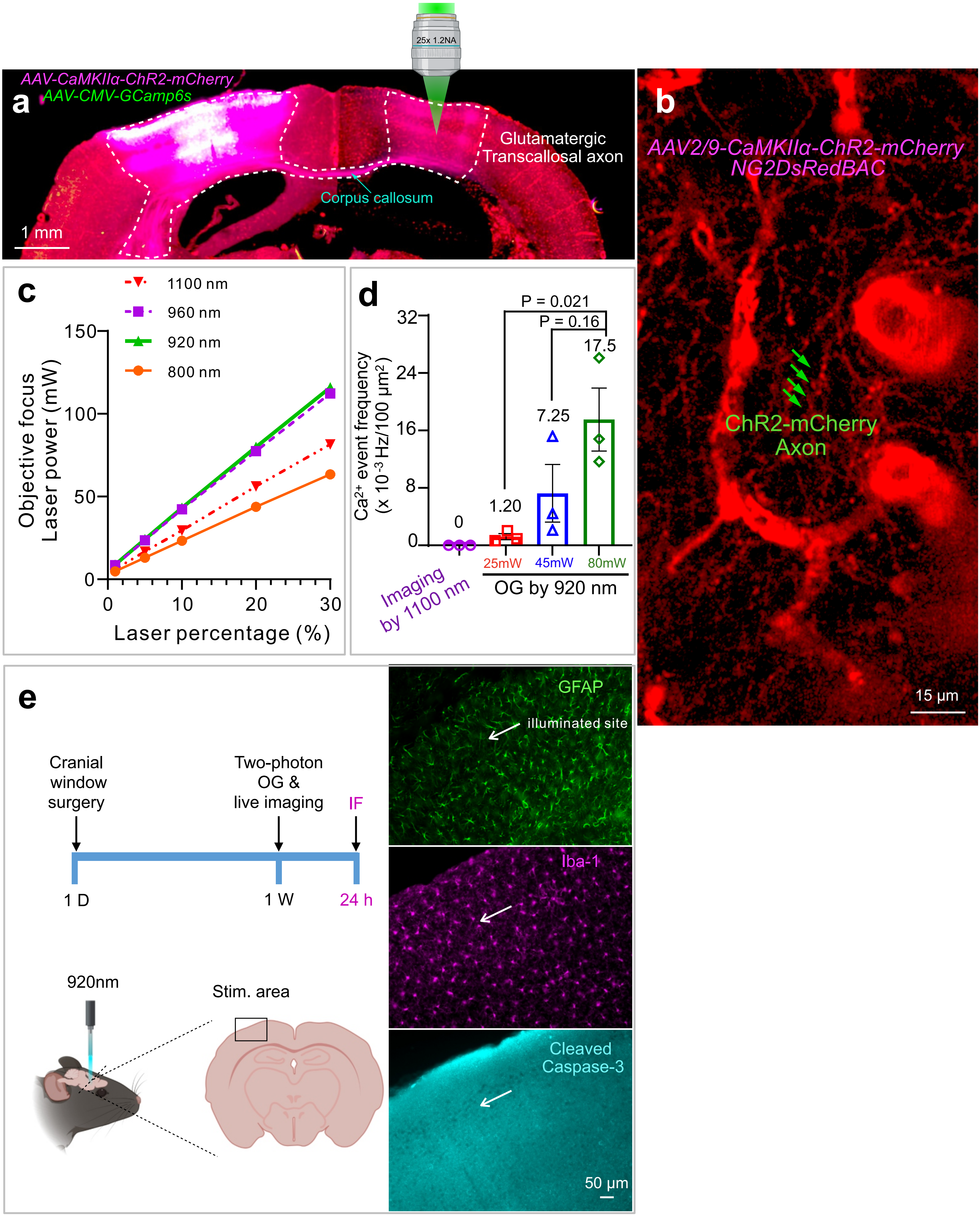

Supplementary Fig. 12 Effective Two-photon optogenetics did not cause brain injury. (**a**) Representation of optogenetics and imaging strategy. A brain section from *NG2DsRed* mouse injected two viruses (*AAV2/9-CaMKIIα-ChR2-mCherry and AAV2/9-CMV-GCamp6s*) on one side and a cranial window was created on the other side. (**b**) A representative live image of the ChR2-mCherry positive axon terminals in the contralateral hemisphere was scanned at the 1100-nm laser wavelength which is optimal to image the mCherry signal while not activating ChR2, N = 6 mice. (**c**) Correlation plot of the measured laser beam powers of varied wavelengths at the focal plane versus the power output settings (%) in the operating software. (**d**) Quantification of Ca^{2+} events observed in the axonal terminals upon an 1100-nm laser scanning and the increasing laser powers at 920 nm. N = 3 mice, data are mean ± s.e.m.; statistical tests were determined by a one-way ANOVA with a post hoc Bonferroni multiple comparison adjustment. (**e**) Upper left: diagram of heat injury detection experiment. Bottom left: immunolabeled sections of mouse brain illuminated at 80 mW, 920 nm for continuous 5 minutes, fixed 24 hours after two-photon stimulation. The schematic was created with BioRender.com. High magnification of the illuminated area is shown in the right panel, N = 3 mice.

Supplementary Fig.13

Zhang et. al

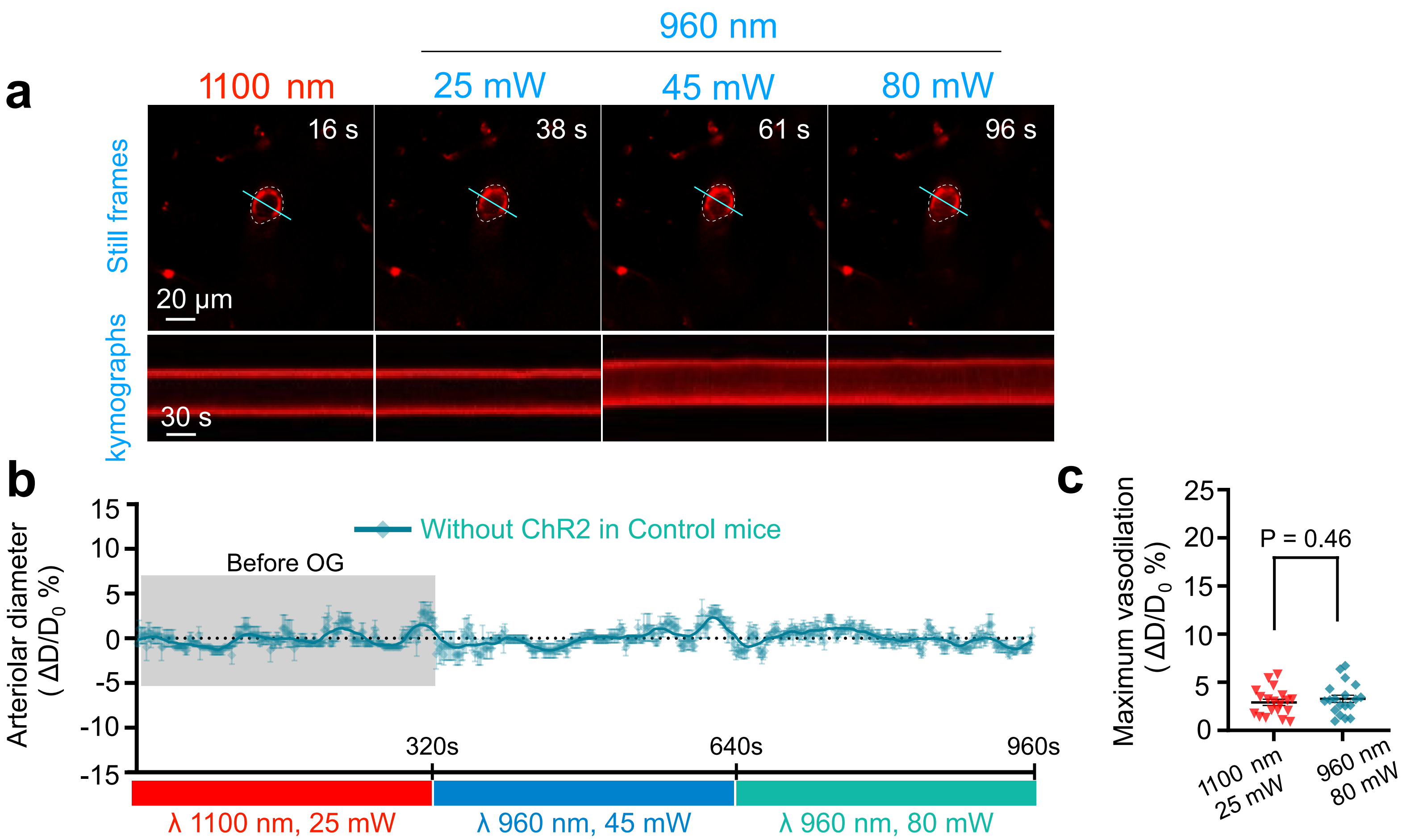

Supplementary Fig. 13 No detectable arteriolar diameter changes in control mice that did not express ChR2 in response to two-photon illuminations.

(**a**) Time-lapse images of a penetrating arteriole in *NG2DsRed* mouse at the focal plane 100.5 μm below pia, scanned by 1100 nm (before OG) and 960 nm at 25, 45, and 80 mW. Bottom, Kymographs for the re-slicing of the time-course images. (**b**) Time course of delta D/D_0 (Diameter changes in **a**) when illuminated at 1100 nm and 960 nm at different powers. (**c**) Maximum vasodilation quantification for **a** and **b**. N = 3 mice, 5 arterioles. Data are mean ± s.e.m.; nested, unpaired, two-tailed *t*-test.

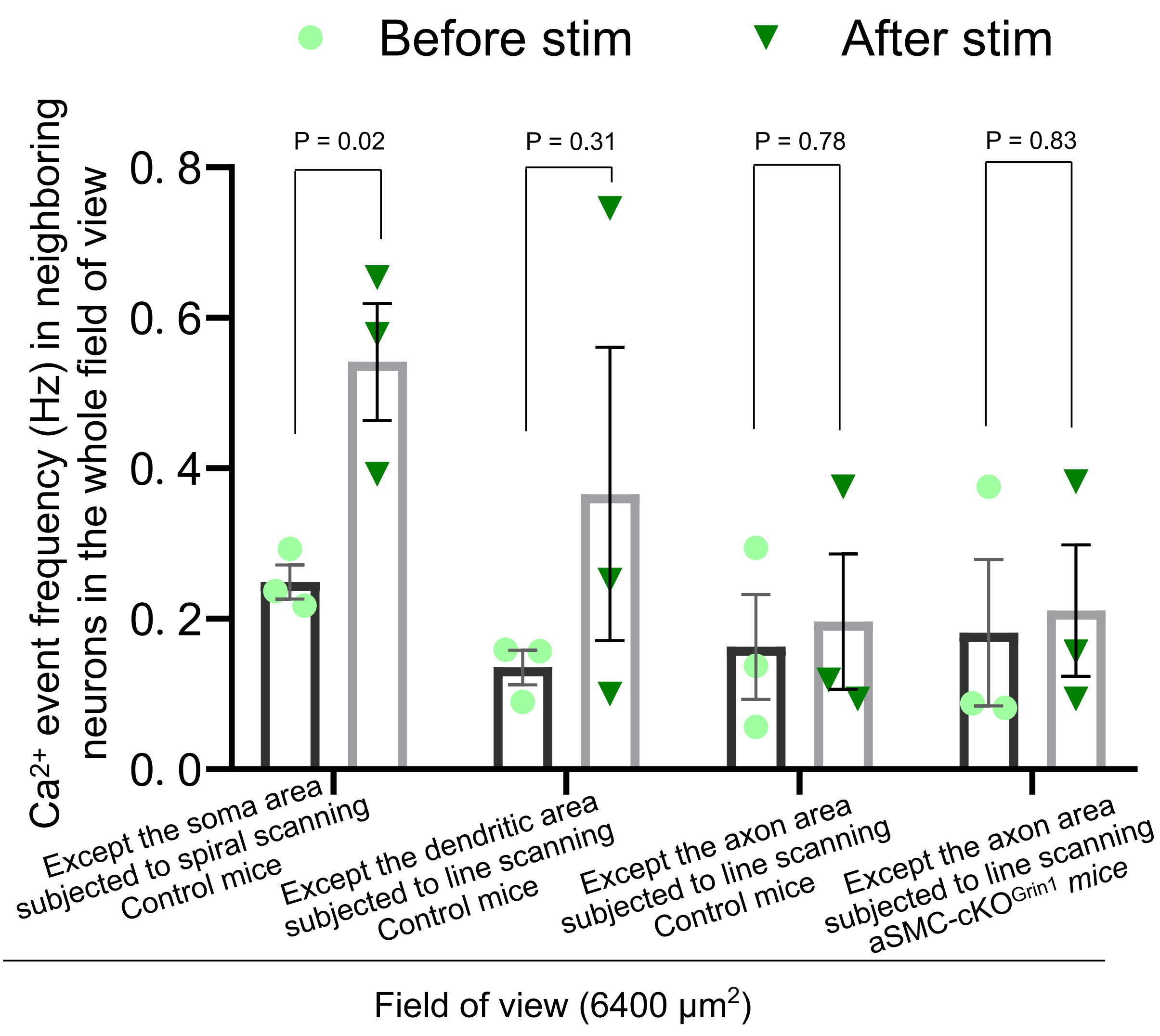

Before stim
After stim
P = 0.02
P = 0.31
P = 0.78
P = 0.83
0. 8
0. 6
0. 4
0. 2
0. 0
Ca2+ event frequency (Hz) in neighboring neurons in the whole field of view
Except the soma area subjected to spiral scanning Control mice
Except the dendritic area subjected to line scanning Control mice
Except the axon area subjected to line scanning Control mice
Except the axon area subjected to line scanning aSMC-cKOGrin1 mice
Field of view (6400 μm2)

Supplementary Fig. 14 Two-photon line-optogenetics on axons and dendrites did not cause neighboring neuron activation, but soma did. Neural Ca^{2+} event frequency in the whole field of views, excluding the events that occurred in the line-scanned areas, before and after the 500-ms high power photostimulation, comparing light green dots to dark green dots. Each view size is 6400 μm^2. N = 3 mice, n = 3 field of views per group. Data are presented in mean ± s.e.m.; nested, unpaired, two-tailed *t*-test.

Supplementary Fig. 15

Zhang et.al

a

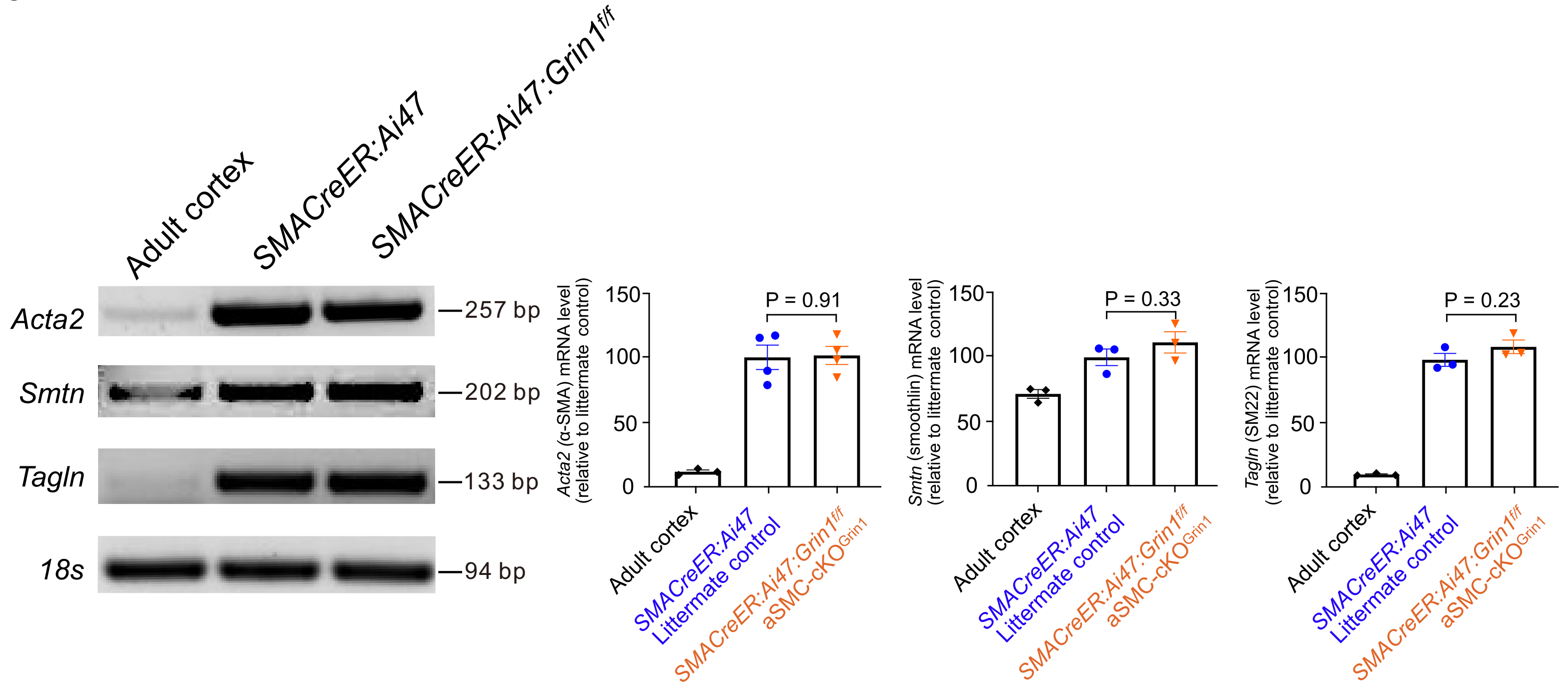

b

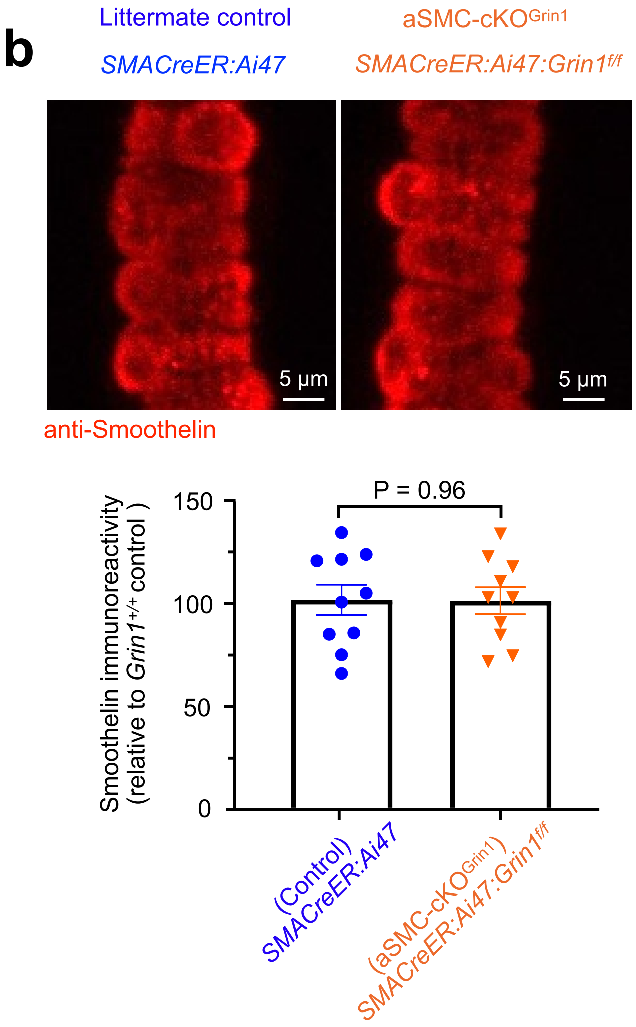

c

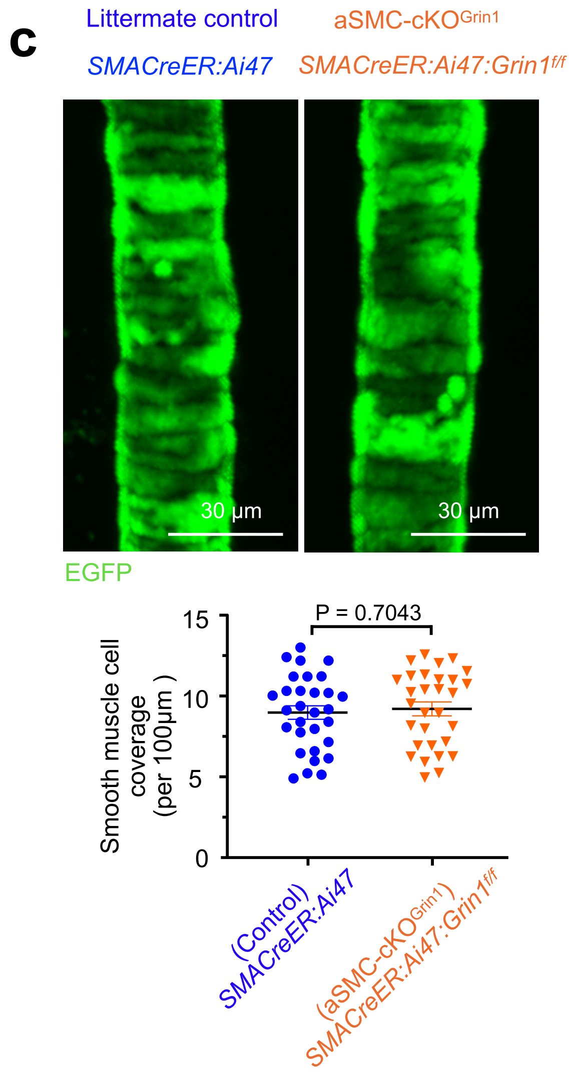

Supplementary Fig. 15 Comparable contractile proteins expression levels and the aSMC coverage on penetrating arterioles. (**a**) RT-PCR for SMC contractile proteins' mRNA expression, including *Acta2*, *Smtn*, *Tagln* in the sorted cells from littermate control (*SMACreER:Ai47*), and *aSMC-cKO*^{Grin1} (*SMACreER:Ai47:Grin1*$^{f/f}$) mice whose aSMCs were labeled by EGFP, and adult mouse cerebral cortices (Left). Four replicates were performed for *Acta2*, and three replicates were performed for *Smtn* and *Tagln* independently (Right). (**b**) Immunostaining of SMC contractile protein smoothelin in the penetrating arterioles from mice of both genotypes. N = 3 mice, n = 10 arterioles. (**c**) Two-photon live images of penetrating arterioles, of which aSMCs were labeled green by including Ai47 reporter into both littermate control and *aSMC-cKO*^{Grin1} mice (upper). Quantification of coverage was calculated by counting aSMC cell number per 100 µm arteriole in length. N = 3 mice, n = 30 arterioles per group. Tamoxifen was given to adult mice (2-3 months old) one month before animal sacrifice in panels **b** and **c**. Data are mean ± s.e.m.; statistical tests were determined by a one-way ANOVA with a post hoc Bonferroni multiple comparison adjustments for **a**, a two-tailed *t*-test for **b** and **c**.

Supplementary Fig. 16

Zhang et.al

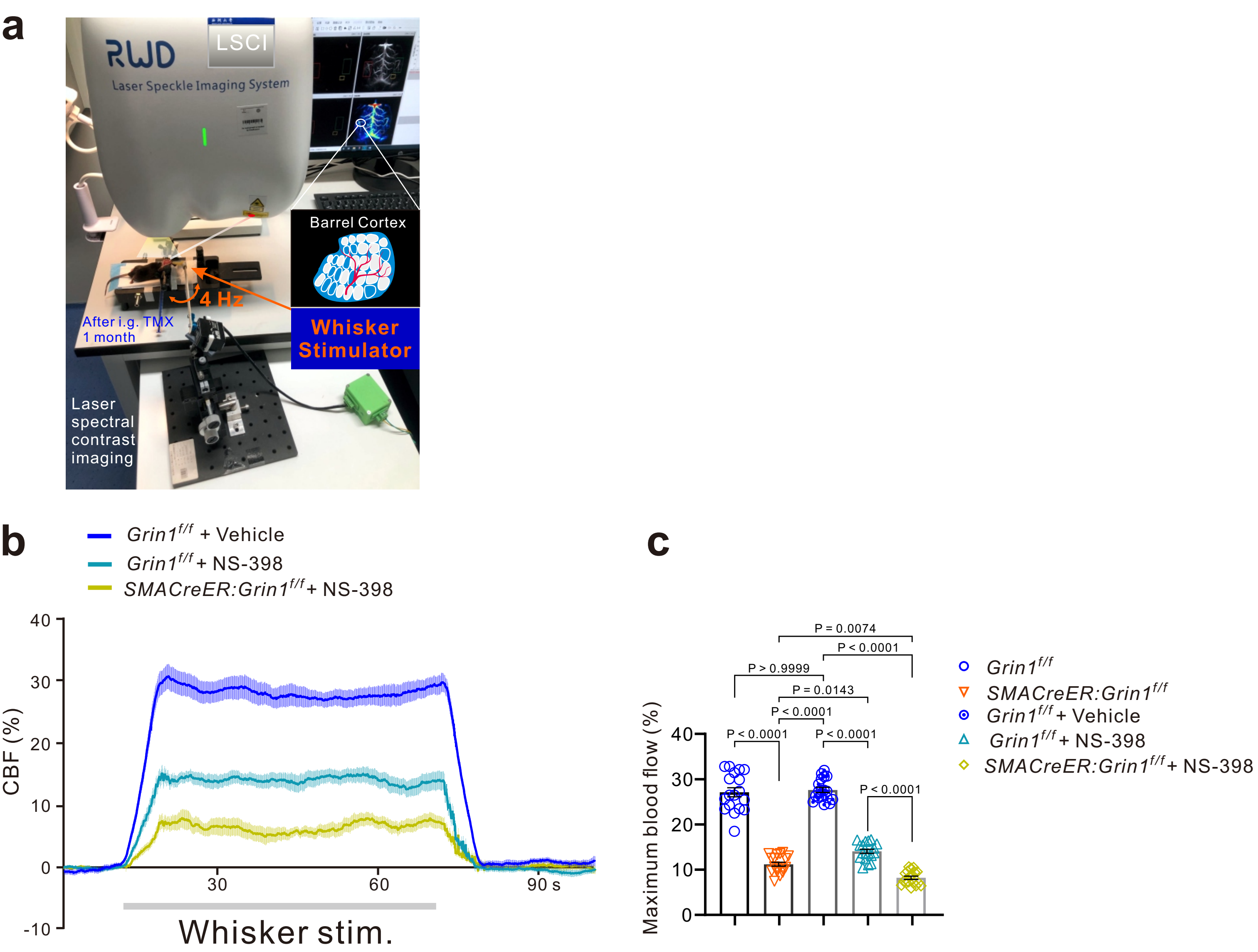

Supplementary Fig. 16 Glu-NsMJ transmission mediates neurovascular coupling independently of postsusynaptic the COX2-PGE2 pathway. (**a**) Schematic showing whisker stimulation and CBF recording setups. Time course (**b**) and maximum percentage (**c**) of CBF changes in the barrel cortex from littermate control (*Grin1$^{f/f}$*) and aSMC-cKO^{Grin1} (*SMACreER:Grin1$^{f/f}$*) upon stimulations. Mice in both genotypes were injected with vehicle or COX2 inhibitor (NS-398). N = 6 mice per group, 3 stimulations for each mouse. The horizontal gray line shows the duration of whisker stimulation. The results of *Grin1$^{f/f}$* (the first bar) and *SMACreER:Grin1$^{f/f}$* (the second bar) mice shared the same data as in Fig. 6p. Data are mean ± s.e.m.; one-way ANOVA with a post hoc Bonferroni multiple comparison adjustment.

Supplementary Fig. 17

Zhang et.al

Fig. 3b

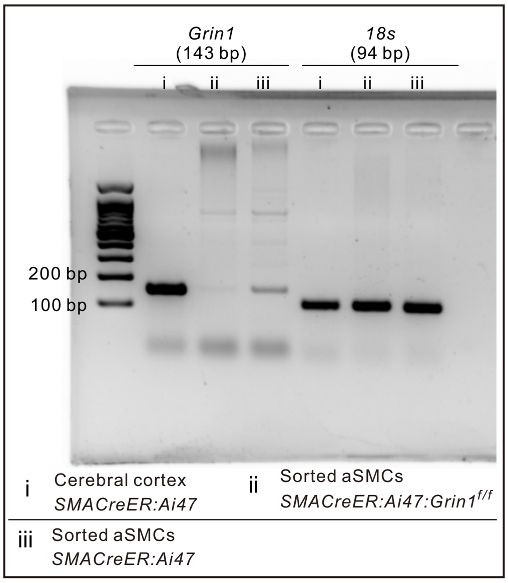

Fig. 3c

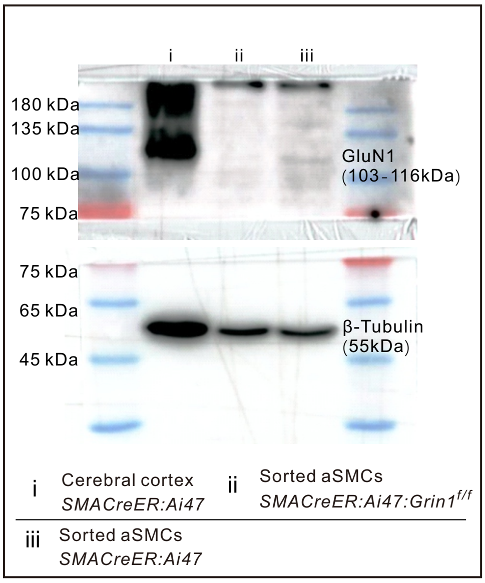

Fig. 6d

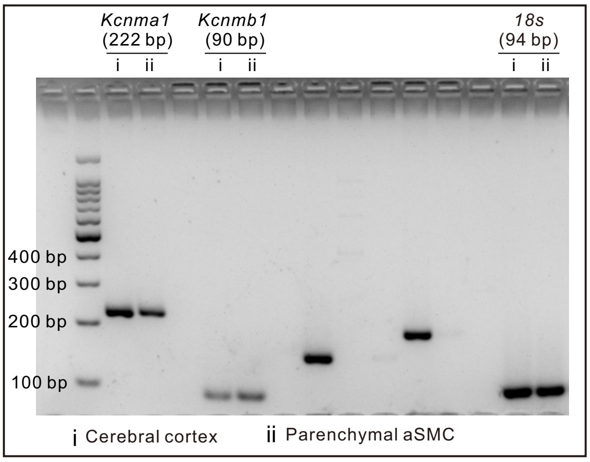

Extended data Fig. 5d

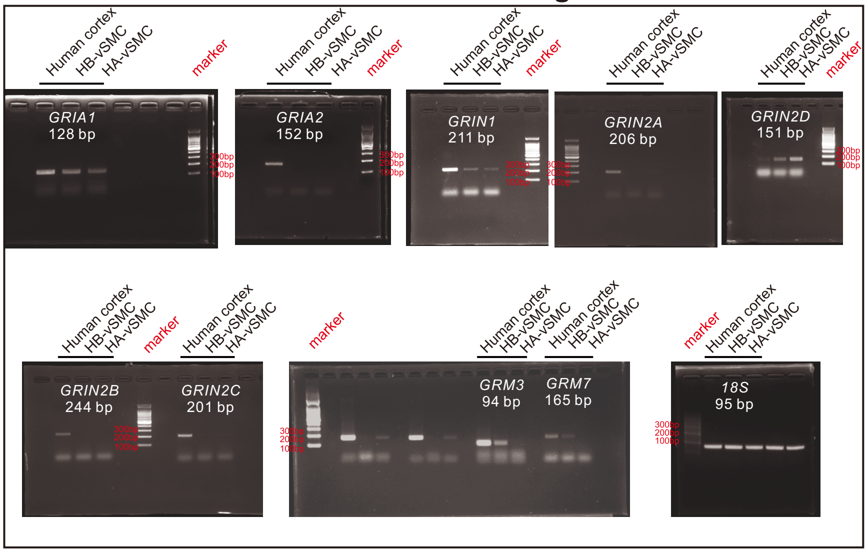

Extended data Fig. 2n

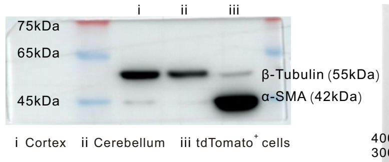

Supplementary Fig. 7d

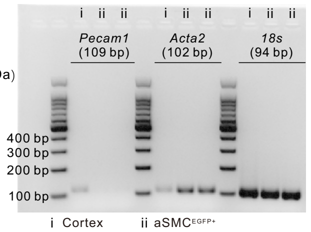

Extended data Fig. 5e

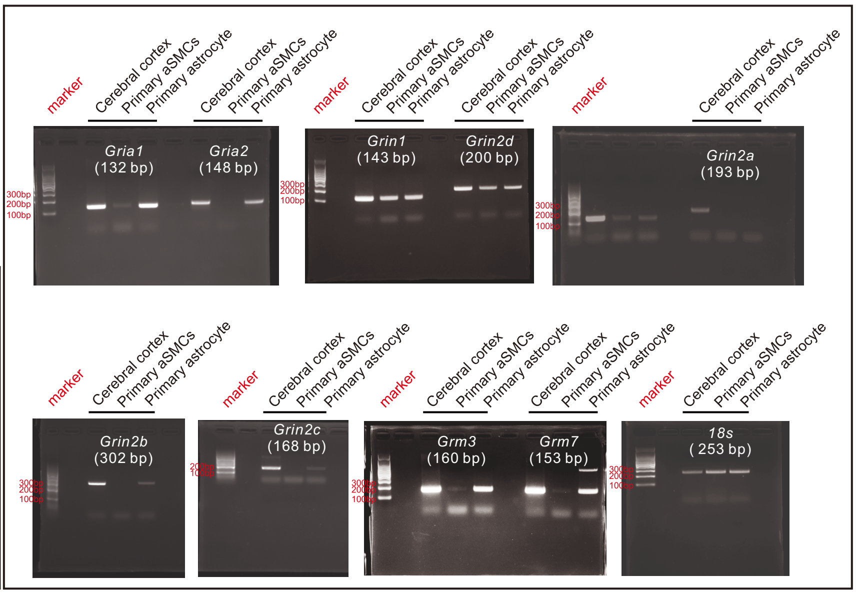

Supplementary Fig. 8a

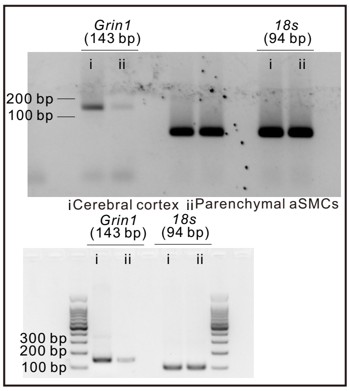

Supplementary Fig. 8c

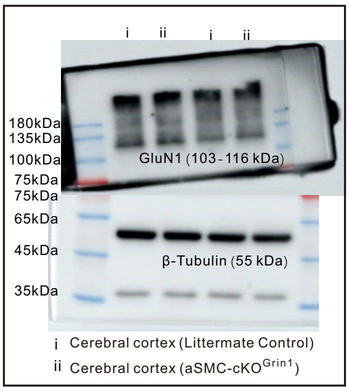

Supplementary Fig. 15a

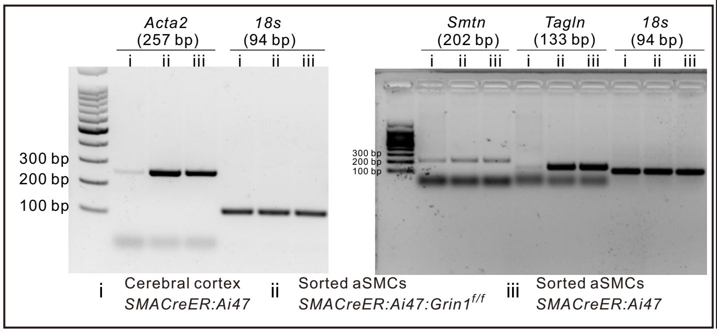

Supplementary Fig. 17 The uncropped scans of all blots and gels in RT-PCR and WB are presented in the main, extended data, and supplementary figures.
