# Supplementary material for: Synaptic-like transmission between neural axons and arteriolar smooth muscle cells drives cerebral neurovascular coupling
Source: Nat Neurosci. 2024 Jan 2;27(2):232–48. doi: 10.1038/s41593-023-01515-0 (PMC10849963; doi:10.1038/s41593-023-01515-0)
Supplement: Supplementary file 2 — Reporting Summary [file 41593_2023_1515_MOESM2_ESM.pdf]

nature portfolio

Corresponding author(s): Jie-Min Jia

Last updated by author(s): Oct 1, 2023

Reporting Summary

Nature Portfolio wishes to improve the reproducibility of the work that we publish. This form provides structure for consistency and transparency in reporting. For further information on Nature Portfolio policies, see our Editorial Policies and the Editorial Policy Checklist.

Statistics

For all statistical analyses, confirm that the following items are present in the figure legend, table legend, main text, or Methods section.

n/a	Confirmed	
☐	☒	The exact sample size (*n*) for each experimental group/condition, given as a discrete number and unit of measurement
☐	☒	A statement on whether measurements were taken from distinct samples or whether the same sample was measured repeatedly
☐	☒	The statistical test(s) used AND whether they are one- or two-sided<br>*Only common tests should be described solely by name; describe more complex techniques in the Methods section.*
☒	☐	A description of all covariates tested
☐	☒	A description of any assumptions or corrections, such as tests of normality and adjustment for multiple comparisons
☐	☒	A full description of the statistical parameters including central tendency (e.g. means) or other basic estimates (e.g. regression coefficient) AND variation (e.g. standard deviation) or associated estimates of uncertainty (e.g. confidence intervals)
☐	☒	For null hypothesis testing, the test statistic (e.g. *F*, *t*, *r*) with confidence intervals, effect sizes, degrees of freedom and *P* value noted<br>*Give P values as exact values whenever suitable.*
☒	☐	For Bayesian analysis, information on the choice of priors and Markov chain Monte Carlo settings
☒	☐	For hierarchical and complex designs, identification of the appropriate level for tests and full reporting of outcomes
☐	☒	Estimates of effect sizes (e.g. Cohen's *d*, Pearson's *r*), indicating how they were calculated

Our web collection on statistics for biologists contains articles on many of the points above.

Software and code

Policy information about availability of computer code

Data collection

The data of whole cerebral arterioles network after tissue clearing were imaged by a light sheet microscope (Carl Zeiss Z.1) with 5X objective (detection optics, NA = 0.16) and Clr Plan-Neofluar 20X objective (clearing immersion, NA = 1.0, ND = 1.45), and images were acquired by Zeiss ZEN software (ver. 3.6, Z.1 Multiview Processing, 3D VisArt) using sCMOS sensor aligned on a special C-mount for optimized image alignment on dual camera port in pixel size 6.5 µm, 15 bit depth and 30fps at 1000 x 1000 pixel in continuous z-drive mode. The serial SEM images of ultrastructure of cerebral penetrating arterioles were collected with the serial block-face SEM microscope (Carl Zeiss, 3view, GeminiSEM 300) with the Gatan DigitalMicrograph software (version 3.5) using a OnPoint BSE detector at 2 kV voltage and 1.8E-03 mBar in high-vacuum mode with focus charge compensation (FCC). The SEM images of co-culture of neuron and aSMCs were collected by Zeiss GeminiSEM 550 with SmartSEM software (version 05.06). The transmission electron microscopy (TEM) images of co-culture of neuron and aSMCs, and the immunogold staining of co-culture were acquired by Thermo Scientific Talos L120C G2 using Ceta S 16M CMOS sensor (4k x 4k) and digital search-and-view camera (SmartCam) and high contrast C-TWIN objective lens with the Velox software (ver. 3.2) at 80 kV and 0.37 nm point resolution and 0.204 line resolution and 40fps. The Z-stack fluorescent images of the same sets of penetrating arterioles in vivo were acquired by the two-photon microscopy (Olympus FLUOVIEW, FVMPE-RS) with InSight X3 laser (Spectra-Physics, wavelength at 680 nm-1300 nm) equipped with Olympus FV31S-SW and FV31S-DT software (ver. 2.6) at high resolution galvanometer scanner and high speed resonance scanning (up to 438 fps) in GaAsP detector and FV30-FGR filter cube for CH1/CH2 detector and FV30-FVG filter cube for CH3/CH4 detector. The X-ray volumetric image was collected by an X-ray microscope (XRM, Xradia 520 Versa, Carl Zeiss) with LabDCT software (ver. 3.1). The X-ray images were superimposed with the two-photon z-stack image by using dragonfly software (ORS, Montreal, version 2022.1). Olympus Motorized Fluorescence Microscope IX83 with CellSens software (version V1.18) was used to time-lapse living image the neuron and aSMC co-cultures applying 20 x objective lens (LUCPlanFLN, NA = 0.45, Ph1, 0-2mm Correction Collar.Phase). Spinning-disk confocal microscope (Nikon, CSU-W1) with Nikon Elements software (NIS ElementsAR ver. 4.6.0) was used to image CTB-488 transportation from aSMC to neuronal soma and calcium events change in aSMCs in vitro. The immunofluorescent images of culture and isolated penetrating arterioles in RNAScope and GluN1, PSD95, and vGlut1 staining were collected by Laser scanning confocal microscope (Zeiss LSM800 with Airyscan) with

Zeiss Zen software (blue edition, ver. 3.6) using Axiocam 702 mono camera. The calcium change of neuron and arteriolar diameter change upon optogenetics stimulation and stroke were also acquire by two-photon microscopy (Olympus FLUOVIEW, FVMPE-RS) with FV31S-SW and FV31S-DT software (ver. 2.6). The blood flow change in barrel cortex after whisker stimulation in control and cKO mice were collected by laser speckle contrast imaging (RFLSI III, RWD) using LSCI software (ver. v01.00.05). The whole brain images of immunofluorescent staining of Iba-1, GFAP and cleaved Caspase-3, and bright field images of brain atrophy section slices were acquired by Fluorescence stereo Zoom microscope (Zeiss Axio Zoom. V16) using Zeiss ZEN software (blue edition, version 3.6). The immunofluorescent images of CaMKIIα, GFAP, Iba-1, CD31, Collagen I, PDGFRα, Smoothelin and CD13 in cultured primary aSMCs or brain slices were acquired by Laser scanning confocal microscope (Zeiss LSM900 with Airyscan), and analyzed by Zeiss Zen software (blue edition, ver. 3.8). Fluorescence-activated cell sorting of primary aSMCs were applied by BD Fusion FACSAria III flow cytometer (BD Biosciences, FCCF-FDF) or MA900 sorter (Sony, FC-MAR). Whole-cell recordings of primary aSMCs were obtained by an Axopatch 700B amplifier (Axon Instruments, Foster City, CA), and the signals were digitized through a Digidata-1550 interface (Axon Instruments). For rotating beam test experiments, mouse travelings along a beam rotating at 3 rpm, were recorded with a high-definition camera (Logitech, C310 HD WEBCAM, 720p/30fps).

Data analysis

Serial stack SEM images were auto-aligned to registration by Amira software (Thermo Fisher, version 2022.1), in which the nonlocal meaning and membrane enhancement algorithms were used to filter the mismatched regions in serial images. Astrocytic end-feet, penetrating arterioles, and neuronal axons and dendrites were manually- and auto- segmented via Imaris software (Oxford, Bitplane, version 9.9). For calculating astrocytic end-feet discontinuity rate, measurements of the rendering volume, meshing surface area, and skeleton length of vessels were conducted by surface-surface contact algorithm in Imaris (version 9.9). NsMJ numbers, NsMJ bouton sizes, junctional clefts and prejunctional vesicle sizes were counted or measured manually by using Gatan DigitalMicrograph software (version 3.5). aSMC microvilli contacting axon, and thickness of basal membrane of cultured aSMCs were measured by Image J2 (Fiji, version 2.5.0). The quantifications of calcium spark-like and calcium wave in aSMCs in vitro were performed by using Nikon Elements software (NIS ElementsAR ver. 4.6.0) and Imaris software (version 9.9). The MATLAB (version 2019a) code of AQuA (https://github.com/yu-lab-vt/AQuA) from open source in GitHub was applied to analyze calcium event frequency in neuron in vivo. The calcium changes in aSMCs after stroke and blood flow volume were quantified by the plugin of Time series analyzer (version 3.0) in Image J (version 1.53) software. The arteriole diameter changes before and after optogenetic stimulations, or during stroke condition were analyzed using custom code performed by MATLAB (https://github.com/JialabEleven/new_vessel_diameter_detection) uploaded in GitHub. The delta fluorescent changes of calcium in vitro were visualized by custom code performed by MATLAB (https://github.com/JialabEleven/calcium_spark_adjustment) uploaded in GitHub. The dynamic distances between neurite tips and SMC cell membrane in living co-cultures, the fluorescent intensity in immunofluorecent stainings, the gray values of Western blottings, RT-PCR bands, and postjunctional membranes and neighboring membranes in EM , and punctum counts in immunogold staining were measured by Image J2 (Fiji, version 2.5.0). The R (version 4.2.1) code of ClusterProfile package (version 4.8.3) from open source (https://www.bioconductor.org/packages/release/bioc/html/clusterProfiler.html) were exploited for identifying differentially expressed genes and RStudio (version 2022.02.2+485) was used for GO and KEGG enrichment analyses of RNA-Sequencing. Fluorescence-activated cell sorting of primary aSMCs were analyzed by FlowJo software (ver. 10.6.2). Data acquisition and analysis of patch-clamp whole-cell recordings for cultured aSMCs were performed by pClamp (Axon Instruments, version 11.2). The co-localization analyses for GluN1 with PSD-95, Kcnmb1, α-SMA, or vGluT1 respectively were conducted by Image J (version 1.53) software with the plugin of colocalization test (https://imagej.net/imaging/colocalization-analysis). The fall frequency, travel distance and average traveling speed of mice in rotating beam tests were analyzed by EthoVision XT software (Netherlands, Noldus Information Technology bv, version 15.0.1416). All Data were analyzed and presented with GraphPad Prism (version 9.5.0).

For manuscripts utilizing custom algorithms or software that are central to the research but not yet described in published literature, software must be made available to editors and reviewers. We strongly encourage code deposition in a community repository (e.g. GitHub). See the Nature Portfolio guidelines for submitting code & software for further information.

Data

Policy information about availability of data

All manuscripts must include a data availability statement. This statement should provide the following information, where applicable:

- Accession codes, unique identifiers, or web links for publicly available datasets
- A description of any restrictions on data availability
- For clinical datasets or third party data, please ensure that the statement adheres to our policy

All data generated or analyzed in the current study are included in this published article and its supplementary information file. Source data (N = 1-16) are provided in this paper. The raw RNA-seq data for parenchymal aSMCs (6 samples: GSM7493520-GSM7493525) and cerebral cortex (6 samples: GSM6568826-GSM6568831) conducted by this study have been uploaded to the NCBI GEO database under the accession number GSE213026. The following publicly available datasets were used: Single-cell RNA-seq database of mouse brain vasculature from Betsholtz lab (Michael Vanlandewijck, et al. Nature, 2018, DOI: 10.1038/nature25739) with the website (https://betsholtzlab.org/VascularSingleCells/database.html) and NCBI GEO database labeled as GSE98816. Bulk RNA-seq data of brain cells from Zhang et al. (Zhang, Y. et al. The Journal of Neuroscience, 2014, DOI: 10.1523/JNEUROSCI.1860-14.2014) with website (http://www.brainrnaseq.org/) and deposited in NCBI GEO database under the accession number GSE52564.

Human research participants

Policy information about studies involving human research participants and Sex and Gender in Research.

Reporting on sex and gender

One female donor, however, sex and gender are not considered in this study design.

Population characteristics

One biospecimen of Asian was used in this study. The donor, a 91-year-old female with a medical history of heart disease and hypertension, gifted the tissue to further scientific research.

Recruitment

The postmortem Brodmann brain area tissue was obtained from a generous adult donor through the Shanghai Red Cross. All tissue samples were donated with full, informed consent. Samples were selected based on the availability of the tissues, which is unlikely to have impacted the results and conclusions.

Ethics oversight	All acquire process is under a protocol approved by Huashan Hospital of Fudan University with approval from local ethics committees and patient consent. All procedures also approved by the Institutional Ethics Committee at the School of Life Sciences, Westlake University.

Note that full information on the approval of the study protocol must also be provided in the manuscript.

Field-specific reporting

Please select the one below that is the best fit for your research. If you are not sure, read the appropriate sections before making your selection.

☒ Life sciences ☐ Behavioural & social sciences ☐ Ecological, evolutionary & environmental sciences

For a reference copy of the document with all sections, see nature.com/documents/nr-reporting-summary-flat.pdf

Life sciences study design

All studies must disclose on these points even when the disclosure is negative.

Sample size	No statistical methods were used to predetermine sample sizes, but our sample sizes are similar to those reported in previous publications (Zhang, Y. et al. The Journal of Neuroscience, 2014, DOI: 10.1523/JNEUROSCI.1860-14.2014; Stephanie Holstein-Rønsbo. et al. Nature Neuroscience, 2023, DOI: 10.1038/s41593-023-01327-2). For experiments involving the ultrastructure characterization of neurovascular unit, n = 3 penetrating arterioles (p-arterioles) were chosen as the minimal replicate number. Because of the special challenge of preparing 3view SEM samples, as long as the structure remains intact, three p-arterioles in the brain of a single mouse could represent the normal structural characteristics. For the statistical analysis of the coverage of astrocytic endfoot, a 50-micron segment unit of p-arterioles with lengths ranging from 400-500 microns was chosen as a calculation unit, because the endfoot coverage on P-arteriole at the length of 50 micron is sufficient to show the endfoot structural profile. For the Western blot, RT-PCR, RNA-Scope and immunofluorecent staining experiments, n = 3 biological replicates were chosen as the minimal replicate number, which were required to detect unpaired t-test significance with an alpha rate set as 0.05 in a standardly powered experiment. For the calcium events measurements in vitro, the diameter and blood flow volume of P-arteriole change experiments, and whisker stimulation experiments, a minimum of 3 cells, or 3 penetrating arterioles, or 3 mice were chosen. A minimal amount of cells, or arterioles, or mice were required to detect a p-value less than 0.05. For behavioral experiments, and detecting the brain atrophy percentage, more than 6 mice were chosen because it is the percentile data that belong to a binary variable. For the whole-cell recording of primary aSMCs, a minimum of 4 cells were chosen to confirm the conclusion.
Data exclusions	In the two-photon optogenetic experiments, if the calcium of neuronal axon, dendrite or soma were not successfully activated by laser frame or line scanning stimulations, or if the GCamp6 protein was denatured, resulting its intensity remained at a constant brightness, we excluded this data. In the whisker stimulation experiments, five technical trials of cerebral blood flow recording via laser speckle contrast imaging were acquired for each mouse, of which the largest and smallest CBF variation data were excluded, and the remaining three trials were used. To perform stroke models using the MCAO surgery, animals that received failed surgery (i. e. the instant reduction of CBF detected by LSCI was smaller than 80%) were excluded. For those experiments that there were no spare time for LSCI imaging, if no vasoconstriction was observed during the entire 2-hour occlusion period under two-photon microscopy after MCAO surgery, we excluded this mouse, being considered as failed surgery. For behavioral test experiments, measurements of body weight during post-stroke 18 days, once mice died, we ceased the process, excluded the behavior and body weight data, and initiated a separate mouse. For bulk RNA sequencing experiments, all the sorted aSMCs contaminated with ECs were excluded. In the whole-cell recording process, the following standards and parameters are used to select the recorded cells and the electrophysiological signals<br>I.Morphology of cells<br>Integrity: The selected cells should have a complete morphology, with no obvious cracks or damage.<br>Smoothness: The cell surface should be smooth, with no large particles or protrusions.<br>Adhesion: The cells should be tightly adhered to the culture plate or cover slip, loose or floating cells will be excluded.<br>Elasticity: The cells should have a certain toughness and elasticity, they should be able to recover after resisting minor impacts or pressure during the operation.<br>II.Parameters for whole-cell patch clamp recordings<br>Leakage Current: The data is acceptable when the leakage current is less than 50 pA.<br>Membrane Resistance: The membrane resistance of the recorded cells should be greater than 100 MΩ.<br>Series Resistance: Series resistance (< 25 MΩ) was continuously monitored and remained stable to within 20%. Additionally, exclusion occurred due to poor imaging quality.
Replication	Each experiment presented in this study was repeated in multiple separate experiments or multiple animals (between 5-23). All results in the paper are acquired from analyses of multiple repeats and animals. All experiments were replicated in at least three independent cohorts successfully unless stated otherwise.
Randomization	Animals in test and control groups were littermates and selected randomly.
Blinding	The investigator was not blinded to most of the experiments in vitro because the cell and isolated penetrating arterioles experiments were performed using a pipeline applied equally to all conditions and replicates. However, the investigators were blind to the behavioral test experiments and brain atrophy quantification when animals were littermates. All the tests were conducted first, and then the genotype was identified later.

Reporting for specific materials, systems and methods

We require information from authors about some types of materials, experimental systems and methods used in many studies. Here, indicate whether each material, system or method listed is relevant to your study. If you are not sure if a list item applies to your research, read the appropriate section before selecting a response.

Materials & experimental systems

n/a	Involved in the study
☐	☒ Antibodies
☐	☒ Eukaryotic cell lines
☒	☐ Palaeontology and archaeology
☐	☒ Animals and other organisms
☒	☐ Clinical data
☒	☐ Dual use research of concern

Methods

n/a	Involved in the study
☒	☐ ChIP-seq
☐	☒ Flow cytometry
☒	☐ MRI-based neuroimaging

Antibodies

Antibodies used

Anti-GluN1 antibody (Merck Millipore, Catalog no. MAB363, clone 54.1, 1:500),
Anti-Tau antibody (Merck Millipore, Catalog no. MAB3420A4, clone PC1C6, 1:200),
Anti-PSD-95 antibody (Merck Millipore, Catalog no. MAB1598, clone 7E3-1B8, 1:500),
Anti-α-smooth muscle actin antibody (ABclonal Technology, Catalog no. A1011, 1:1000),
Anti-β-Tubulin antibody (ABclonal Technology, Catalog no. AC021, clone AMC0498, 1:5000),
Cy3-conjugated anti-α-smooth muscle actin antibody (Sigma Aldrich, Catalog no. C6198, clone 1A4, 1:200),
FITC--conjugated anti-α-smooth muscle actin antibody (Sigma Aldrich, Catalog no. F3777, clone 1A4, 1:200),
Anti-NeuN antibody (Merck Millipore, Catalog no. ABN90P, clone A60, 1:500),
anti-CaMKIIα antibody (Invitrogen, Catalog no. PA5-19128, 1:500),
anti-GFAP antibody (ThermoFisher Scientific, Catalog no. UC276149, clone 2.2B10, 1:500),
anti-Iba-1 antibody (Wako Pure Chemical Industries, Ltd. Catalog no. 019-19741, 1:500),
anti-CD31 antibody (BD Biosciences, Catalog no. 557355, clone MEC13.3, 1:500),
anti-Collagen I antibody (Abcam, Catalog no. ab34710, 1:500),
anti-PDGFRα antibody (R & D systems, Catalog no. AF1062, 1:500),
anti-Cleaved Caspase-3 antibody (Cell Signal Technology, Ltd. Catalog no. 9661, 1:500),
anti-Smoothelin antibody (Abcam, Catalog no. ab219652, 1:200),
PE-Alexa Fluor 594 conjugated anti-CD31 antibody (Biolegend, Catalog no. 102520, clone MEC13.3, 1:200),
APC conjugated anti-CD45 antibody (Biolegend, Catalog no. 103112, clone 30-F11, 1:200),
anti-CD13 antibody (R & D systems, Catalog no. AF2335, 1:200),
anti-sloβ1(Kcnmb1) antibody (Alomone labs, Catalog no. APC-036, 1:200),
anti-vGluT1 antibody (Synaptic Systems, Catalog no. 135311, clone 317D5, 1:500),
Alexa Fluor 488-conjugated goat anti-mouse IgG antibody (Invitrogen, Catalog no. A-11034, 1:1000).

Validation

Anti-GluN1 antibody (Cat#MAB363): species reactivity, Xenopus, rat, human, monkey; applications for ELISA, immunocytochemistry, immunohistochemistry (formalin-fixed, paraffin-embedded sections), radioimmunoassay, Western blot; description: glutamate is a widely utilized excitatory neurotransmitter in mammalian brains. The ion channels activated by glutamate are typically divided into two classes. Those that are sensitive to N-methyl-D-aspartate (NMDA) are designated NMDA receptors (NMDAR) while those activated by kainate (KA) and a-amino-3-hydroxy-5-methyl-4-isoxalone propionic acid (AMPA) are referred to as non-NMDA receptors. NMDA receptors (NMDARs) encompass different combinations of subunits, GluN1 (NR1), GluN2(NR2) and GluN3(NR3). The GluN1 subunit is expressed in abundance in virtually all regions of the brain; reference: an oral vaccine against NMDAR1 with efficacy in experimental stroke and epilepsy. M J During et al. Science (New York, N.Y.), 287(5457), 1453-1460 (2000-02-26).
Anti-tau antibody (Cat#MAB3420A4): species reactivity, human, mouse, rat; applications for immunocytochemistry and immunohistochemistry; description: tau, a microtubule binding protein which serves to stabilize microtubules in growing axons, is found to be hyperphosphorylated in paired helical filaments (PHF), the major fibrous component of neurofibrillary lesions associated with Alzheimer's disease. Hyperphosphorylation of tau is thought to be the critical event leading to the assembly of PHF. Six tau protein isoforms have been identified, all of which are phosphorylated by glycogen synthase kinase 3 (GSK 3). Cellular and subcellular localization: in situ, anti-tau-1 has a stringent specificity for the axons of neurons. The antibody does not stain the cell bodies or dendrites of neurons, nor does it stain any other cell type (4). However, this in vivo intracellular specificity is not maintained in culture: anti-tau-1 stains the axon, cell bodies, and dendrites of rat hippocampal neurons grown in culture (5). The specificity of anti-tau-1 was originally thought to represent the restricted expression of tau to axons. Later studies revealed that this specificity is dependent on the state of phosphorylation. In dephosphorylated samples (samples treated with alkaline phosphatase) anti-tau-1 stains astrocytes, perineuronal glial cells, and the axons, cell bodies and dendrites of neurons, while in untreated samples, anti-tau-1 stains only axons (6). (The epitope recognized by anti-tau-1 is probably at or near a phosphorylated site.); reference: regulation of axon growth by the JIP1-AKT axis. Dajas-Bailador, F; Bantounas, I; Jones, EV; Whitmarsh, AJ. Journal of Cell Science. 2014 Jan 1;127(Pt 1):230-9. doi: 10.1242/jcs.137208. Epub 2013 Nov 6.

Anti-PSD-95 antibody (Cat#MAB1598): Species reactivity, bovine, rat, mouse; applications for immunocytochemistry, immunoprecipitation and Western blot; description, the postsynaptic density (PSD) is an electron dense structure just beneath the postsynaptic membrane. Several functions have been proposed for the PSD including regulating receptor number and clustering, anchoring signal transduction molecules at the synapse and mediating adhesion between the presynaptic and postsynaptic membranes. The most abundant PSD protein is the [alpha] subunit of the type II calcium/calmodulin dependent protein kinase ([alpha]CaMKII). This protein is likely to play a role in the calcium-mediated signal transduction at the synapse that mediates certain forms of synaptic plasticity. Another major PSD protein is PSD-95, a member of the guanylate kinase family (GUK) of proteins; reference: PSD-95 is post-transcriptionally repressed during early neural development by PTBP1 and PTBP2. Zheng, S; Gray, EE; Chawla, G; Porse, BT; O'Dell, TJ; Black, DL. Nature Neuroscience. 2012 Jan 15;15(3):381-8, S1. doi: 10.1038/nn.3026.

	Cy3-conjugated anti-α-smooth muscle actin antibody (Cat#C6198): species reactivity, human, frog, sheep, chicken, goat, bovine, rat, guinea pig, mouse, canine, rabbit, snake; applications for flow cytometry, immunocytochemistry, immunofluorescence, immunohistochemistry, Western Blot; description: monoclonal anti-actin, α-smooth muscle (mouse IgG2a isotype) is derived from the 1A4 hybridoma produced by the fusion of mouse myeloma cells and splenocytes of immunized BALB/c mice. The antibody (also known as anti-α-Sm-1) is specific for the single isoform of α-smooth muscle actin. It reacts specifically with α-smooth muscle actin in immunoblotting assays and labels smooth muscle cells in frozen or formalin-fixed, paraffin-embedded tissue sections; reference: Endothelial-myofibroblast transition contributes to the early development of diabetic renal interstitial fibrosis in streptozotocin-induced diabetic mice. Jinhua Li et al. The American Journal of Pathology, 175(4), 1380-1388 (2009-09-05). FITC-conjugated anti-α-smooth muscle actin antibody (Cat#F3777): species reactivity, human, frog, sheep, chicken, goat, bovine, rat, guinea pig, mouse, canine, rabbit, snake; applications for flow cytometry, immunocytochemistry, immunofluorescence, immunohistochemistry, Western Blot; reference: cyclooxygenase-2 in endothelial and vascular smooth muscle cells restrains atherogenesis in hyperlipidemic mice. Tang S Y, et al. Circulation, 129(17), 1761-1761 (2014).

Eukaryotic cell lines

Policy information about cell lines and Sex and Gender in Research

Cell line source(s)	HB-vSMC (Human, ScienCell, cat. No 1100)<br>HA-vSMC (Human, ATCC, cat. No CRL-1999)
Authentication	All cell lines have been authenticated by RT-PCR, using the Acta2, Tagln, and Smtn primers for high enrichment to confirm their identity.
Mycoplasma contamination	Cell lines were not tested for mycoplasma contamination because no indication of contamination was observed.
Commonly misidentified lines<br>(See ICLAC register)	No commonly misidentified cell lines were used.

Animals and other research organisms

Policy information about studies involving animals; ARRIVE guidelines recommended for reporting animal research, and Sex and Gender in Research

Laboratory animals	The strains, including NG2DsRedBac, Thy1-YFP-H, Ai14 (Gt(ROSA)26Sor tm14(CAG-tdTomato)Hze), Ai96(Gt(ROSA)26Sor tm96(CAG-GCaMP6s)Hze mice (The Jackson Laboratory, stock Nos: 008241, 003782, 007914, and 024106 respectively), SMACreER (Tg(Sma-CreERT2)12Pcn), Ai47 (Gt(ROSA)26Sor tm47(CAG-EGFP)Hze) mice, Grin1 fl/fl (Grin1 tm2Stl , The Jackson Laboratory, stock no: 005246), PDGFRβCreER (Tg(Pdgfrb-cre/ERT2)6096Rha (The Jackson Laboratory, stock no: 029684), Myh11CreER (Tg(Myh11-cre/ERT2)F31Gko/J (The Jackson Laboratory, stock no: 037658) mice were used in this study. All mouse strains were used for experiments in adult mice aged 2-4 months or neonatal mice aged P0-P4. Standard chow and water were provided to the mice ad libitum. Four mice were housed in each cage in a standard animal room on a 12-h light/dark cycle (lights on at 7 a.m.) at 25°C with 40-60% humidity.
Wild animals	This study did not involve wild animals.
Reporting on sex	Male mice with the aged ranged 2 to 4 months were performed in whisker stimulation and behavior experiments, while other experiments applied female or males.
Field-collected samples	This study did not involve samples collected from the field.
Ethics oversight	All mice procedures complied with the Institutional Animal Care and Use Committee (IACUC) guidelines of the School of Life Sciences, Westlake University (approval no.: 20-033-JJM).

Note that full information on the approval of the study protocol must also be provided in the manuscript.

Flow Cytometry

Plots

Confirm that:

☒ The axis labels state the marker and fluorochrome used (e.g. CD4-FITC).

☒ The axis scales are clearly visible. Include numbers along axes only for bottom left plot of group (a 'group' is an analysis of identical markers).

☒ All plots are contour plots with outliers or pseudocolor plots.

☒ A numerical value for number of cells or percentage (with statistics) is provided.

Methodology

Sample preparation	Parenchymal aSMCs were isolated from neonatal SMACreER:Ai14 mouse brains for in vitro culture experiments. Pregnant SMACre ER:Ai14 mice were intraperitoneally injected or intragastrically administered tamoxifen on an embryonic day (E)18 to induce tdTomato expression in aSMCs. Brain samples were collected on P1, and their leptomeninges were removed, then

single-cell suspensions were obtained using the Neural Tissue Dissociation Kit (P) (Miltenyi Biotec Inc, cat# 130-092-628). In addition, parenchymal aSMCs from brains of adult SMACreER:Ai47 mice were also sorted for the study of profiling molecular expression. Briefly, adult SMACreER:Ai47 mice receive tamoxifen one month before cell sorting. Brain were isolated and digested mildly, then enriched cerebral arterioles initially with minimal inclusion of capillaries. Further, the enriched arterioles were digested thoroughly in collagen I and IV and trypsin to acquire the single-cell suspensions.

Instrument	Parenchymal aSMCs were sorted via a BD FACSAria III flow cytometer (BD Biosciences, USA) or MA900 sorter (Sony, FC-MAR).
Software	All FACS data were analyzed using FlowJo v10.6.2 software (FlowJo, LLC).
Cell population abundance	The sorted tdTomato positive parenchymal aSMCs from neonatal SMACreER:Ai14 mouse brains were cultured during 7-14 DIV. Anti-α-smooth muscle actin antibody was used to verify the abundance of aSMCs. Results show that the positive percentage is about 98%, indicating the efficiency abundance via cell sorting (Extended Data Fig. 4m).<br>The sorted EGFP positive parenchymal aSMCs from adult SMACreER:Ai47 mouse brains were directly extracted mRNA by TRIzol. No-expression of Pecam1 and enriched-expression of Acta2 by RT-PCR verified the abundance of aSMCs without contamination of endothelial cell (Extended Data Fig. 10d-e).
Gating strategy	The gating strategy of flow cytometry for tdTomato positive parenchymal aSMCs from neonatal SMACreER:Ai14 mouse brains was designed as below. Firstly, cells were selected with a very wide gating setting using forward scatter area/side scatter area (FSC-A/SSC-A). Second, based on FSC-A/FSC-H (forward scatter high) and further SSC-A/SSC-H (side scatter high), adherent cells were removed twice from the parental FSC-A/SSC-A gate. Finally, fluorescent events were selected from the non-adherent cells. tdTomato was excited with a 561-nm laser, and its emission was detected with a 582/15 filter. Wild-type C57BL6/J and Ai14 singlet-positive mice lacking fluorescence were used as the negative controls (Extended Data Fig. 4j-k).<br>In addition, the gating strategy for sorted EGFP positive parenchymal aSMCs from adult SMACreER:Ai47 mouse brains were conducted as below. The parental gating of FSC-A/SSC-A excluded cell debris. Then the gating of FSC-A/FSC-H removed adherent cells. The gating of viability dye (Violet 405) was used to eliminate dead cells. Non-adherent and living cells were additionally performed negative selection to exclude CD31 positive endothelial cells and CD45 positive immune cells contaminations (Extended Data Fig. 10a-b).

☒ Tick this box to confirm that a figure exemplifying the gating strategy is provided in the Supplementary Information.
