# Supplementary material for: Author Correction: Synaptic-like transmission between neural axons and arteriolar smooth muscle cells drives cerebral neurovascular coupling
Source: Nat Neurosci. 2026 Sep 25;29(11):2889. doi: 10.1038/s41593-026-02474-y (PMC13724267; doi:10.1038/s41593-026-02474-y)

nature neuroscience

Supplementary information

Author Correction: Synaptic-like transmission between neural axons and arteriolar smooth muscle cells drives cerebral neurovascular coupling

In the format provided by the authors and unedited

Fig. 1

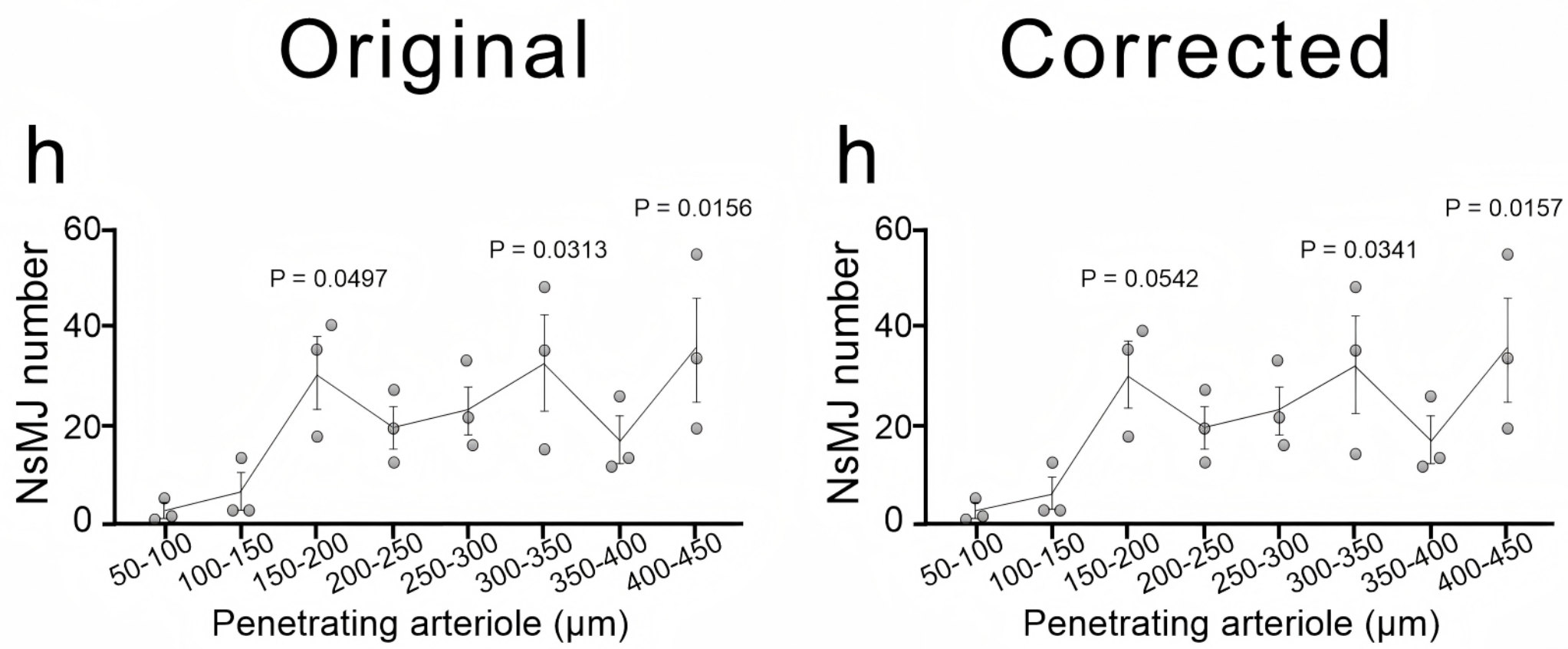

Fig. 1

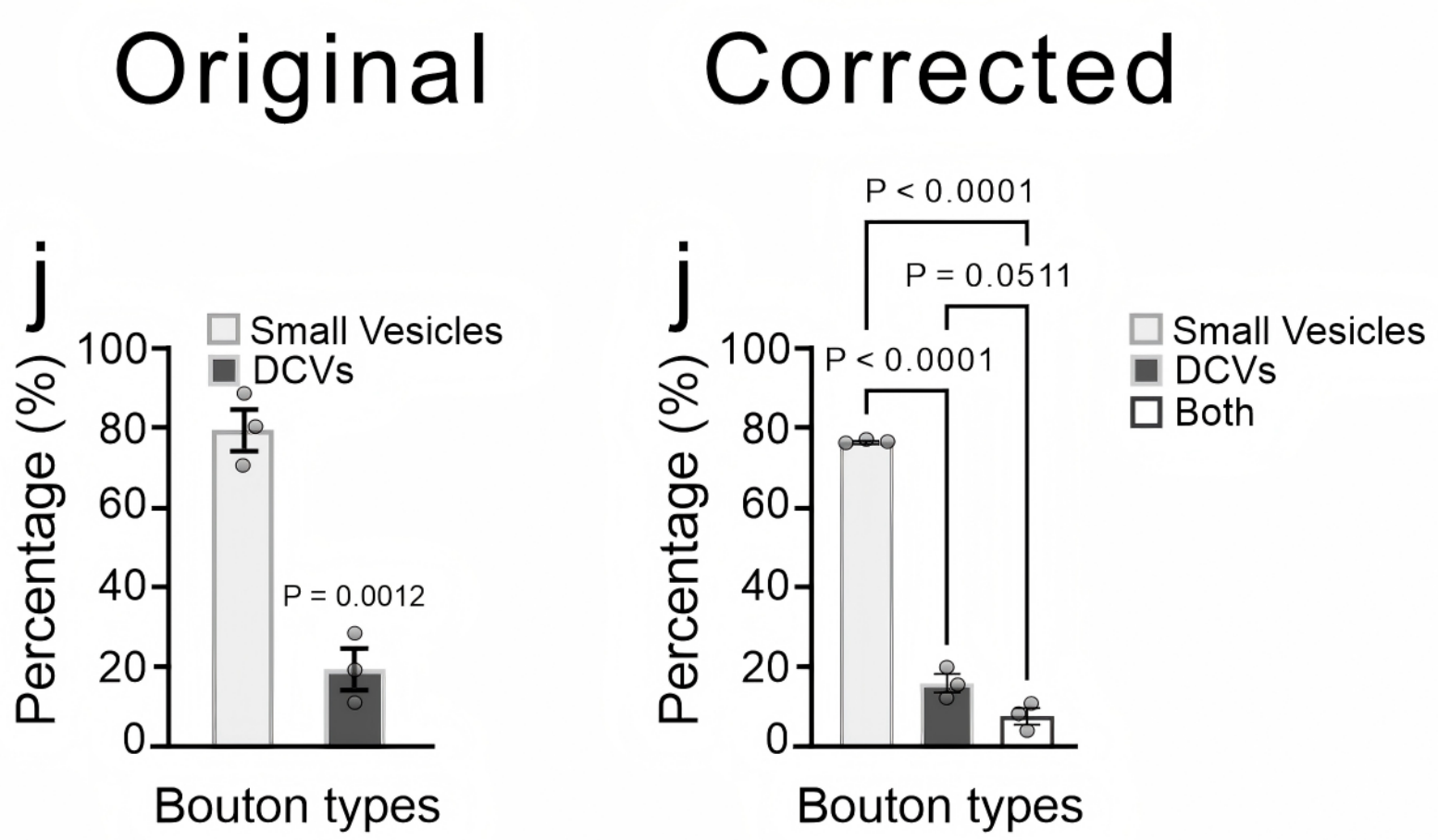

Fig. 1

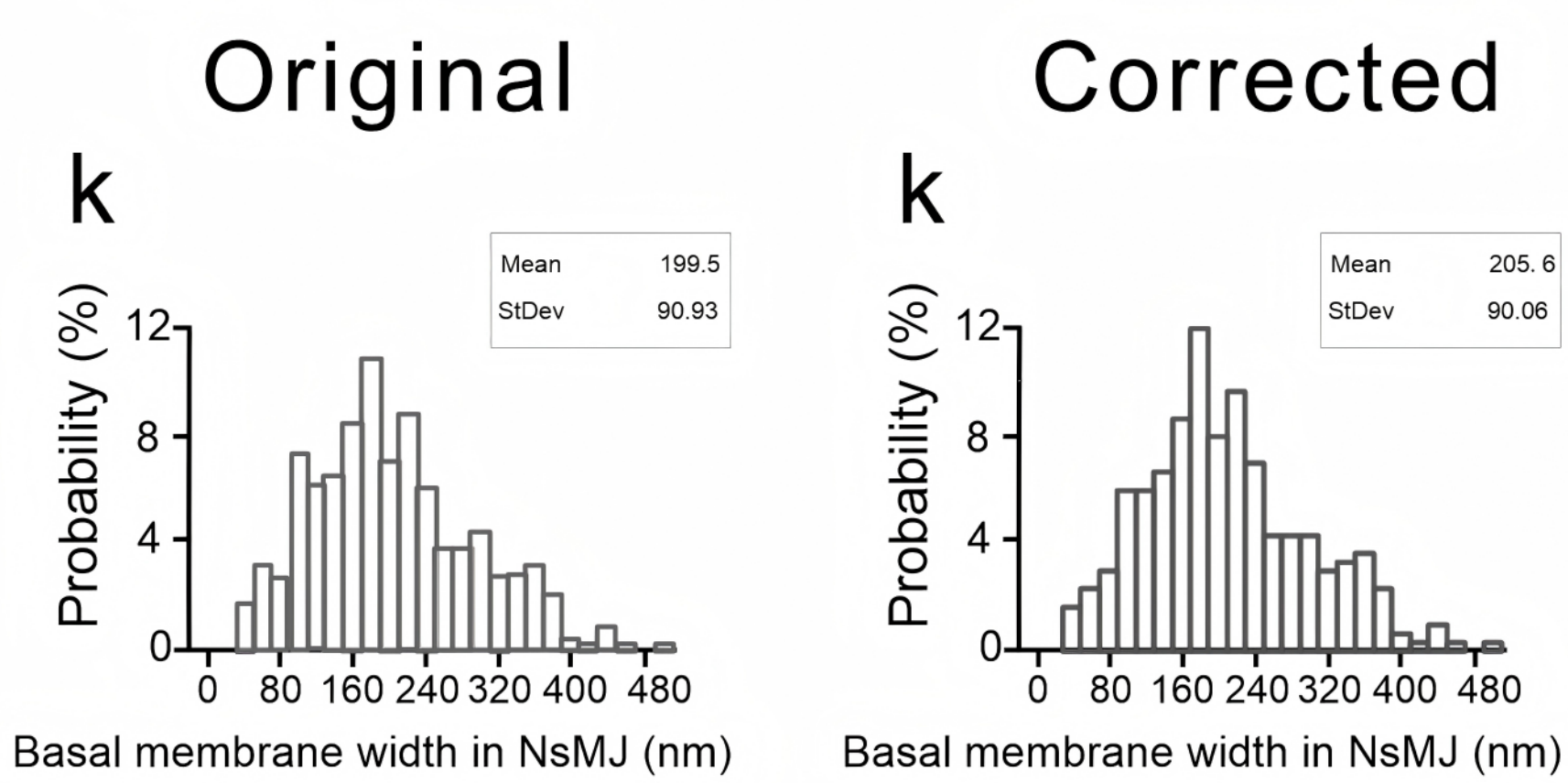

Fig. 8

Original

d

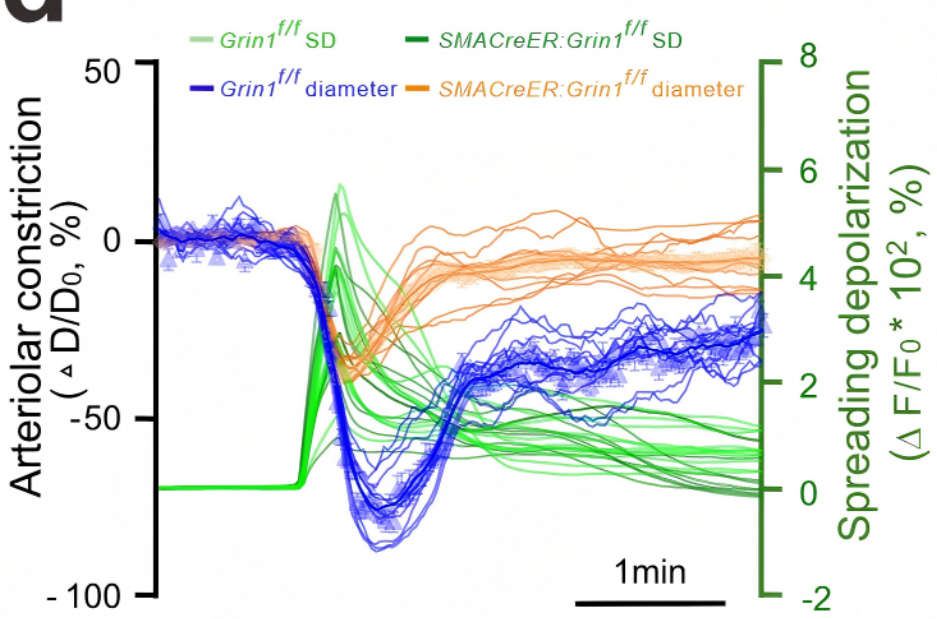

Corrected

d

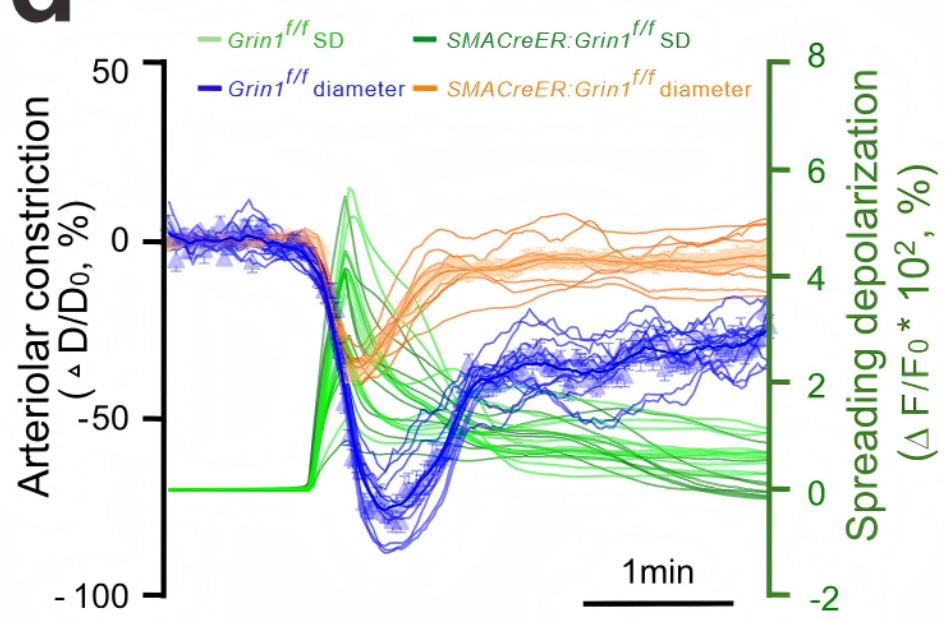

Supplement: Supplementary file 1 [file 41593_2026_2474_MOESM1_ESM.pdf]
